# Supplementary material for: Ser/Leu-swapped cell-free translation system constructed with natural/in vitro transcribed-hybrid tRNA set
Source: Nat Commun. 2024 May 16;15:4143. doi: 10.1038/s41467-024-48056-z (PMC11099018; doi:10.1038/s41467-024-48056-z)
Supplement: Supplementary file 1 — Supplementary information [file 41467_2024_48056_MOESM1_ESM.pdf]

Ser/Leu-swapped cell-free translation system constructed with natural/*in vitro* transcribed-hybrid tRNA set

Fujino *et al.*

Supplementary Method 1. Preparation of the mRNAs used in this study.
M1 mRNAs (Fig. 3a) and streptavidin mRNAs used for native-PAGE analysis (*stv-Std* and *stv-SL*, Supplementary Data 2) were prepared in our previous work[1]. The detailed protocol is as follows.

DNA templates for M1 mRNAs were prepared by extension of annealed oligonucleotides, T7esD6MYYY.F55 (5′-TAATACGACTCACTATAGGGTTAACTTTAACAAGGAGAAAAACATGTACTACTAC-3′) and Y3xDDRD_nnnXaa.R44 (Sequences are listed in Supplementary Data 3), followed by amplification by PCR using T7ex5.F22 (5′-GGCGTAATACG ACTCACTATAG-3′) and DRDuaaAS.R20 (5′-TGCTGGCACTGATTCGAAGC-3′) as primers. Extension (20 μL) was performed under the following condition: 1 × PfSH buffer [10 mM Tris-HCl (pH 8.4), 100 mM KCl, 0.1% (v/v) Triton X-100, 2 mM $MgSO_4$], 0.2 mM of each dNTP, 1 μM each forward and reverse oligonucleotides, and 2 nM of Pfu-S DNA polymerase. DNA was extended by heating the mixture at 95°C for 1 min, and 5 cycles of 50°C for 1 min and 72 °C for 1 min. PCR (200 μL) was performed under the following conditions: 1 × PfSH buffer, 0.2 mM each dNTP, 2% DMSO, 1 μM each forward and reverse primer, extended DNA (1 μL of extension mixture), and 2 nM Pfu-S DNA polymerase. DNA was amplified by 12 cycles at 95°C for 20 s, 50°C for 20 s, and 72°C for 30 s. The amplified DNA was used directly, without purification, for runoff *in vitro* transcription. Transcription of the mRNAs (1000 μL) was performed under the following conditions: 1 × T7 buffer [40 mM Tris-HCl pH 8.0, 1 mM spermidine, 0.01% Triton X-100], 10 mM DTT, 25 mM $MgCl_2$, 5 mM GMP, 5 mM each NTP, amplified DNA (200 μL of PCR mixture), and 0.12 μM T7 RNA polymerase at 37 °C overnight. The produced mRNAs were purified by extraction with phenol/chloroform and isopropanol precipitation followed by ethanol precipitation. The concentration of mRNA was determined using NanoDrop Spectrophotometer ND-1000 (Thermo Fisher Scientific).

Template DNAs for *stv-Std* and *stv-SL* were prepared using T7Aex.F24 (5′-GGCGTAATACGACTCACTATAGGA-3′) and StvWT.R20 (5′-ACATAGTTACTGCTGGACGG-3′) for *stv-Std* or T7Aex.F24 and StvSL.R20 (5′-ACATAGTTACTGCTGAACGG-3′) for *stv-SL* as primers. PCR (1000 μL) was performed using the following conditions: 1 × PfSH buffer, 2% DMSO, 0.2 mM each dNTP, 0.375 μM forward primer, 0.375 μM reverse primer, plasmid containing streptavidin genes, and 2 nM of Pfu-S DNA polymerase. DNA was amplified by 12 cycles at 95°C for 20 s, 50°C for 20 s, and 72°C for 1 min. The amplified DNA was purified by extraction with phenol/chloroform and isopropanol precipitation. DNA was dissolved in 100 μL of 10 mM Tris-AcOH pH7.8. The DNA template was transcribed by runoff *in vitro* transcription (1000 μL) using the following conditions: 1 × T7 buffer, 10 mM DTT, 30 mM $MgCl_2$, 5 mM each NTP, template DNA (100 μL of the above DNA solution), and 0.12 μM T7 RNA polymerase at 37 °C overnight.

Purification and measurement of the concentration of mRNAs was performed as described above.

The other mRNAs were prepared in this study by the following procedure. DNA templates for M2 mRNAs were prepared by PCR using T7ex5.F22 and pLCMS_3B2-4B.R24 (5′-CGTAGCTTATTTTACGTCCTTACC-3′) as primers. The PCR (200 µL) was performed under the following conditions: 1 × PfSH buffer, 0.2 mM each dNTP, 2% DMSO, 1 µM each forward and reverse primer, a plasmid carrying a template DNA sequence (Supplementary Data 1), and 2 nM of Pfu-S DNA polymerase. DNA was amplified by 10 cycles of 95°C for 40 s, 55°C for 40 s, and 72°C for 40 s. The amplified DNA was used directly, without purification, for run-off *in vitro* transcription. DNA templates for the M3 mRNAs were prepared using T7ex5.F22 and pLCMS_3A_pcrR.24 (5′-CGTAGCTTATTTTACGTCTTTACC-3′) as primers and a plasmid carrying a template DNA sequence (Supplementary Data 1) using similar procedure above. Transcription of the mRNA M2 and M3 (300 µL) was performed under the following conditions: 1 × T7 buffer, 10 mM DTT, 22.5 mM $MgCl_2$, 3.75 mM each NTP, amplified DNA (60 µL of PCR mixture) and 37 nM T7 RNA polymerase at 37 °C overnight. Purification and measurement of the concentration of mRNAs was performed as described above.

The genes coding model proteins (*gfp-Std-22C, gfp-SL-22C, pa-gfp-Std, pa-gfp-SL, pa-stv-Std, pa-stv-SL, pa-gal-Std, pa-gal-SL, pa-gfp-SL-RC5, pa-gfp-SL-RC9,* and *pa-gfp-SL-RC15*; Supplementary Data 2) were cloned into the pUC19 vector.

DNA templates for mRNA containing *gfp-Std-22C, gfp-SL-22C, and gfp-SL-47C* (Supplementary Data 2) were prepared by PCR using T7Aex.F24 and ASsfGFP.R20 (5′-TGACTAAGAATCCAGTATGC-3′) as primers. PCR (1000 µL) was performed under the same condition as the *stv-Std* and *stv-SL* mRNAs except primers and template plasmids. DNA was amplified by 12 cycles of 95°C for 20 s, 50°C for 20 s, and 72°C for 1 min. The resultant DNA was purified and transcribed to mRNAs under the same condition as the *stv-Std* and *stv-SL* mRNAs. Purification and measurement of the concentration of mRNAs was performed as described above.

DNA templates for mRNA containing other model proteins (*pa-gfp-Std, pa-gfp-SL, pa-stv-Std, pa-stv-SL, pa-gal-Std, pa-gal-SL, pa-gfp-SL-RC5, pa-gfp-SL-RC9,* and *pa-gfp-SL-RC15;* Supplementary Data 2) were amplified by PCR using T7ex5.F22 and ASsfGFP.R20 as primers. PCR (1000 µL) was performed under the same condition as the *stv-Std* and *stv-SL* mRNAs except primers and template plasmids. DNA was amplified by 15 cycles of 95°C for 40 s, 55°C for 40 s, and 72°C for 40 sec (5 min for *pa-gal-Std, pa-gal-SL*). The resultant DNA was purified by the same procedure as the *stv-Std* and *stv-SL* mRNAs. Transcription of the mRNAs (600 µL) was performed under the following conditions: 1 × T7 buffer, 10 mM DTT, 22.5 mM $MgCl_2$,

3.75 mM each NTP, amplified DNA (60 µL of the purified DNA) and 37 nM T7 RNA polymerase at 37 °C overnight. Purification and measurement of the concentration of mRNAs was performed as described above.

Supplementary Method 2. Preparation of the IVT-tRNAs used in this study.

DNA templates for the tRNAs were prepared by extension of the oligonucleotides listed in Supplementary Data 4 (see Supplementary Data 5 for sequences) followed by two rounds of PCR using the primers listed in Supplementary Data 4 (see Supplementary Data 5 for sequences). Extension (20 µL) was performed under the same condition as the M1 mRNAs except oligonucleotides. First round PCR (100 µL) was performed under the following conditions: 1 × PfSH buffer, 0.2 mM each dNTP, 2% DMSO, 1 µM each forward and reverse primer, extended DNA (2.5 µL of extension mixture), and 2 nM of *Pfu-S* DNA polymerase. DNA was amplified by 5 cycles of 95°C for 20 s, 50°C for 20 s, and 72°C for 30 s. Second round PCR (200 µL) was performed under the following conditions: 1 × PfSH buffer, 0.2 mM each dNTP, 2% DMSO, 1 µM each forward and reverse primer, amplified DNA (5% v/v of first PCR mixture), and 2 nM of *Pfu-S* DNA polymerase. DNA was amplified by 12 cycles of 95°C for 20 s, 50°C for 20 s, and 72°C for 30 s. The amplified DNA was used directly, without purification, for run-off *in vitro* transcription. Transcription of the tRNAs except $tRNA^{Trp}$, $tRNA^{Gln}$, and $tRNA^{Pro}$ (2 mL) was performed under the following conditions: 1 × T7 buffer, 10 mM DTT, 22.5 mM $MgCl_2$, 5 mM GMP, 3.75 mM each NTP, amplified DNA (200 µL of second PCR mixture) and 0.12 µM T7 RNA polymerase at 37 °C overnight. DNase I (0.005 U/µL) and $MnCl_2$ (2 mM) were added to the transcription mixture, and then the mixture was incubated at 37 °C for 2 h. After DNase I treatment, the synthesized tRNAs were purified by extraction with phenol/chloroform and two rounds of isopropanol precipitation. The tRNAs were dissolved in ultrapure water, and their concentrations were determined using a NanoDrop-spectrophotometer ND-1000. Transcription of $tRNA^{Trp}$, $tRNA^{Gln}$, and $tRNA^{Pro}$ (2 mL) was performed under the following conditions: 1 × T7 buffer, 10 mM DTT, 22.5 mM $MgCl_2$, 3.75 mM each NTP, amplified DNA (200 µL of second PCR mixture), RNase P (0.4 µM RNase P for $tRNA^{Gln}$ and $tRNA^{Pro}$, 0.8 µM RNase P for $tRNA^{Trp}$), and 0.12 µM T7 RNA polymerase at 37 °C overnight. DNase I treatment and purification of tRNAs was performed as described above. The resulting tRNA sequences are listed in Supplementary Table 5. Twenty-one different T7-tRNAs ($tRNA^{Ala}_{GGC}$, $tRNA^{Arg}_{GCG}$, $tRNA^{Asn}_{GUU}$, $tRNA^{Asp}_{GUC}$, $tRNA^{Cys}_{GCA}$, $tRNA^{Glu}_{CUC}$, $tRNA^{Gly}_{GCC}$, $tRNA^{His}_{GUG}$, $tRNA^{Ile}_{GAU}$, $tRNA^{Leu}_{GGA}$, $tRNA^{Lys}_{CUU}$, $tRNA^{ini}_{CAU}$, $tRNA^{Met}_{CAU}$, $tRNA^{Phe}_{GAA}$, $tRNA^{Ser}_{GAG}$, $tRNA^{Thr}_{GGU}$, $tRNA^{Tyr}_{GUA}$, $tRNA^{Val}_{GAC}$, $tRNA^{Gln}_{CUG}$, $tRNA^{Pro}_{GGG}$, and $tRNA^{Trp}_{CCA}$) were combined to prepare the IVT21-SL tRNA set.

Supplementary Method 3. Extraction of natural tRNAs from Rosetta™ 2 (DE3) cells.

Total tRNA was extracted from *E. coli* strain Rosetta™ 2 (DE3) cells using the following procedure.[2] The *E. coli* Rosetta™ 2 (DE3) strain was cultivated in 330 mL of LB medium with

20 μg/mL chloramphenicol and grown at 37˚C. After the culture reached an A_{600} value of 1.1, the cells were pelleted and resuspended in 4 mL of Standard buffer [0.001 M Tris-HCl (pH 7.2), 0.01 M $MgCl_2$]. Then, 4 mL of Phenol saturated with Standard buffer was added to the cells, and the solution was mixed on ice for one hour. After centrifugation at 15,000 g for 20 minutes at 4˚C, the aqueous phase was collected. To this, 500 μL of cooled 20% (w/w) AcOK (pH 5.5) was added, followed by 11 mL of ethanol, and the mixture was incubated at –20˚C for 30 minutes. After centrifugation at 15,000 g for 10 minutes at 4˚C, the pellet was dissolved in 4 mL of 1 M NaCl solution. This solution was centrifuged again under the same conditions, and the resulting pellet was dissolved in 2 mL of 1 M NaCl. After a third centrifugation, the supernatants from the 4 mL and 2 mL solutions were combined. Following the addition of 6.1 mL of ethanol and subsequent centrifugation, the pellet was dissolved in 816 μL of 0.5 M Tris-HCl (pH 8.8), and incubated at 37˚C for one hour. Next, 81 μL of 20% (w/w) AcOK (pH 5.5) and 1.8 mL of ethanol were added. After centrifugation, the pellet was dissolved in 2.5 mL of 0.3 M AcONa (pH 7), and 1.5 mL of isopropanol was added. Post-centrifugation, 4 mL of supernatant was collected, and the pellet was again dissolved in 1.3 mL of 0.3 M AcONa (pH 7). After 0.7 mL of isopropanol was added, the solution was centrifuged, and 2 mL of supernatant was collected. The supernatants (total 6 mL) were mixed and 5.9 mL of isopropanol was added. Another round of centrifugation was performed, and the final pellet was resuspended in 70% ethanol and mixed for one hour at 25˚C. After the last centrifugation, the pellet was air-dried and then dissolved in 50 μL of ultra-pure water.

Supplementary Method 4. LC-MS analysis of synthesized peptides and proteins.

For the peptide purification, 10 µL of methanol was added to 10 µL of the translation reaction mixture and the precipitate was removed by centrifugation (15,000 g, 10 min). Then, 20 µL of 1% TFA was added to the supernatant and the precipitate was removed by centrifugation (15,000 g, 10 min). The resulting solution was used for the LC-MS analysis.

For protein sample purification, 10 µL of the translation mixture was added to 10 µL of Anti PA tag antibody beads. After incubating for one hour at 25 °C, beads were washed twice with 25 mM Hepes-K (pH 7.5), 150 mM NaCl, 0.05% Tween20 solution, and once with ultrapure water. The proteins on the beads were digested with Lys-C (0.5 µg) at 37 °C for 4 hours followed by digestion with trypsin (0.5 µg) at 37 °C overnight. The protein lysates were incubated for 15 min at 37°C in 25 mM TCEP and alkylated for 30 min at 37°C in the dark using 25 mM iodoacetamide.

Model peptides and the digested protein fragments were desalted using GL-Tip SDB (GL Sciences), evaporated in a SpeedVac concentrator, and redissolved in 0.1% TFA and 2% acetonitrile. Nanoscale liquid chromatography coupled with tandem mass spectrometry (nano LC-MS/MS) was performed using Dionex U3000 gradient pump (Thermo Fisher Scientific)

coupled with Q Exactive Hybrid Quadrupole-Orbitrap Mass Spectrometer (Thermo Fisher Scientific). The samples were concentrated on a C18 trap column (5-μm particle size, 300 μm inner diameter, 5 mm length; Chemical Evaluation and Research Institute, Tokyo, Japan) and separated on a C18 column (3-μm particle size, 100-μm inner diameter, 125 mm length; Nikkyo Technos) at a flow rate of 0.5 μL/min. The mobile phases were solvent A (0.5% acetic acid) and solvent B (0.5% acetic acid in 80% acetonitrile). For protein analysis, the elution gradient for solvent B was as follows: 5% to 40% B over 100 min, then 40% B to 95% B for 1 min, 95% B for 5 min, then back to 5% B over 1 min, and finally re-equilibrating at 5% B for 8 min. For peptide analysis, the elution gradient for solvent B was as follows: 5% to 40% B over 20 min, then 40% B to 95% B for 1 min, 95% B for 5 min, then back to 5% B over 1 min, and finally re-equilibrating at 5% B for 8 min. Electrospray ionization was performed in positive-ion mode. Xcalibur 4.1.50 (Thermo Fisher Scientific) was used to record peptide spectra. The full scan was acquired from 350 to 1800 m/z with a resolution of 17,500, automatic gain control (AGC) as 3×10^6, and maximum injection time as 60 ms. MS/MS scans were performed with a resolution of 35,000, AGC target as 1×10^5, and maximum injection time as 60 ms. The 10 highest intensity precursor ions were isolated using the quadrupole analyzer in a window of 2.0 m/z and fragmented by higher energy collisional dissociation (HCD) fragmentation with normalized collision energy (NCE) of 27%. Multiply-charged peptides were chosen for MS/MS experiments. Dynamic exclusion time was set to 5 s for peptide analysis and 10 s for protein analysis.

For the analysis of sequence data, FASTA files listed in Supplementary Data 6 were used. MS/MS spectra were interpreted and peak lists were generated using Proteome Discoverer 2.4.1.15 (Thermo Fisher Scientific). Searches were performed using SEQUEST (Thermo Fisher Scientific) with the following parameters: a mass tolerance of 10 ppm for peptide tolerance, 0.02 Da for MS/MS tolerance, a fixed modification of carbamidomethyl (C) or formyl (any N-terminus) and a variable modification of oxidation (M). Peptide identifications were based on a significant Xcorr values (high confidence filter). Peptide identification information returned from SEQUEST was filtered at a false discovery rate (FDR) of 1% using the Target Decoy PSM Validator node of Proteome Discoverer to obtain confirmed peptide identification and modification lists of HCD MS/MS. LC-MS/MS analysis was performed once (n = 1) for each sample (Total 46 samples). Only peptides identified by LC-MS/MS were discussed in the manuscript.

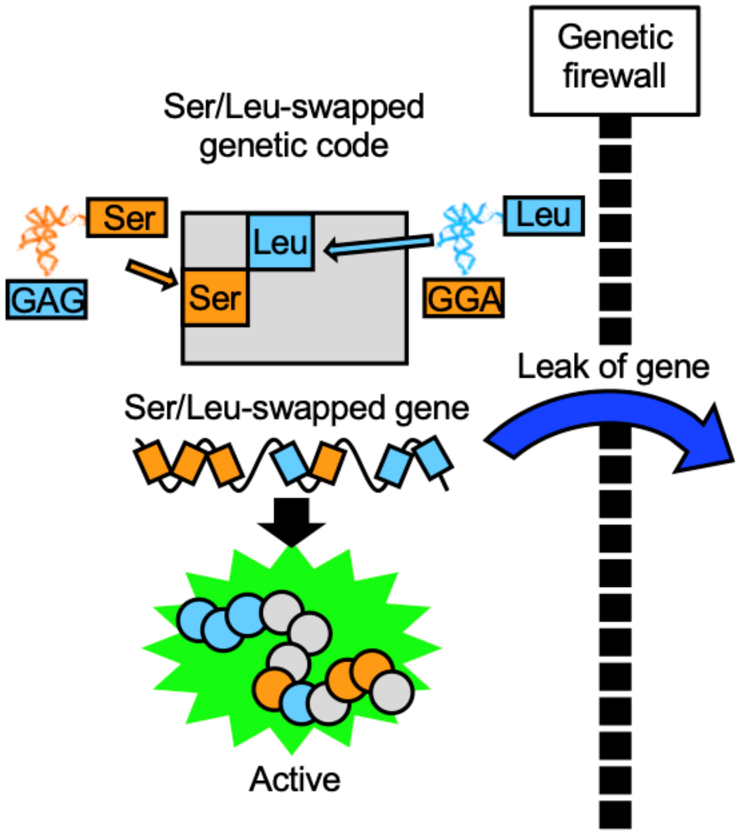

Supplementary Fig. 1. Concept for preventing the expression of the functional proteins from leaked genes using the Ser/Leu-swapped genetic code. In the translation system employing the Ser/Leu-swapped genetic code, Ser is assigned to Leu codons and vice versa, by utilizing anticodon-swapped chimeric tRNAs. A gene encoded by this Ser/Leu-swapped code is translated into a functional protein only within this specific translation system. If this gene were to leak into the environment and be assimilated by natural organisms, it would translate into a non-functional protein due to the Ser/Leu-swap. Thus, the Ser/Leu-swapped genetic code acts as a genetic firewall, reducing potential biohazards.

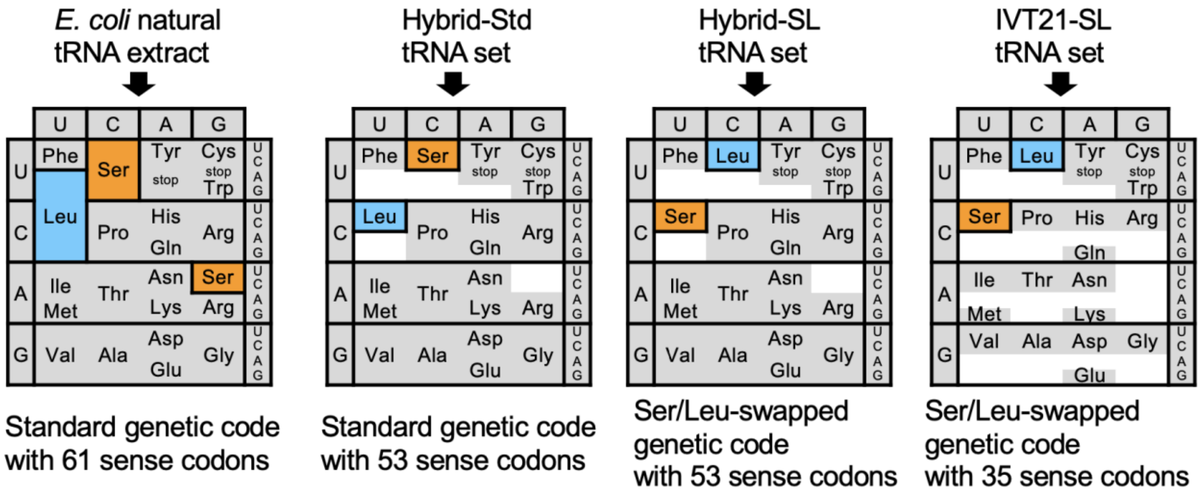

Supplementary Fig. 2. The four genetic codes produced by respective tRNA sets in this study. Abbreviations: *E. coli*, *Escherichia coli*; IVT, *in vitro* transcribed; Std, standard; SL, Ser/Leu-swapped.

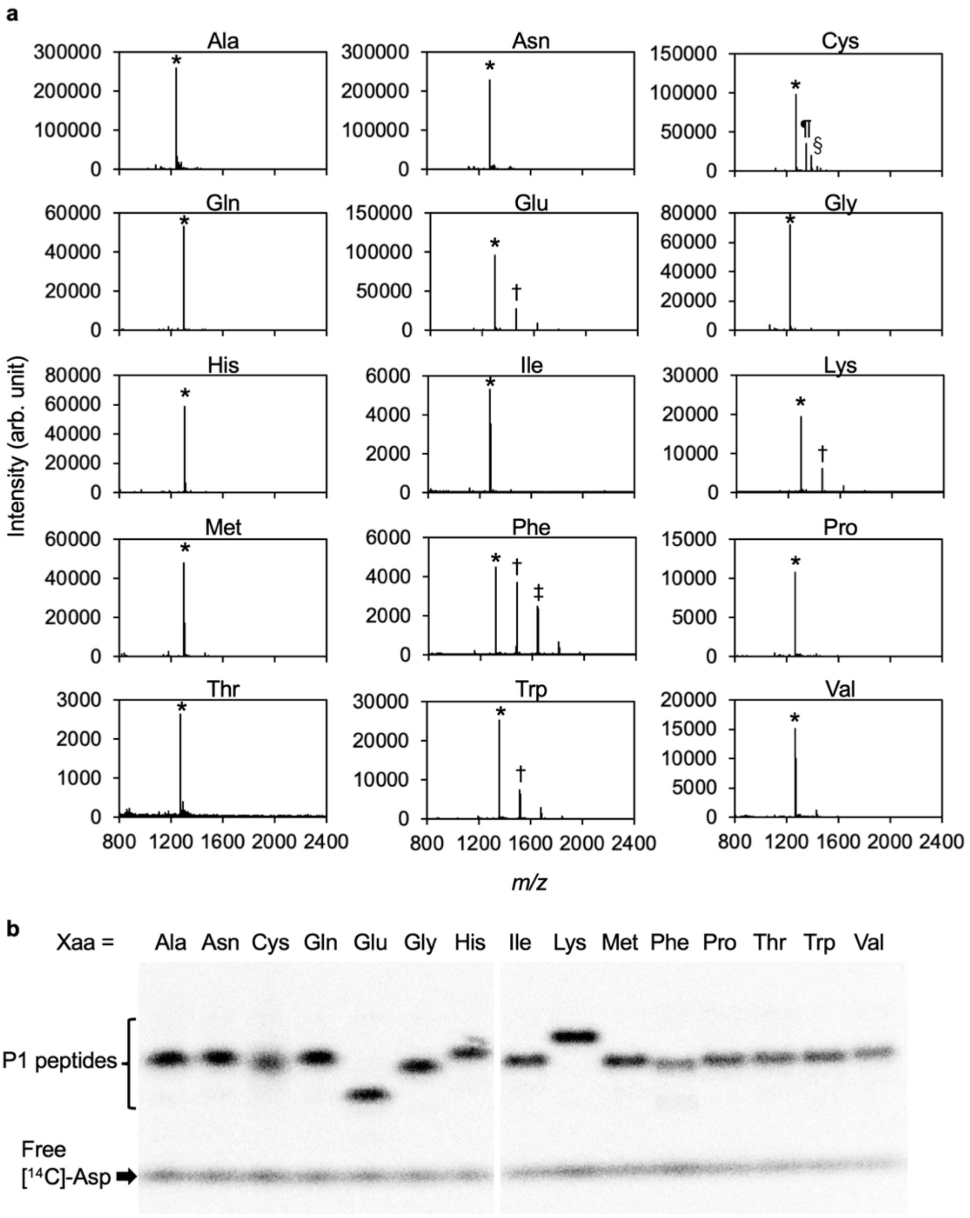

Supplementary Fig. 3. Translation of model peptides using the cell-free translation system with the hybrid-Std tRNA set. The sequences of the model peptides and the corresponding mRNAs are shown in Fig. 3a. Within the sequence, Xaa denotes 15 of the 20 natural amino acids, excluding Tyr, Asp, Arg, Ser, and Leu. **a** MALDI-TOF-MS analysis of the model peptides. Peak labels are as follows: full-length model peptide with the intended amino acid at the position Xaa, asterisk; full-length model peptide with adducts of β-mercaptoethanol, pilcrow; full-length peptide with Cys monomer, section sign; full-length peptide +163 by-product corresponding to an added Tyr, dagger; full-length peptide +326 by-product corresponding to two additional Tyr residues, double dagger. The calculated and observed masses of the peptides are provided in Supplementary Table 2. **b** Tricine SDS-PAGE analysis of the model peptides. The model peptides were analyzed by tricine SDS-PAGE and detected by the autoradiography (n = 1). Source data are provided as a Source Data file.

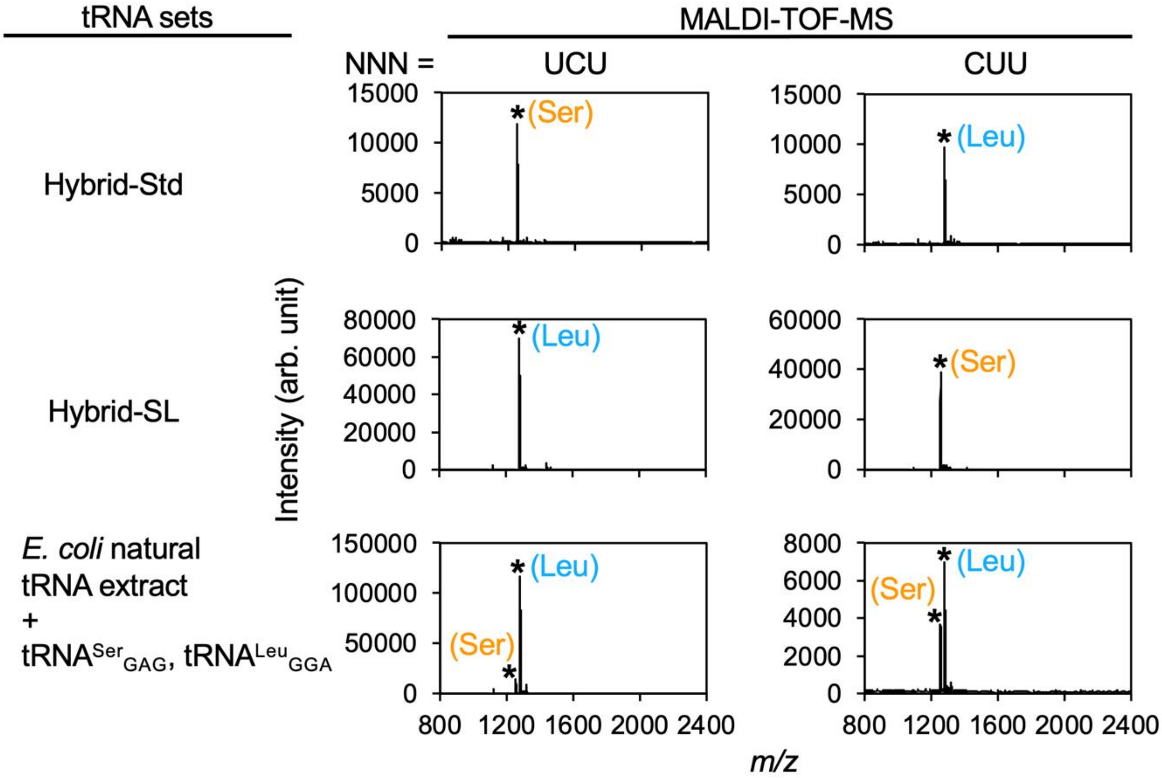

Supplementary Fig. 4. Full spectra of the MALDI-TOF-MS analysis shown in Fig. 3b. Source data are provided as a Source Data file.

```
                     Met Asn Ser Lys Gly Glu Glu Leu Phe Thr Gly Val Val Pro Ile Leu Val Glu Leu Asp
gfp-Std-22C      1   ATG AAT TCT AAG GGT GAG GAG CTT TTC ACT GGT GTT GTT CCT ATC CTT GTT GAG CTT GAT   60
gfp-SL-22C       1   ATG AAT CTT AAG GGT GAG GAG TCT TTC ACT GGT GTT GTT CCT ATC TCT GTT GAG TCT GAT   60
gfp-SL-47C       1   ATG --- CTT AAA GGC GAA GAG TCT TTT ACG GGT GTC GTC CCC ATC TCT GTC GAA TCT GAT   60

                     Gly Asp Val Asn Gly His Lys Phe Ser Val Arg Gly Glu Gly Glu Gly Asp Ala Thr Asn
gfp-Std-22C     61   GGT GAT GTT AAC GGT CAT AAG TTC TCT GTT CGT GGT GAG GGT GAG GGT GAT GCT ACT AAC  120
gfp-SL-22C      61   GGT GAT GTT AAC GGT CAT AAG TTC CTT GTT CGT GGT GAG GGT GAG GGT GAT GCT ACT AAC  120
gfp-SL-47C      61   GGT GAC GTA AAT GGC CAC AAA TTT CTT GTA CGT GGA GAA GGC GAA GGT GAT GCG ACC AAT  120

                     Gly Lys Leu Thr Leu Lys Phe Ile Cys Thr Thr Gly Lys Leu Pro Val Pro Trp Pro Thr
gfp-Std-22C    121   GGT AAG CTT ACT CTT AAG TTC ATC TGT ACT ACT GGT AAG CTT CCT GTT CCT TGG CCT ACT  180
gfp-SL-22C     121   GGT AAG TCT ACT TCT AAG TTC ATC TGT ACT ACT GGT AAG TCT CCT GTT CCT TGG CCT ACT  180
gfp-SL-47C     121   GGG AAA TCT ACC TCT AAA TTC ATC TGT ACA ACC GGA AAA TCT CCG GTT CCA TGG CCT ACG  180

                     Leu Val Thr Thr Leu Thr Tyr Gly Val Gln Cys Phe Ser Arg Tyr Pro Asp His Met Lys
gfp-Std-22C    181   CTT GTT ACT ACT CTT ACT TAC GGT GTT CAG TGT TTC TCT CGT TAC CCT GAT CAT ATG AAG  240
gfp-SL-22C     181   TCT GTT ACT ACT TCT ACT TAC GGT GTT CAG TGT TTC CTT CGT TAC CCT GAT CAT ATG AAG  240
gfp-SL-47C     181   TCT GTT ACG ACA TCT ACG TAT GGC GTT CAA TGC TTT CTT CGG TAT CCG GAT CAT ATG AAG  240

                     Arg His Asp Phe Phe Lys Ser Ala Met Pro Glu Gly Tyr Val Gln Glu Arg Thr Ile Ser
gfp-Std-22C    241   CGT CAT GAT TTC TTC AAG TCT GCT ATG CCT GAG GGT TAC GTT CAG GAG CGT ACT ATC TCT  300
gfp-SL-22C     241   CGT CAT GAT TTC TTC AAG CTT GCT ATG CCT GAG GGT TAC GTT CAG GAG CGT ACT ATC CTT  300
gfp-SL-47C     241   CGT CAT GAC TTC TTC AAA CTT GCG ATG CCT GAA GGG TAT GTG CAG GAA CGC ACC ATC CTT  300

                     Phe Lys Asp Asp Gly Thr Tyr Lys Thr Arg Ala Glu Val Lys Phe Glu Gly Asp Thr Leu
gfp-Std-22C    301   TTC AAG GAT GAT GGT ACT TAC AAG ACT CGT GCT GAG GTT AAG TTC GAG GGT GAT ACT CTT  360
gfp-SL-22C     301   TTC AAG GAT GAT GGT ACT TAC AAG ACT CGT GCT GAG GTT AAG TTC GAG GGT GAT ACT TCT  360
gfp-SL-47C     301   TTC AAA GAC GAT GGC ACG TAT AAA ACC CGT GCA GAA GTG AAA TTT GAG GGC GAT ACC TCT  360

                     Val Asn Arg Ile Glu Leu Lys Gly Ile Asp Phe Lys Glu Asp Gly Asn Ile Leu Gly His
gfp-Std-22C    361   GTT AAC CGT ATC GAG CTT AAG GGT ATC GAT TTC AAG GAG GAT GGT AAC ATC CTT GGT CAT  420
gfp-SL-22C     361   GTT AAC CGT ATC GAG TCT AAG GGT ATC GAT TTC AAG GAG GAT GGT AAC ATC TCT GGT CAT  420
gfp-SL-47C     361   GTG AAT CGC ATT GAA TCT AAA GGC ATC GAC TTC AAA GAA GAT GGC AAC ATT TCT GGT CAC  420

                     Lys Leu Glu Tyr Asn Phe Asn Ser His Asn Val Tyr Ile Thr Ala Asp Lys Gln Lys Asn
gfp-Std-22C    421   AAG CTT GAG TAC AAC TTC AAC TCT CAT AAC GTT TAC ATC ACT GCT GAT AAG CAG AAG AAC  480
gfp-SL-22C     421   AAG TCT GAG TAC AAC TTC AAC CTT CAT AAC GTT TAC ATC ACT GCT GAT AAG CAG AAG AAC  480
gfp-SL-47C     421   AAG TCT GAG TAC AAC TTT AAC CTT CAT AAC GTG TAC ATT ACT GCC GAT AAA CAG AAG AAC  480

                     Gly Ile Lys Ala Asn Phe Lys Ile Arg His Asn Val Glu Asp Gly Ser Val Gln Leu Ala
gfp-Std-22C    481   GGT ATC AAG GCT AAC TTC AAG ATC CGT CAT AAC GTT GAG GAT GGT TCT GTT CAG CTT GCT  540
gfp-SL-22C     481   GGT ATC AAG GCT AAC TTC AAG ATC CGT CAT AAC GTT GAG GAT GGT CTT GTT CAG TCT GCT  540
gfp-SL-47C     481   GGT ATC AAA GCC AAC TTT AAG ATT CGC CAT AAT GTG GAA GAT GGC CTT GTG CAG TCT GCT  540

                     Asp His Tyr Gln Gln Asn Thr Pro Ile Gly Asp Gly Pro Val Leu Leu Pro Asp Asn His
gfp-Std-22C    541   GAT CAT TAC CAG CAG AAC ACT CCT ATC GGT GAT GGT CCT GTT CTT CTT CCT GAT AAC CAT  600
gfp-SL-22C     541   GAT CAT TAC CAG CAG AAC ACT CCT ATC GGT GAT GGT CCT GTT TCT TCT CCT GAT AAC CAT  600
gfp-SL-47C     541   GAC CAT TAC CAG CAG AAC ACT CCG ATT GGG GAT GGT CCG GTA TCT TCT CCA GAC AAT CAC  600

                     Tyr Leu Ser Thr Gln Ser Val Leu Ser Lys Asp Pro Asn Glu Lys Arg Asp His Met Val
gfp-Std-22C    601   TAC CTT TCT ACT CAG TCT GTT CTT TCT AAG GAT CCT AAC GAG AAG CGT GAT CAT ATG GTT  660
gfp-SL-22C     601   TAC TCT CTT ACT CAG CTT GTT TCT CTT AAG GAT CCT AAC GAG AAG CGT GAT CAT ATG GTT  660
gfp-SL-47C     601   TAC TCT CTT ACC CAA CTT GTT TCT CTT AAA GAC CCG AAT GAG AAA CGC GAT CAC ATG GTG  660

                     Leu Leu Glu Phe Val Thr Ala Ala Gly Ile Thr His Gly Met Asp Glu Leu Tyr Lys stop
gfp-Std-22C    661   CTT CTT GAG TTC GTT ACT GCT GCT GGT ATC ACT CAT GGT ATG GAT GAG CTC TAC AAG TAA  720
gfp-SL-22C     661   TCT TCT GAG TTC GTT ACT GCT GCT GGT ATC ACT CAT GGT ATG GAT GAG TCC TAC AAG TAA  720
gfp-SL-47C     661   TCT TCT GAA TTT GTC ACT GCA GCG GGT ATT ACC CAT GGT ATG GAT GAG TCT TAT AAG TAA  720


gfp-Std-22C          GCA TAC TGG ATT CTT AGT  CA
gfp-SL-22C           GCA TAC TGG ATT CTT AGT  CA
gfp-SL-47C           GCA TAC TGG ATT CTT AGT  CA
```

gfp-Std-22C

Phe	UUU	0	Ser	UCU	10	Tyr	UAU	0	Cys	UGU	2
	UUC	12		UCC	0		UAC	9		UGC	0
Leu	UUA	0		UCA	0	stop	UAA	1	stop	UGA	0
	UUG	0		UCG	0		UAG	0	Trp	UGG	1
	CUU	19	Pro	CCU	10	His	CAU	10	Arg	CGU	8
	CUC	1		CCC	0		CAC	0		CGC	0
	CUA	0		CCA	0	Gln	CAA	0		CGA	0
	CUG	0		CCG	0		CAG	7		CGG	0
Ile	AUU	0	Thr	ACU	18	Asn	AAU	1	Ser	AGU	0
	AUC	11		ACC	0		AAC	13		AGC	0
	AUA	0		ACA	0	Lys	AAA	0	Arg	AGA	0
Met	AUG	5		ACG	0		AAG	20		AGG	0
Val	GUU	18	Ala	GCU	8	Asp	GAU	18	Gly	GGU	22
	GUC	0		GCC	0		GAC	0		GGC	0
	GUA	0		GCA	0	Glu	GAA	0		GGA	0
	GUG	0		GCG	0		GAG	16		GGG	0

gfp-SL-22C

Phe	UUU	0	Ser	UCU	19	Tyr	UAU	0	Cys	UGU	2
	UUC	12		UCC	1		UAC	9		UGC	0
Leu	UUA	0		UCA	0	stop	UAA	1	stop	UGA	0
	UUG	0		UCG	0		UAG	0	Trp	UGG	1
	CUU	10	Pro	CCU	10	His	CAU	10	Arg	CGU	8
	CUC	0		CCC	0		CAC	0		CGC	0
	CUA	0		CCA	0	Gln	CAA	0		CGA	0
	CUG	0		CCG	0		CAG	7		CGG	0
Ile	AUU	0	Thr	ACU	18	Asn	AAU	1	Ser	AGU	0
	AUC	11		ACC	0		AAC	13		AGC	0
	AUA	0		ACA	0	Lys	AAA	0	Arg	AGA	0
Met	AUG	5		ACG	0		AAG	20		AGG	0
Val	GUU	18	Ala	GCU	8	Asp	GAU	18	Gly	GGU	22
	GUC	0		GCC	0		GAC	0		GGC	0
	GUA	0		GCA	0	Glu	GAA	0		GGA	0
	GUG	0		GCG	0		GAG	16		GGG	0

gfp-SL-47C

Phe	UUU	7	Ser	UCU	20	Tyr	UAU	5	Cys	UGU	1
	UUC	5		UCC	0		UAC	4		UGC	1
Leu	UUA	0		UCA	0	stop	UAA	1	stop	UGA	0
	UUG	0		UCG	0		UAG	0	Trp	UGG	1
	CUU	10	Pro	CCU	2	His	CAU	6	Arg	CGU	3
	CUC	0		CCC	1		CAC	4		CGC	4
	CUA	0		CCA	2	Gln	CAA	2		CGA	0
	CUG	0		CCG	5		CAG	5		CGG	1
Ile	AUU	6	Thr	ACU	3	Asn	AAU	6	Ser	AGU	0
	AUC	5		ACC	8		AAC	7		AGC	0
	AUA	0		ACA	2	Lys	AAA	15	Arg	AGA	0
Met	AUG	5		ACG	5		AAG	5		AGG	0
Val	GUU	4	Ala	GCU	1	Asp	GAU	11	Gly	GGU	8
	GUC	4		GCC	2		GAC	7		GGC	9
	GUA	3		GCA	2	Glu	GAA	11		GGA	2
	GUG	7		GCG	3		GAG	5		GGG	3

Supplementary Fig. 5. The sequences of *gfp-Std-22C, gfp-SL-22C*, and *gfp-SL-47C* genes and their codon usages. Ser and Leu codons are highlighted in green.

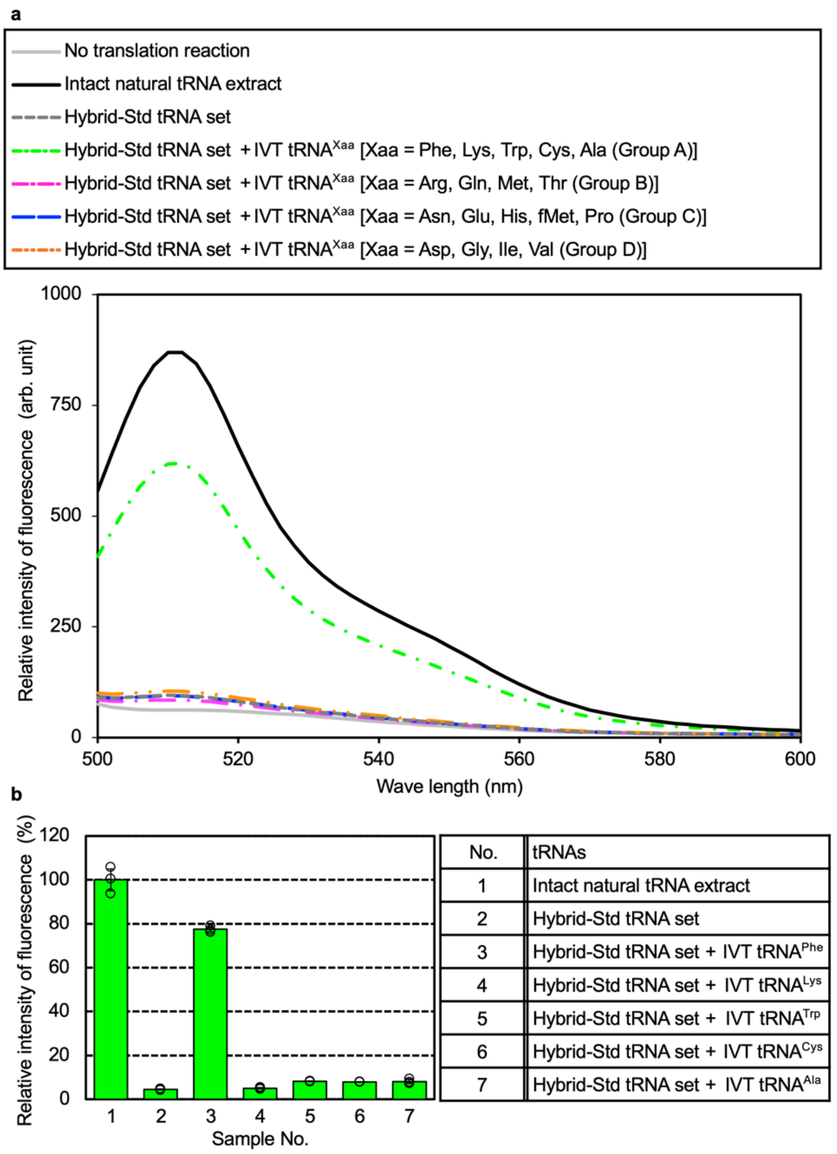

Supplementary Fig. 6. Exploration of the additional IVT tRNA(s) required for the efficient production of sfGFP. a The IVT initiator tRNA and 17 IVT tRNAs corresponding to 17 out of the 20 natural amino acids (excluding Ser, Leu, and Tyr) were divided into four groups A–D. Each group of tRNAs was added to the cell-free the translation system containing the hybrid-Std tRNA set. sfGFP was translated from the *gfp-Std-22C* gene. After the reaction, the fluorescence intensity of sfGFP in the translation systems was measured by using a spectrofluorometer (ex = 488 nm, em = 500–600 nm; n = 1). **b** Each IVT tRNA in Group A ($tRNA^{Phe}$, $tRNA^{Lys}$, $tRNA^{Trp}$, $tRNA^{Cys}$, and $tRNA^{Ala}$) was added to the translation system with the hybrid-Std tRNA set. sfGFP was translated from the *gfp-Std-22C* gene. The fluorescence intensity of sfGFP in the translation systems was measured using a spectrofluorometer (ex = 488 nm, em = 510 nm). The relative fluorescence intensity of sfGFP was normalized against the fluorescence intensity with the natural tRNA extract. Bars represent mean ± SD, and open circles represent individual data points for n = 3 biologically independent experiments. Abbreviations: Std, standard; IVT, *in vitro* transcribed. Source data are provided as a Source Data file.

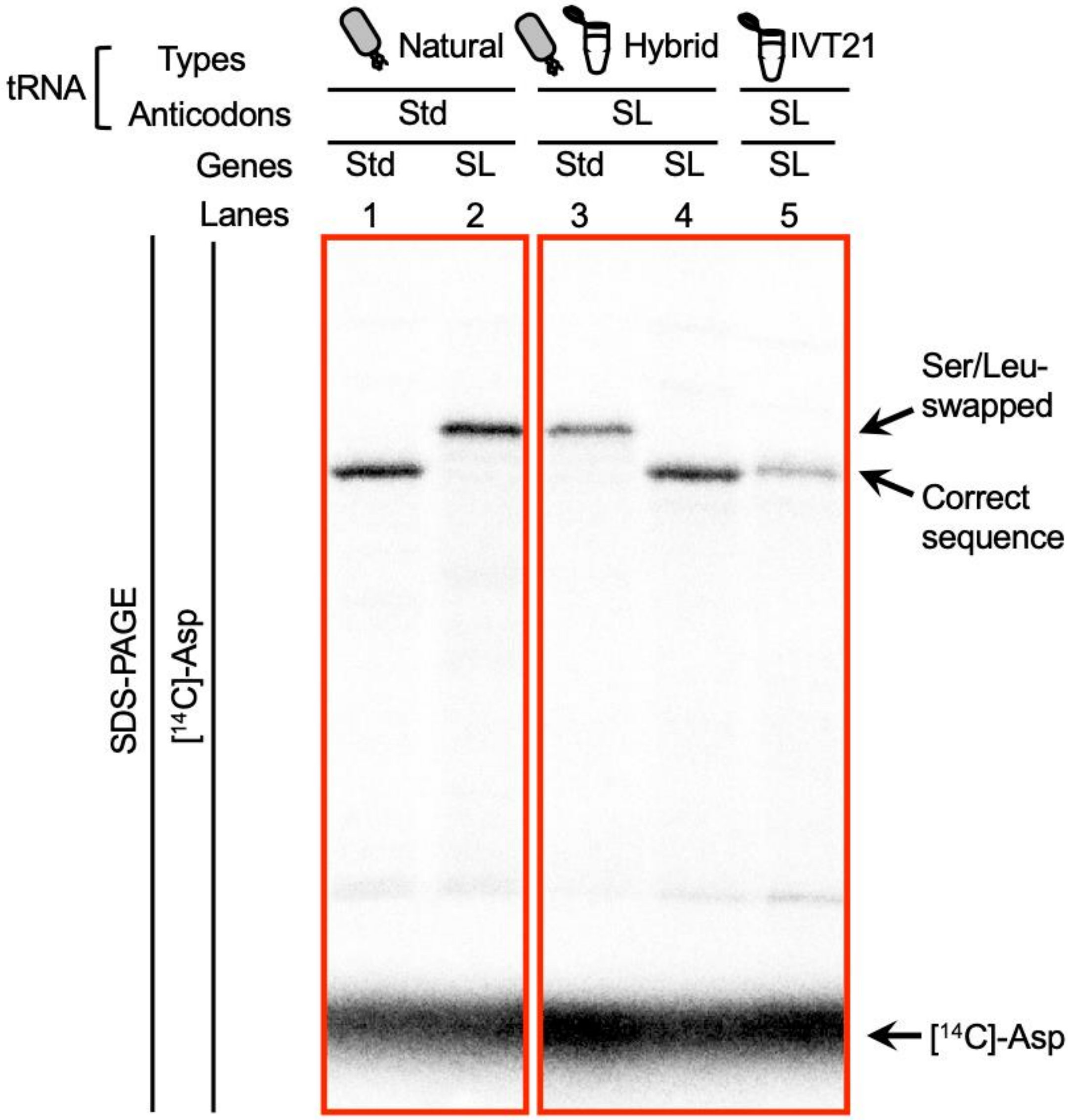

Supplementary Fig. 7. SDS-PAGE analysis of the sfGFP shown in Fig. 4a. The proteins were detected by the autoradiography. Cell-free translation systems were constructed using the tRNA sets, and two sfGFP genes coded with the standard genetic code (*gfp-Std-22C gene*) or the Ser/Leu-swapped genetic code (*gfp-SL-22C gene*) were translated. Samples were run on the same gel, and the image was cropped only for the purpose of this figure. Gel image is representative of n = 3 biologically independent experiments. Abbreviations: UTR, untranslated region; Std, standard; SL, Ser/Leu-swapped. Source data are provided as a Source Data file.

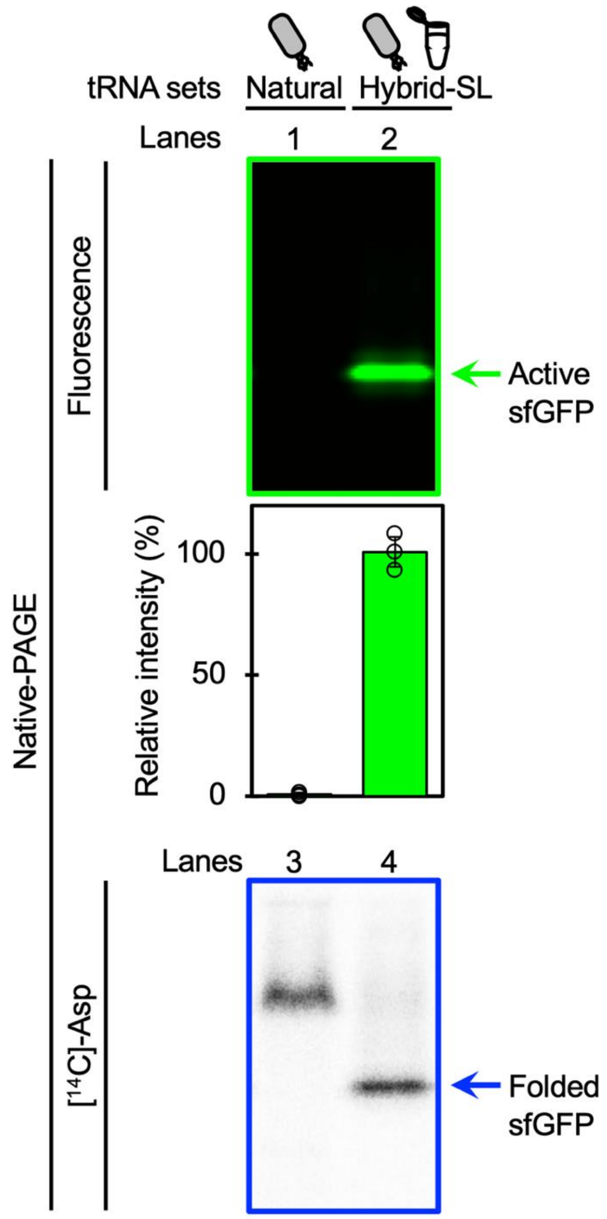

Supplementary Fig. 8. Translation of the *gfp-SL-47C* gene using the natural tRNA extract or the hybrid-SL tRNA set. The upper panel shows the result of native PAGE followed by the fluorescence imaging of the sfGFP. The bottom panel displays the autoradiography result of the same gel. Gel images were cropped only for the purpose of this figure. Gel images are representative of n = 3 biologically independent experiments. Bars represent mean ± SD, and open circles represent individual data points for n = 3 biologically independent experiments. The relative band intensities were normalized against the intensity of the band observed with the combination of the natural tRNA extract and the *gfp-Std-22C* gene (Fig. 4a, lane 1). For quantitative comparisons between samples on different gels, same standard sample was applied on both gels. Abbreviations: SL, Ser/Leu-swapped. Source data are provided as a Source Data file.

```
                  Met Asn Met Lys Gly Val Ala Met Pro Gly Ala Glu Asp Asp Val Val Lys Leu Lys Gly
pa-gfp-SL        1 ATG AAT ATG AAA GGC GTT GCG ATG CCT GGT GCC GAA GAT GAT GTC GTG AAA CTT AAA GGC 60
pa-gfp-SL-RC5    1 ATG AAT ATG AAA GGC GTT GCG ATG CCT GGT GCC GAA GAT GAT GTC GTG AAA CTT AAA GGC 60
pa-gfp-SL-RC9    1 ATG AAT ATG AAA GGC GTT GCG ATG CCT GGT GCC GAA GAT GAT GTC GTG AAA CTT AAA GGC 60
pa-gfp-SL-RC15   1 ATG AAT ATG AAA GGC GTT GCG ATG CCT GGT GCC GAA GAT GAT GTC GTG AAA CTT AAA GGC 60

                  Glu Glu Ser Phe Thr Gly Val Val Pro Ile Ser Val Glu Ser Asp Gly Asp Val Asn Gly
pa-gfp-SL       61 GAA GAG TCT TTT ACG GGT GTC GTC CCC ATC TCT GTC GAA TCT GAT GGT GAC GTA AAT GGC 120
pa-gfp-SL-RC5   61 GAA GAA TCT TTT ACC GGT GTC GTA CCG ATT TCT GTC GAG TCT GAC GGC GAT GTG AAT GGC 120
pa-gfp-SL-RC9   61 GAA GAA TCT TTT ACC GGT GTC GTA CCG ATT TCT GTC GAG TCT GAC GGC GAT GTG AAT GGA 120
pa-gfp-SL-RC15  61 GAA GAA TCT TTT ACC GGT GTC GTA CCG ATT TCT GTC GAG TCT GAC GGC GAT GTG AAT GGA 120

                  His Lys Phe Leu Val Arg Gly Glu Gly Glu Gly Asp Ala Thr Asn Gly Lys Ser Thr Ser
pa-gfp-SL      121 CAC AAA TTT CTT GTA CGT GGA GAA GGC GAA GGT GAT GCG ACC AAT GGG AAA TCT ACC TCT 180
pa-gfp-SL-RC5  121 CAC AAG TTT CTT GTC CGT GGT GAA GGT GAA GGC GAT GCG ACC AAT GGG AAA TCT ACG TCT 180
pa-gfp-SL-RC9  121 CAC AAG TTT CTT GTC CGT GGT GAA GGT GAA GGC GAT GCG ACC AAT GGG AAA TCT ACG TCT 180
pa-gfp-SL-RC15 121 CAC AAG TTT CTT GTC CGT GGA GAA GGA GAA GGA GAT GCG ACC AAT GGG AAA TCT ACG TCT 180

                  Lys Phe Ile Cys Thr Thr Gly Lys Ser Pro Val Pro Trp Pro Thr Ser Val Thr Thr Ser
pa-gfp-SL      181 AAA TTC ATC TGT ACA ACC GGA AAA TCT CCG GTT CCA TGG CCT ACG TCT GTT ACG ACA TCT 240
pa-gfp-SL-RC5  181 AAA TTC ATC TGT ACG ACA GGC AAA TCT CCG GTA CCT TGG CCA ACT TCT GTC ACT ACG TCT 240
pa-gfp-SL-RC9  181 AAA TTC ATC TGT ACG ACA GGC AAA TCT CCG GTA CCT TGG CCA ACT TCT GTC ACT ACG TCT 240
pa-gfp-SL-RC15 181 AAA TTC ATC TGT ACG ACA GGC AAA TCT CCG GTA CCT TGG CCA ACT TCT GTC ACT ACG TCT 240

                  Thr Tyr Gly Val Gln Cys Phe Leu Arg Tyr Pro Asp His Met Lys Arg His Asp Phe Phe
pa-gfp-SL      241 ACG TAT GGC GTT CAA TGC TTT CTT CGG TAT CCG GAT CAT ATG AAG CGT CAT GAC TTC TTC 300
pa-gfp-SL-RC5  241 ACG TAT GGG GTT CAG TGC TTT CTT CGT TAT CCG GAT CAC ATG AAA CGG CAT GAC TTC TTT 300
pa-gfp-SL-RC9  241 ACG TAT GGG GTT CAG TGC TTT CTT CGG TAT CCC GAT CAC ATG AAA CGG CAT GAC TTC TTT 300
pa-gfp-SL-RC15 241 ACG TAT GGG GTT CAG TGC TTT CTT CGG TAT CCC GAT CAC ATG AAA CGG CAT GAC TTC TTT 300

                  Lys Leu Ala Met Pro Glu Gly Tyr Val Gln Glu Arg Thr Ile Leu Phe Lys Asp Asp Gly
pa-gfp-SL      301 AAA CTT GCG ATG CCT GAA GGG TAT GTG CAG GAA CGC ACC ATC CTT TTC AAA GAC GAT GGC 360
pa-gfp-SL-RC5  301 AAA CTT GCA ATG CCG GAA GGC TAT GTG CAA GAA AGA ACG ATT CTT TTT AAA GAC GAC GGT 360
pa-gfp-SL-RC9  301 AAA CTT GCA ATG CCC GAA GGC TAT GTG CAA GAA AGA ACG ATT CTT TTT AAA GAC GAC GGT 360
pa-gfp-SL-RC15 301 AAA CTT GCA ATG CCC GAA GGC TAT GTG CAA GAA AGA ACG ATA CTT TTT AAA GAC GAC GGT 360

                  Thr Tyr Lys Thr Arg Ala Glu Val Lys Phe Glu Gly Asp Thr Ser Val Asn Arg Ile Glu
pa-gfp-SL      361 ACG TAT AAA ACC CGT GCA GAA GTG AAA TTT GAG GGC GAT ACC TCT GTG AAT CGC ATT GAA 420
pa-gfp-SL-RC5  361 ACC TAC AAG ACT CGA GCC GAA GTT AAG TTC GAA GGT GAT ACC TCT GTG AAT CGC ATC GAG 420
pa-gfp-SL-RC9  361 ACC TAC AAG ACT CGA GCC GAA GTT AAG TTC GAA GGT GAT ACC TCT GTG AAT CGC ATC GAG 420
pa-gfp-SL-RC15 361 ACC TAC AAG ACT CGA GCC GAA GTT AAG TTC GAA GGA GAT ACC TCT GTG AAT CGC ATA GAG 420

                  Ser Lys Gly Ile Asp Phe Lys Glu Asp Gly Asn Ile Ser Gly His Lys Ser Glu Tyr Asn
pa-gfp-SL      421 TCT AAA GGC ATC GAC TTC AAA GAA GAT GGC AAC ATT TCT GGT CAC AAG TCT GAG TAC AAC 480
pa-gfp-SL-RC5  421 TCT AAA GGC ATC GAT TTC AAG GAA GAT GGG AAC ATC TCT GGT CAT AAA TCT GAG TAT AAC 480
pa-gfp-SL-RC9  421 TCT AAA GGC ATA GAT TTC AAG GAA GAT GGG AAC ATC TCT GGA CAT AAA TCT GAG TAT AAC 480
pa-gfp-SL-RC15 421 TCT AAA GGA ATA GAT TTC AAG GAA GAT GGG AAC ATC TCT GGA CAT AAA TCT GAG TAT AAC 480

                  Phe Asn Leu His Asn Val Tyr Ile Thr Ala Asp Lys Gln Lys Asn Gly Ile Lys Ala Asn
pa-gfp-SL      481 TTT AAC CTT CAT AAC GTG TAC ATT ACT GCC GAT AAA CAG AAG AAC GGT ATC AAA GCC AAC 540
pa-gfp-SL-RC5  481 TTC AAT CTC CAT AAC GTG TAC ATT ACC GCG GAC AAA CAG AAG AAC GGT ATC AAA GCC AAC 540
pa-gfp-SL-RC9  481 TTC AAT CTC CAT AAC GTG TAC ATA ACC GCG GAC AAA CAG AAG AAC GGA ATC AAA GCC AAC 540
pa-gfp-SL-RC15 481 TTC AAT CTC CAT AAC GTG TAC ATA ACC GCG GAC AAA CAG AAG AAC GGA ATA AAA GCC AAC 540

                  Phe Lys Ile Arg His Asn Val Glu Asp Gly Leu Val Gln Ser Ala Asp His Tyr Gln Gln
pa-gfp-SL      541 TTT AAG ATT CGC CAT AAT GTG GAA GAT GGC CTT GTG CAG TCT GCT GAC CAT TAC CAG CAG 600
pa-gfp-SL-RC5  541 TTT AAA ATT CGC CAT AAC GTG GAA GAT GGT CTT GTT CAG TCT GCG GAC CAC TAT CAG CAG 600
pa-gfp-SL-RC9  541 TTT AAA ATT CGC CAT AAC GTG GAA GAT GGT CTT GTT CAG TCT GCG GAC CAC TAT CAG CAG 600
pa-gfp-SL-RC15 541 TTT AAA ATT AGG CAT AAC GTG GAA GAT GGT CTT GTT CAG TCT GCG GAC CAC TAT CAG CAG 600

                  Asn Thr Pro Ile Gly Asp Gly Pro Val Ser Ser Pro Asp Asn His Tyr Ser Leu Thr Gln
pa-gfp-SL      601 AAC ACT CCG ATT GGG GAT GGT CCG GTA TCT TCT CCA GAC AAT CAC TAC TCT CTT ACC CAA 660
pa-gfp-SL-RC5  601 AAT ACC CCT ATA GGC GAT GGA CCA GTT TCC TCT CCC GAT AAC CAC TAC TCT CTT ACC CAA 660
pa-gfp-SL-RC9  601 AAT ACC CCT ATA GGC GAT GGA CCA GTT TCC TCT CCC GAT AAC CAC TAC TCT CTT ACC CAA 660
pa-gfp-SL-RC15 601 AAT ACC CCC ATA GGC GAT GGA CCC GTT TCC TCT CCC GAT AAC CAC TAC TCT CTT ACC CAA 660

                  Leu Val Ser Leu Lys Asp Pro Asn Glu Lys Arg Asp His Met Val Ser Ser Glu Phe Val
pa-gfp-SL      661 CTT GTT TCT CTT AAA GAC CCG AAT GAG AAA CGC GAT CAC ATG GTG TCT TCT GAA TTT GTC 720
pa-gfp-SL-RC5  661 CTC GTA TCT CTT AAA GAC CCG AAT GAG AAA AGG GAT CAT ATG GTG TCT TCC GAG TTT GTG 720
pa-gfp-SL-RC9  661 CTC GTA TCT CTT AAA GAC CCG AAT GAG AAA AGG GAT CAT ATG GTG TCT TCC GAG TTT GTG 720
pa-gfp-SL-RC15 661 CTC GTA TCT CTT AAA GAC CCG AAT GAG AAA AGG GAT CAT ATG GTG TCT TCC GAG TTT GTG 720

                  Thr Ala Ala Gly Ile Thr His Gly Met Asp Glu Ser Tyr Lysstop
pa-gfp-SL      721 ACT GCA GCG GGT ATT ACC CAT GGT ATG GAT GAG TCT TAT AAG TAA                     765
pa-gfp-SL-RC5  721 ACA GCA GCT GGA ATT ACC CAT GGC ATG GAT GAA TCT TAC AAA TAA                     765
pa-gfp-SL-RC9  721 ACA GCA GCT GGA ATT ACC CAT GGA ATG GAT GAA TCT TAC AAA TAA                     765
pa-gfp-SL-RC15 721 ACA GCA GCT GGA ATT ACC CAT GGA ATG GAT GAA TCT TAC AAA TAA                     765
```

pa-gfp-SL

Phe	UUU	7	Ser	UCU	20	Tyr	UAU	5	Cys	UGU	1
	UUC	5		UCC	0		UAC	4		UGC	1
Leu	UUA	0		UCA	0	stop	UAA	1	stop	UGA	0
	UUG	0		UCG	0		UAG	0	Trp	UGG	1
	CUU	10	Pro	CCU	3	His	CAU	6	Arg	CGU	3
	CUC	0		CCC	1		CAC	4		CGC	4
	CUA	0		CCA	2	Gln	CAA	2		CGA	0
	CUG	0		CCG	5		CAG	5		CGG	1
Ile	AUU	6	Thr	ACU	3	Asn	AAU	7	Ser	AGU	0
	AUC	5		ACC	8		AAC	7		AGC	0
	AUA	0		ACA	2	Lys	AAA	17	Arg	AGA	0
Met	AUG	7		ACG	5		AAG	5		AGG	0
Val	GUU	5	Ala	GCU	1	Asp	GAU	13	Gly	GGU	9
	GUC	5		GCC	3		GAC	7		GGC	10
	GUA	3		GCA	2	Glu	GAA	12		GGA	2
	GUG	8		GCG	4		GAG	5		GGG	3

pa-gfp-SL-RC9

Phe	UUU	7	Ser	UCU	18	Tyr	UAU	5	Cys	UGU	1
	UUC	5		UCC	2		UAC	4		UGC	1
Leu	UUA	0		UCA	0	stop	UAA	1	stop	UGA	0
	UUG	0		UCG	0		UAG	0	Trp	UGG	1
	CUU	8	Pro	CCU	3	His	CAU	6	Arg	CGU	1
	CUC	2		CCC	3		CAC	4		CGC	2
	CUA	0		CCA	2	Gln	CAA	2		CGA	1
	CUG	0		CCG	3		CAG	5		CGG	2
Ile	AUU	4	Thr	ACU	3	Asn	AAU	7	Ser	AGU	0
	AUC	4		ACC	8		AAC	7		AGC	0
	AUA	3		ACA	2	Lys	AAA	17	Arg	AGA	1
Met	AUG	7		ACG	5		AAG	5		AGG	1
Val	GUU	5	Ala	GCU	1	Asp	GAU	13	Gly	GGU	7
	GUC	5		GCC	3		GAC	7		GGC	8
	GUA	3		GCA	2	Glu	GAA	12		GGA	6
	GUG	8		GCG	4		GAG	5		GGG	3

pa-gfp-SL-RC5

Phe	UUU	7	Ser	UCU	18	Tyr	UAU	5	Cys	UGU	1
	UUC	5		UCC	2		UAC	4		UGC	1
Leu	UUA	0		UCA	0	stop	UAA	1	stop	UGA	0
	UUG	0		UCG	0		UAG	0	Trp	UGG	1
	CUU	8	Pro	CCU	3	His	CAU	6	Arg	CGU	2
	CUC	2		CCC	1		CAC	4		CGC	2
	CUA	0		CCA	2	Gln	CAA	2		CGA	1
	CUG	0		CCG	5		CAG	5		CGG	1
Ile	AUU	5	Thr	ACU	3	Asn	AAU	7	Ser	AGU	0
	AUC	5		ACC	8		AAC	7		AGC	0
	AUA	1		ACA	2	Lys	AAA	17	Arg	AGA	1
Met	AUG	7		ACG	5		AAG	5		AGG	1
Val	GUU	5	Ala	GCU	1	Asp	GAU	13	Gly	GGU	9
	GUC	5		GCC	3		GAC	7		GGC	10
	GUA	3		GCA	2	Glu	GAA	12		GGA	2
	GUG	8		GCG	4		GAG	5		GGG	3

pa-gfp-SL-RC15

Phe	UUU	7	Ser	UCU	17	Tyr	UAU	5	Cys	UGU	1
	UUC	5		UCC	2		UAC	3		UGC	1
Leu	UUA	0		UCA	0	stop	UAA	0	stop	UGA	0
	UUG	0		UCG	0		UAG	0	Trp	UGG	1
	CUU	8	Pro	CCU	2	His	CAU	5	Arg	CGU	1
	CUC	2		CCC	5		CAC	4		CGC	1
	CUA	0		CCA	1	Gln	CAA	2		CGA	1
	CUG	0		CCG	3		CAG	5		CGG	2
Ile	AUU	2	Thr	ACU	3	Asn	AAU	7	Ser	AGU	0
	AUC	2		ACC	7		AAC	7		AGC	0
	AUA	6		ACA	1	Lys	AAA	16	Arg	AGA	1
Met	AUG	6		ACG	5		AAG	5		AGG	2
Val	GUU	5	Ala	GCU	0	Asp	GAU	12	Gly	GGU	4
	GUC	5		GCC	3		GAC	7		GGC	6
	GUA	3		GCA	1	Glu	GAA	11		GGA	9
	GUG	8		GCG	4		GAG	5		GGG	3

Supplementary Fig. 9. The sequences of *pa-gfp-SL-RC5, 9, 15* genes and their codon usages. Ser and Leu codons are highlighted in green. Rare codons are highlighted in magenta.

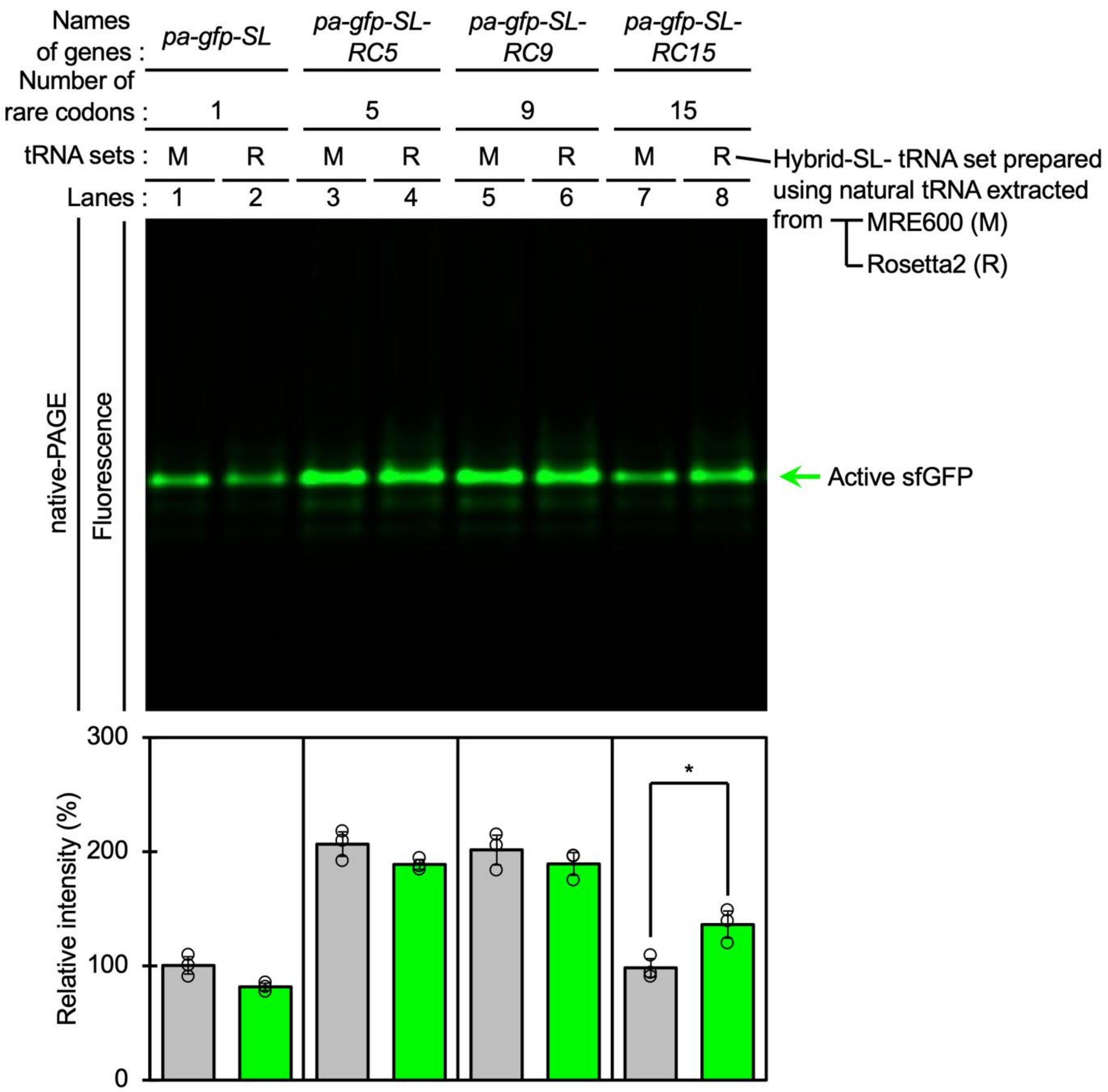

Supplementary Fig. 10. Comparison of hybrid-SL tRNA sets prepared with a ΔSLY natural tRNA set derived from *E. coli* with/without tRNA genes corresponding to rare codons. sfGFP genes containing 1, 5, 9, and 15 rare codons were used. The relative fluorescence band intensities in the native PAGE were normalized against the band intensity in lane 1. Gel image was cropped only for the purpose of this figure. Gel image is representative of n = 3 biologically independent experiments. Bars represent mean ± SD, and open circles represent individual data points for n = 3 biologically independent experiments. Two-tailed Student's T-Test was used for statistical analysis, *$p < 0.05$ ($p = 0.02$). Abbreviations: SL, Ser/Leu-swapped. Source data are provided as a Source Data file.

```
                 Met Asp Pro Ser Lys Asp Ser Lys Ala Gln Val Ser Ala Ala Glu Ala Gly Ile Thr Gly
stv-std      1   ATG GAT CCC TCT AAG GAC TCC AAG GCT CAG GTT TCC GCT GCC GAG GCT GGC ATT ACC GGC  60
stv-SL       1   ATG GAT CCC CTT AAG GAC CTC AAG GCC CAG GTT CTT GCC GCT GAG GCT GGT ATT ACT GGC  60

                 Thr Trp Tyr Asn Gln Leu Gly Ser Thr Phe Ile Val Thr Ala Gly Ala Asp Gly Ala Leu
stv-std     61   ACT TGG TAT AAC CAG CTT GGC TCC ACC TTT ATT GTC ACC GCT GGC GCT GAT GGT GCT CTT 120
stv-SL      61   ACC TGG TAC AAC CAG TCT GGC CTC ACT TTC ATC GTC ACT GCT GGT GCC GAT GGC GCT TCT 120

                 Thr Gly Thr Tyr Glu Ser Ala Val Gly Asn Ala Glu Ser Arg Tyr Val Leu Thr Gly Arg
stv-std    121   ACT GGC ACC TAT GAG TCT GCC GTT GGC AAT GCT GAG TCC CGT TAT GTT CTC ACT GGT CGC 180
stv-SL     121   ACC GGT ACC TAT GAG CTT GCC GTT GGC AAT GCT GAG CTC CGC TAT GTC TCC ACC GGT CGT 180

                 Tyr Asp Ser Ala Pro Ala Thr Asp Gly Ser Gly Thr Ala Leu Gly Trp Thr Val Ala Trp
stv-std    181   TAT GAC TCC GCT CCT GCT ACT GAC GGT TCT GGT ACT GCT CTT GGT TGG ACC GTT GCC TGG 240
stv-SL     181   TAT GAC CTC GCT CCT GCT ACT GAC GGT CTT GGC ACT GCT TCT GGC TGG ACT GTC GCT TGG 240

                 Lys Asn Asn Tyr Arg Asn Ala His Ser Ala Thr Thr Trp Ser Gly Gln Tyr Val Gly Gly
stv-std    241   AAG AAC AAT TAT CGT AAC GCC CAT TCC GCC ACC ACT TGG TCT GGT CAG TAT GTT GGT GGT 300
stv-SL     241   AAG AAC AAC TAC CGC AAT GCC CAT CTT GCC ACC ACT TGG CTT GGT CAG TAC GTT GGT GGT 300

                 Ala Glu Ala Arg Ile Asn Thr Gln Trp Leu Leu Thr Ser Gly Thr Thr Glu Ala Asn Ala
stv-std    301   GCT GAG GCT CGT ATC AAT ACT CAG TGG CTT CTC ACC TCC GGC ACT ACC GAG GCT AAC GCC 360
stv-SL     301   GCT GAG GCC CGT ATC AAC ACC CAG TGG TCC TCC ACT CTC GGC ACC ACT GAG GCC AAT GCC 360

                 Trp Lys Ser Thr Leu Val Gly His Asp Thr Phe Thr Lys Val Lys Pro Ser Ala Ala Ser
stv-std    361   TGG AAG TCT ACC CTT GTT GGT CAC GAC ACT TTC ACT AAG GTC AAG CCC TCC GCC GCC TCT 420
stv-SL     361   TGG AAG CTC ACT TCC GTT GGT CAC GAC ACC TTT ACC AAG GTC AAG CCT CTT GCT GCC CTC 420

                 Ile Asp Ala Ala Lys Lys Ala Gly Val Asn Asn Gly Asn Pro Leu Asp Ala Val Gln Gln
stv-std    421   ATC GAT GCT GCT AAG AAG GCT GGC GTC AAT AAC GGT AAC CCT CTT GAT GCC GTC CAG CAG 480
stv-SL     421   ATT GAT GCT GCC AAG AAG GCT GGC GTC AAC AAT GGC AAT CCC TCT GAT GCC GTT CAG CAG 480

                stop
stv-std    481   TAA                                                                             483
stv-SL     481   TAA                                                                             483
```

stv-std

Phe	UUU	1	Ser	UCU	6	Tyr	UAU	6	Cys	UGU	0
	UUC	1		UCC	8		UAC	0		UGC	0
Leu	UUA	0		UCA	0	stop	UAA	1	stop	UGA	0
	UUG	0		UCG	0		UAG	0	Trp	UGG	6
	CUU	6	Pro	CCU	2	His	CAU	1	Arg	CGU	3
	CUC	2		CCC	2		CAC	1		CGC	1
	CUA	0		CCA	0	Gln	CAA	0		CGA	0
	CUG	0		CCG	0		CAG	6		CGG	0
Ile	AUU	2	Thr	ACU	10	Asn	AAU	4	Ser	AGU	0
	AUC	2		ACC	9		AAC	6		AGC	0
	AUA	0		ACA	0	Lys	AAA	0	Arg	AGA	0
Met	AUG	1		ACG	0		AAG	8		AGG	0
Val	GUU	6	Ala	GCU	16	Asp	GAU	4	Gly	GGU	10
	GUC	4		GCC	9		GAC	4		GGC	8
	GUA	0		GCA	0	Glu	GAA	0		GGA	0
	GUG	0		GCG	0		GAG	5		GGG	0

stv-SL

Phe	UUU	1	Ser	UCU	4	Tyr	UAU	3	Cys	UGU	0
	UUC	1		UCC	4		UAC	3		UGC	0
Leu	UUA	0		UCA	0	stop	UAA	1	stop	UGA	0
	UUG	0		UCG	0		UAG	0	Trp	UGG	6
	CUU	7	Pro	CCU	2	His	CAU	1	Arg	CGU	2
	CUC	7		CCC	2		CAC	1		CGC	2
	CUA	0		CCA	0	Gln	CAA	0		CGA	0
	CUG	0		CCG	0		CAG	6		CGG	0
Ile	AUU	2	Thr	ACU	10	Asn	AAU	5	Ser	AGU	0
	AUC	2		ACC	9		AAC	5		AGC	0
	AUA	0		ACA	0	Lys	AAA	0	Arg	AGA	0
Met	AUG	1		ACG	0		AAG	8		AGG	0
Val	GUU	5	Ala	GCU	13	Asp	GAU	4	Gly	GGU	9
	GUC	5		GCC	12		GAC	4		GGC	9
	GUA	0		GCA	0	Glu	GAA	0		GGA	0
	GUG	0		GCG	0		GAG	5		GGG	0

Supplementary Fig. 11. The sequences of *stv-Std, stv-SL* genes used for PAGE analysis shown in Supplementary Fig. 12 and their codon usages. Ser and Leu codons are highlighted in green.

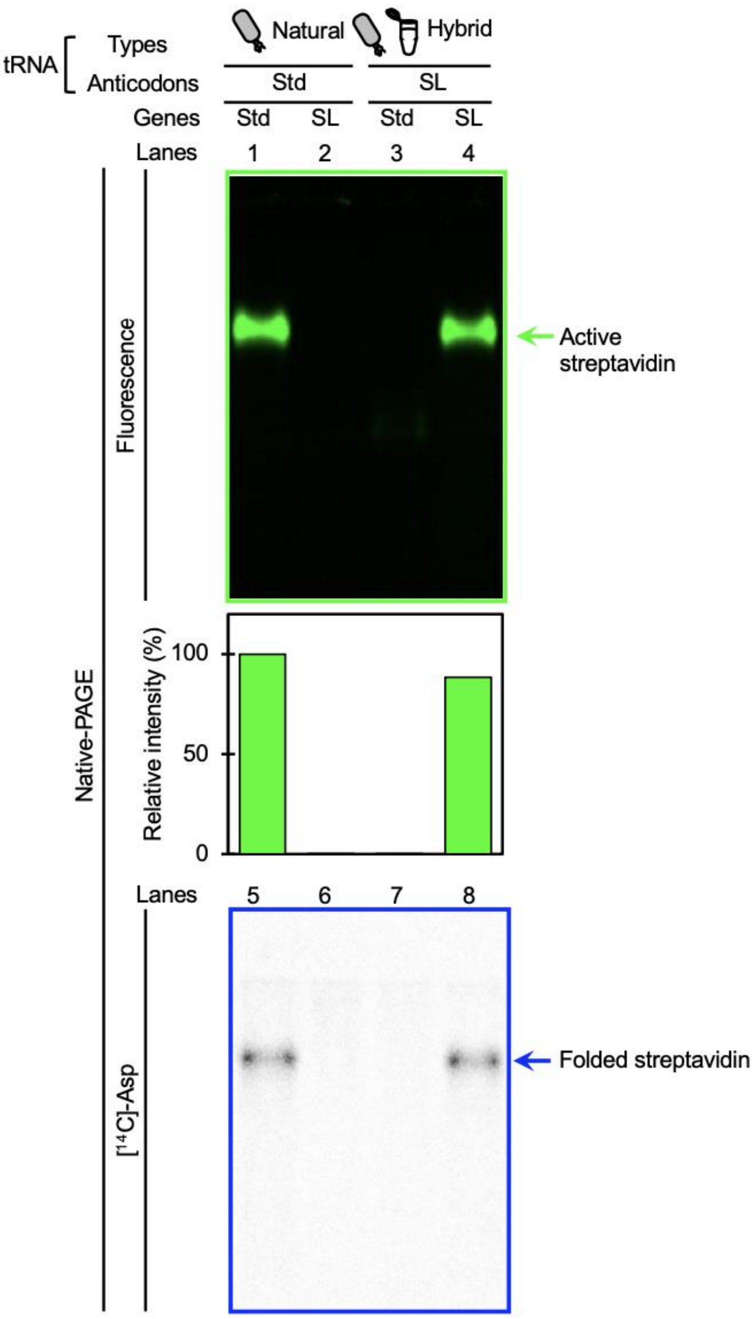

Supplementary Fig. 12. Comparison of the natural tRNA extract and the hybrid-SL tRNA set for the translation of the streptavidin coded with the standard genetic code (stv-Std gene) or the Ser/Leu-swapped genetic code (stv-SL gene). Active streptavidin was labeled using Atto 488-biotin added to the translation mixture. The upper panel shows the result of native PAGE followed by fluorescence imaging (n = 1). The bottom panel displays the autoradiography result of the native PAGE. Gel images were cropped only for the purpose of this figure. The relative band intensities were normalized against the intensity observed with the combination of the natural tRNA extract and the *stv-Std* gene (lane 1). Abbreviations: Std, standard; SL, Ser/Leu-swapped. Source data are provided as a Source Data file.

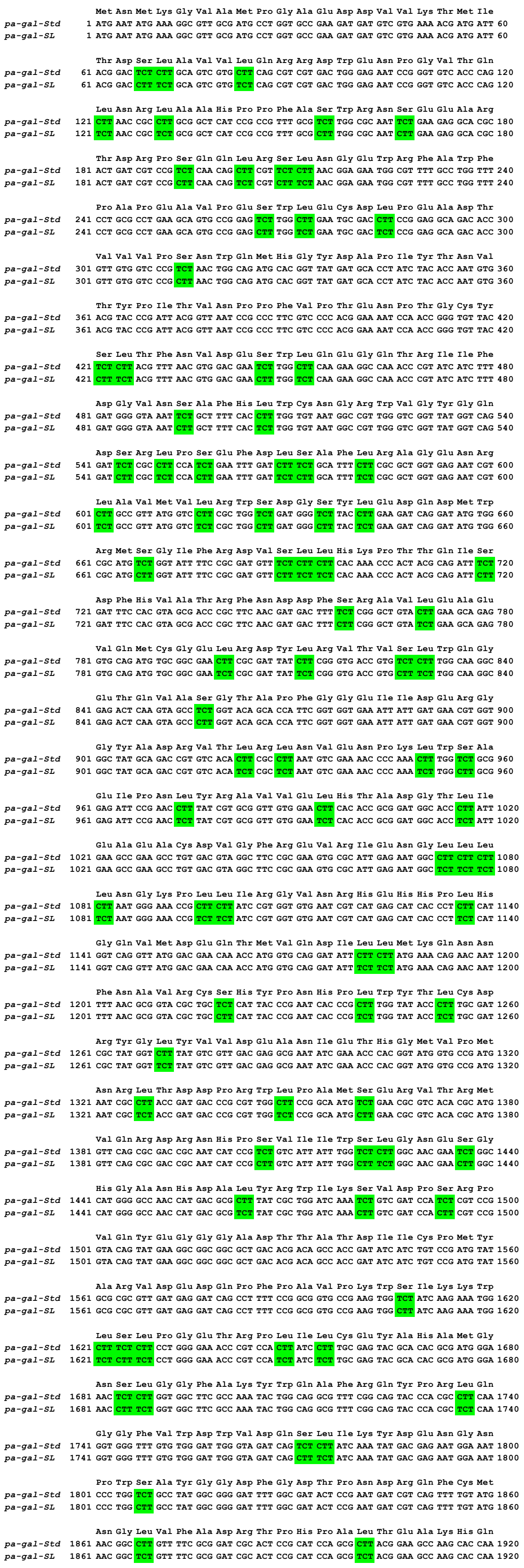

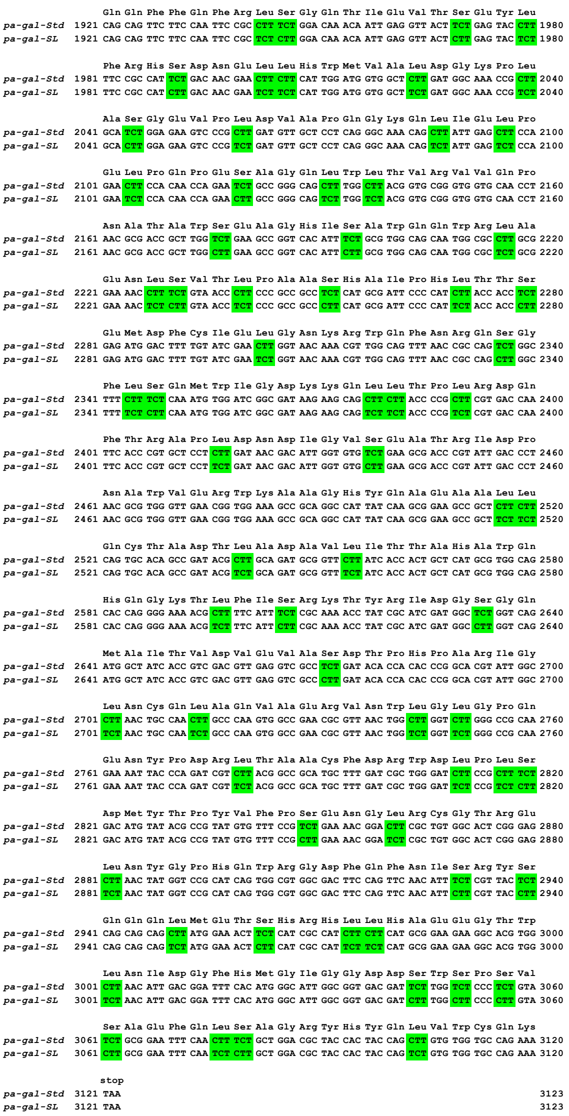

pa-gal-Std

Phe	UUU	22	Ser	UCU	60	Tyr	UAU	18	Cys	UGU	7
	UUC	16		UCC	0		UAC	13		UGC	9
Leu	UUA	0		UCA	0	stop	UAA	1	stop	UGA	0
	UUG	0		UCG	0		UAG	0	Trp	UGG	39
	CUU	96	Pro	CCU	11	His	CAU	19	Arg	CGU	25
	CUC	0		CCC	8		CAC	15		CGC	35
	CUA	0		CCA	12	Gln	CAA	20		CGA	0
	CUG	0		CCG	32		CAG	38		CGG	6
Ile	AUU	23	Thr	ACU	9	Asn	AAU	22	Ser	AGU	0
	AUC	16		ACC	25		AAC	26		AGC	0
	AUA	0		ACA	7	Lys	AAA	17	Arg	AGA	0
Met	AUG	26		ACG	15		AAG	5		AGG	0
Val	GUU	17	Ala	GCU	13	Asp	GAU	41	Gly	GGU	24
	GUC	16		GCC	22		GAC	25		GGC	30
	GUA	10		GCA	16	Glu	GAA	43		GGA	8
	GUG	24		GCG	28		GAG	20		GGG	11

pa-gal-SL

Phe	UUU	22	Ser	UCU	96	Tyr	UAU	18	Cys	UGU	7
	UUC	16		UCC	0		UAC	13		UGC	9
Leu	UUA	0		UCA	0	stop	UAA	1	stop	UGA	0
	UUG	0		UCG	0		UAG	0	Trp	UGG	39
	CUU	60	Pro	CCU	11	His	CAU	19	Arg	CGU	25
	CUC	0		CCC	8		CAC	15		CGC	35
	CUA	0		CCA	12	Gln	CAA	20		CGA	0
	CUG	0		CCG	32		CAG	38		CGG	6
Ile	AUU	23	Thr	ACU	9	Asn	AAU	22	Ser	AGU	0
	AUC	16		ACC	25		AAC	26		AGC	0
	AUA	0		ACA	7	Lys	AAA	17	Arg	AGA	0
Met	AUG	26		ACG	15		AAG	5		AGG	0
Val	GUU	17	Ala	GCU	13	Asp	GAU	41	Gly	GGU	24
	GUC	16		GCC	22		GAC	25		GGC	30
	GUA	10		GCA	16	Glu	GAA	43		GGA	8
	GUG	24		GCG	28		GAG	20		GGG	11

Supplementary Fig. 13. The sequences of *pa-gal-Std, pa-gal-SL* and their codon usages.
Ser and Leu codons are highlighted in green.

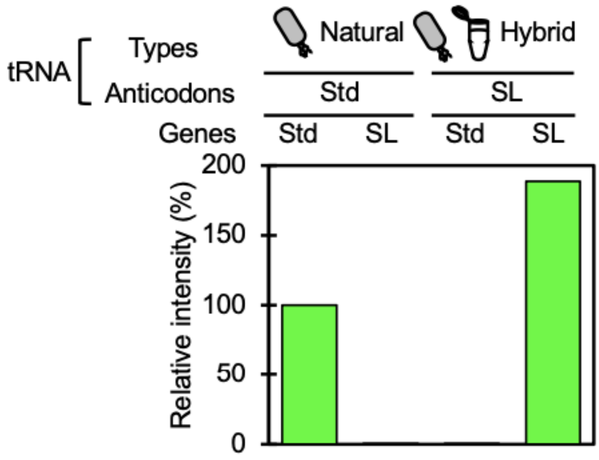

Supplementary Fig. 14. Comparison of natural tRNA extract and the hybrid-SL tRNA set for the translation of β-galactosidase encoded by the standard genetic code (*pa-gal-Std* gene) and the Ser/Leu-swapped genetic code (*pa-gal-SL* gene). 50 µM Fluorescein di-β-D-galactopyranoside was added to the translation reaction mixture. After incubation at 37˚C for 30 min, the solution was diluted 40 times with 25 mM Hepes-K pH7.5, 150 mM NaCl and analyzed using fluorescence spectrometry (n = 1). The relative intensities were normalized against the intensity of the combination of the natural tRNA extract and the p*a-gal-Std gene*. Abbreviations: Std, standard; SL, Ser/Leu-swapped. Source data are provided as a Source Data file.

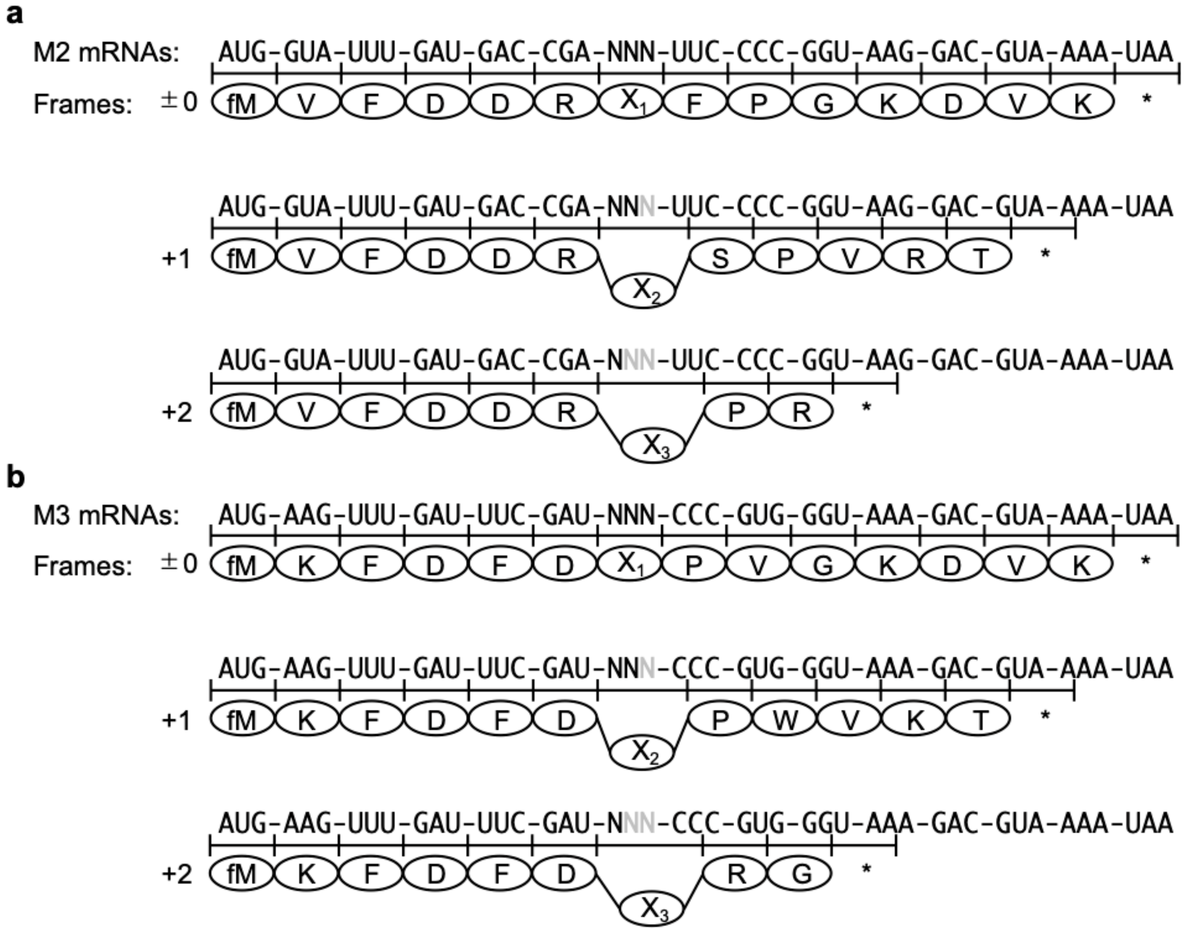

Supplementary Fig. 15. M2 and M3 mRNAs to study frameshifts. a Sequence of M2 mRNA and possible frameshift products. **b** Sequence of M3 mRNA and possible frameshift products. The frameshifts will result in termination at different positions.

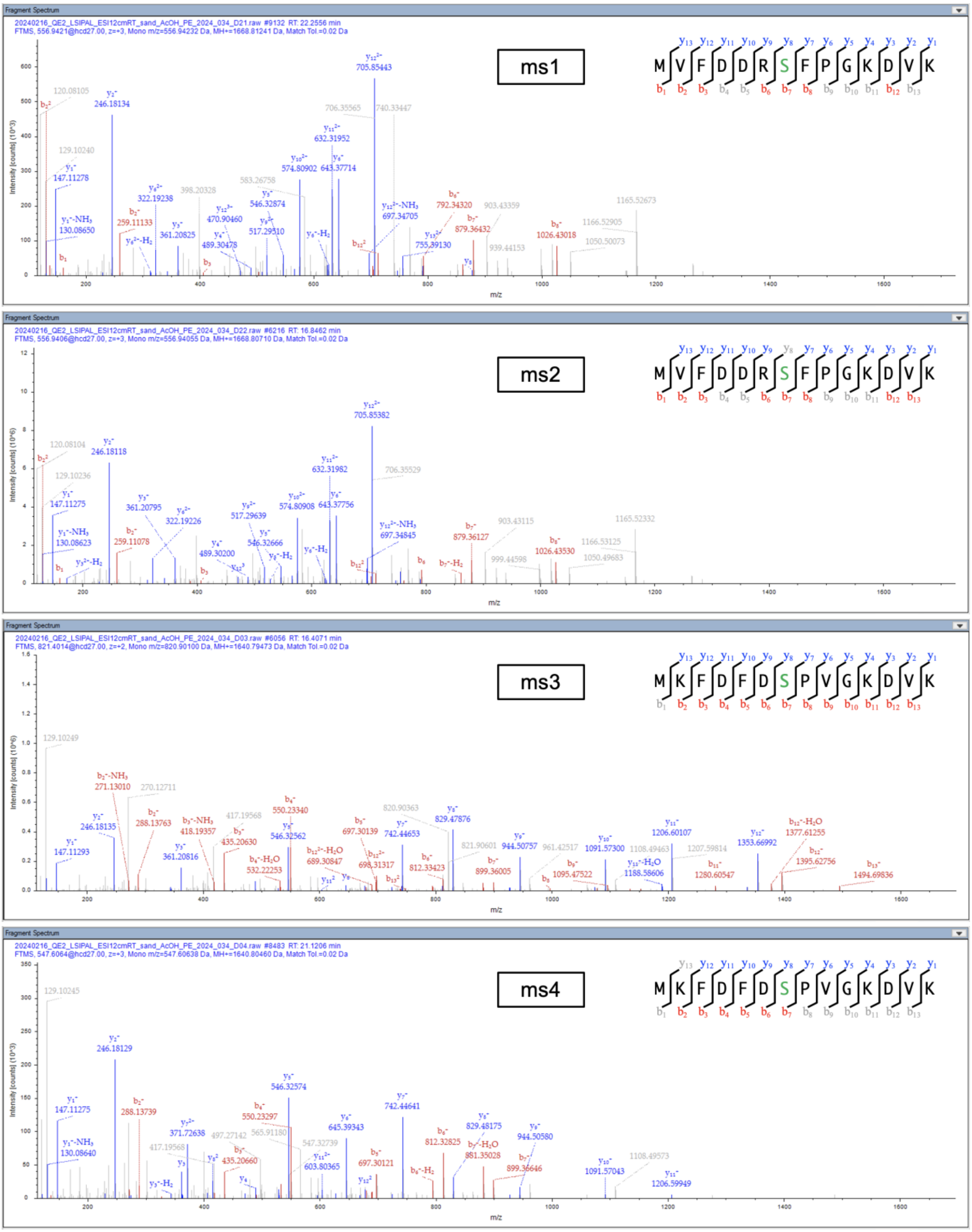

Supplementary Fig. 16. MS/MS data of peptides in Fig. 5 and Supplementary Table 3 and 4 (ms1–ms4). The peaks of the y ions are represented in blue, and the b ions in red. Identified y and b ions are highlighted in the panel at upper right. Amino acids incorporated into CU(U/C) or UC(U/C) codons are represented in green.

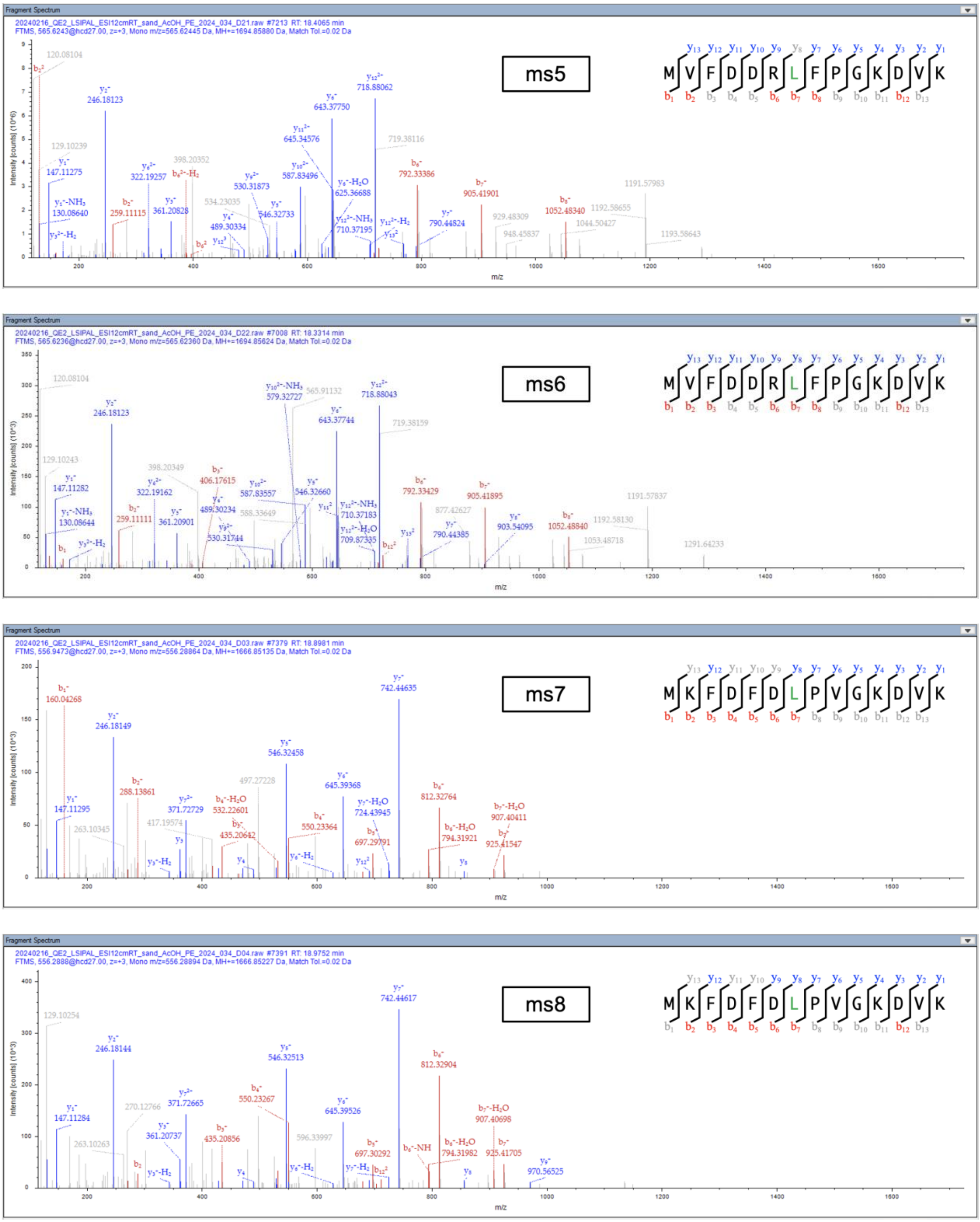

Supplementary Fig. 17. MS/MS data of peptides in Fig. 5 and Supplementary Table 3 and 4 (ms5–ms8). The peaks of the y ions are represented in blue, and the b ions in red. Identified y and b ions are highlighted in the panel at upper right. Amino acids incorporated into CU(U/C) or UC(U/C) codons are represented in green.

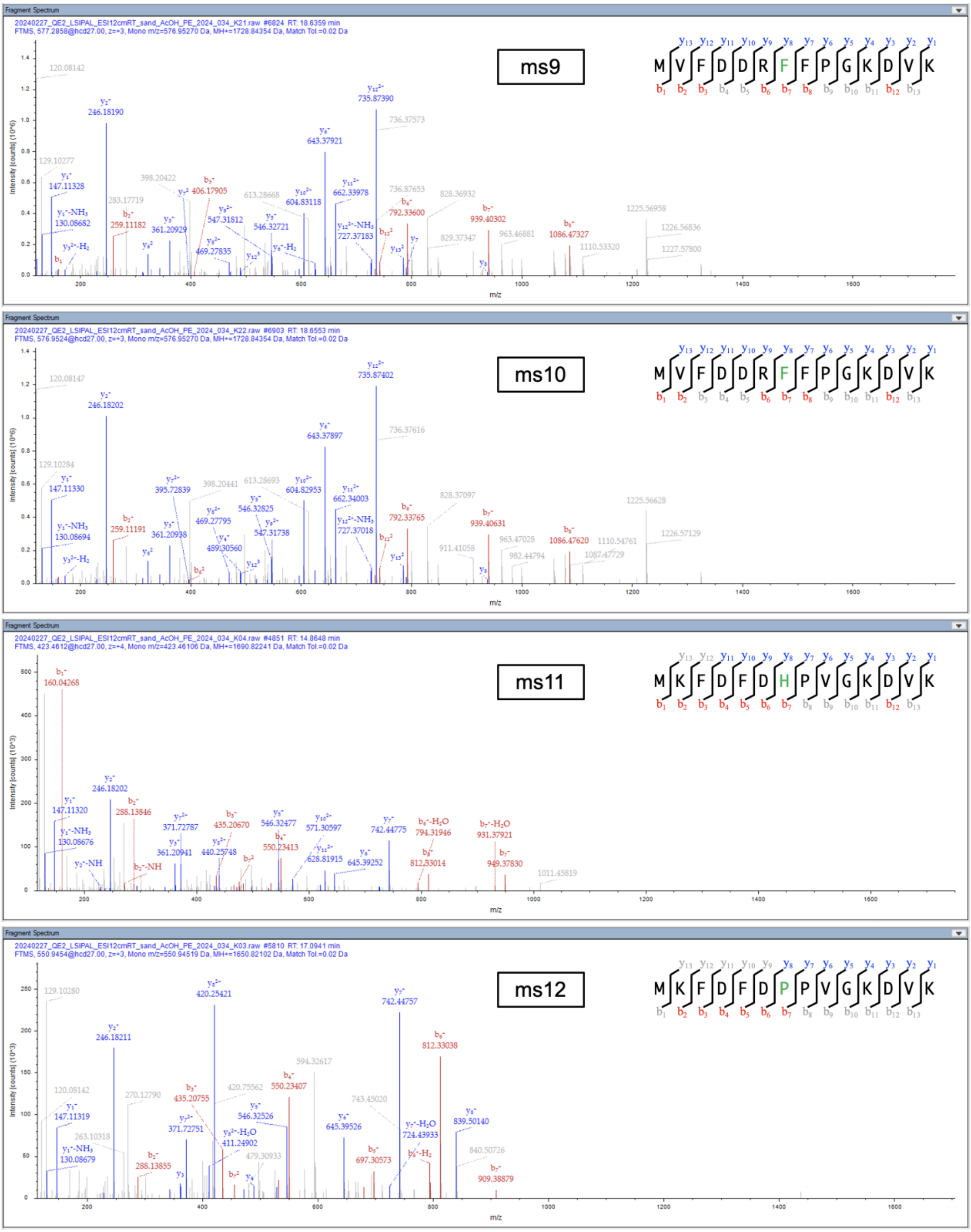

Supplementary Fig. 18. MS/MS data of peptides in Fig. 5 and Supplementary Table 3 and 4 (ms9–ms12). The peaks of the y ions are represented in blue, and the b ions in red. Identified y and b ions are highlighted in the panel at upper right. Amino acids incorporated into CU(U/C) or UC(U/C) codons are represented in green.

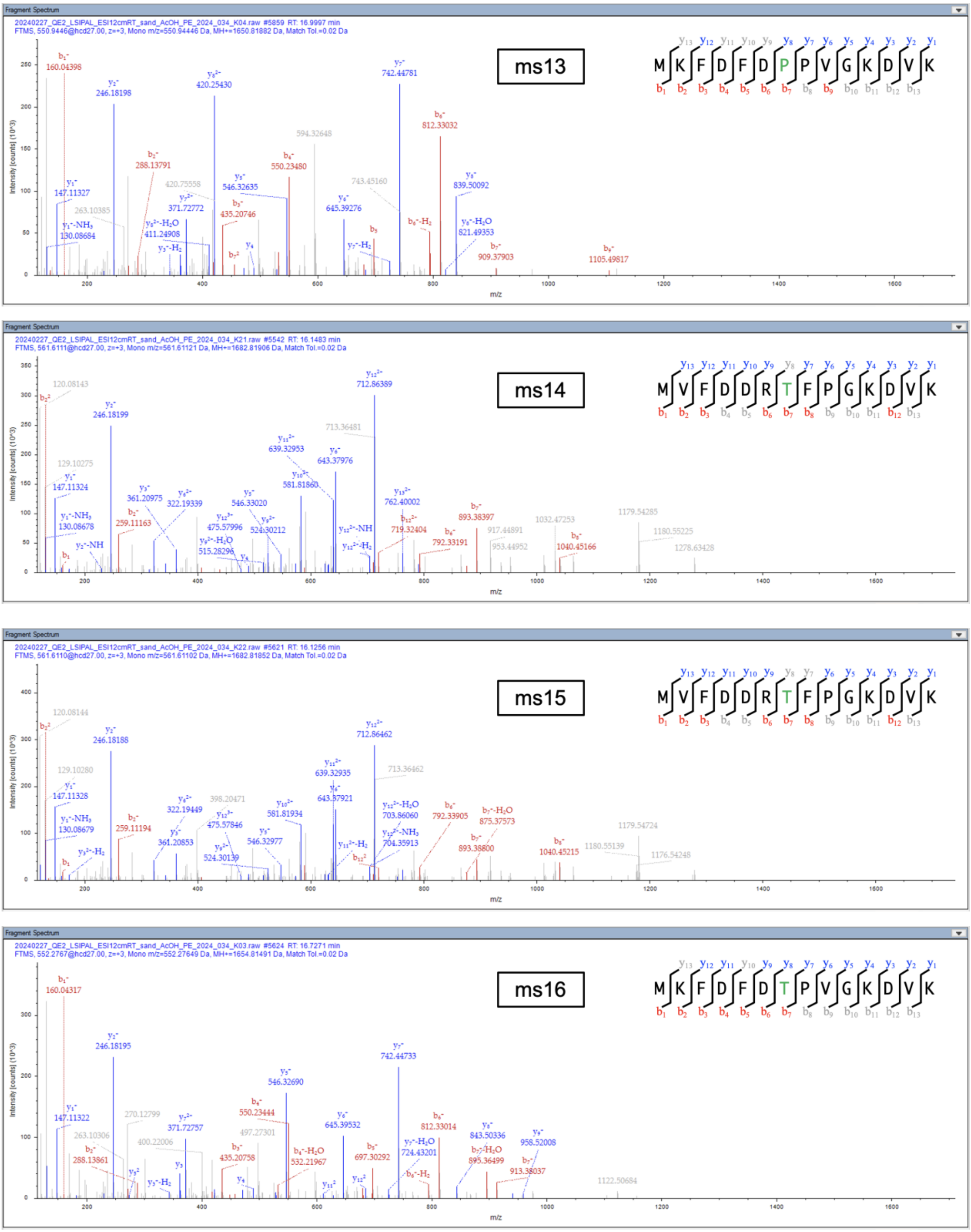

Supplementary Fig. 19. MS/MS data of peptides in Fig. 5 and Supplementary Table 3 and 4 (ms13–ms16). The peaks of the y ions are represented in blue, and the b ions in red. Identified y and b ions are highlighted in the panel at upper right. Amino acids incorporated into CU(U/C) or UC(U/C) codons are represented in green.

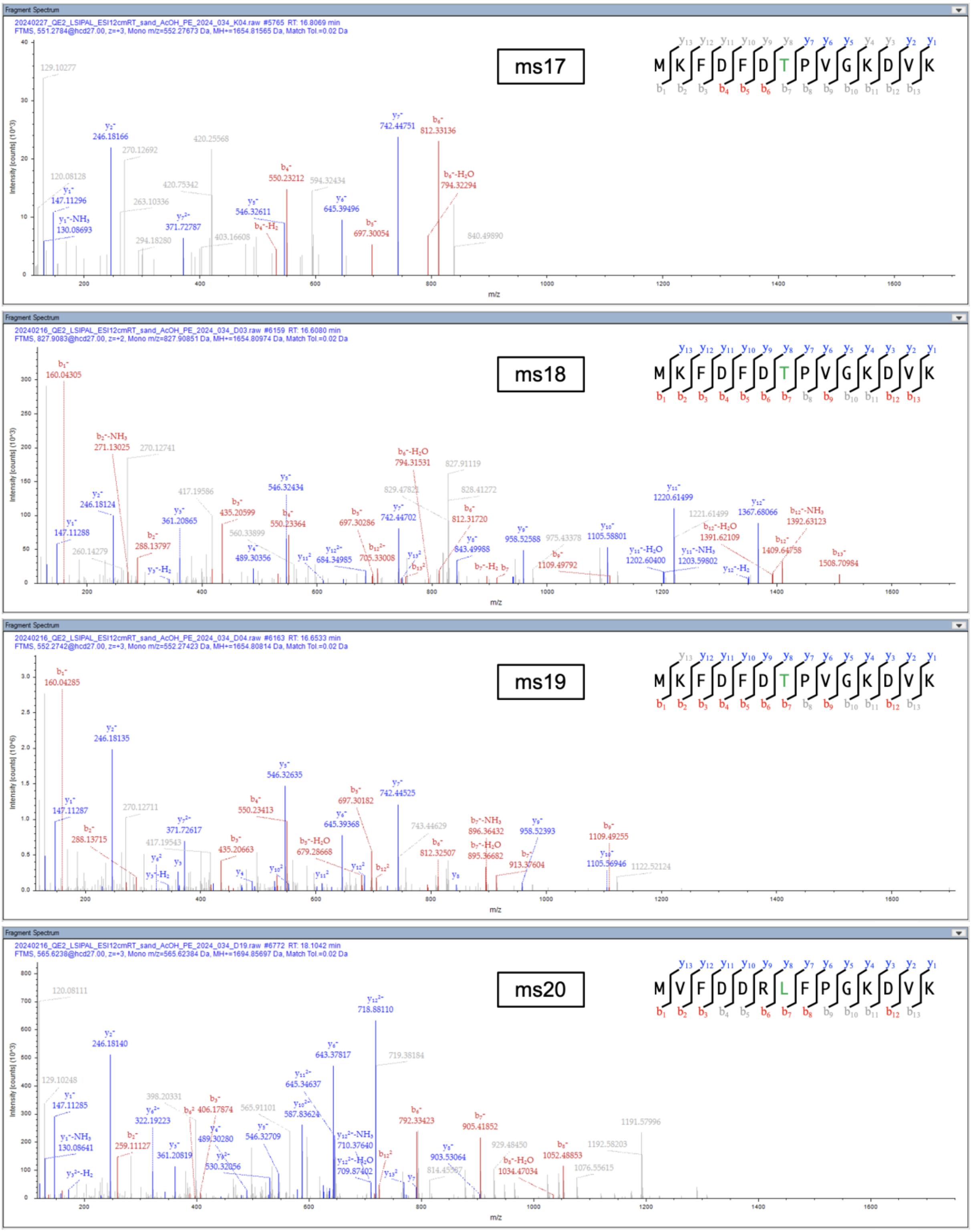

Supplementary Fig. 20. MS/MS data of peptides in Fig. 5 and Supplementary Table 3 and 4 (ms17–ms20). The peaks of the y ions are represented in blue, and the b ions in red. Identified y and b ions are highlighted in the panel at upper right. Amino acids incorporated into CU(U/C) or UC(U/C) codons are represented in green.

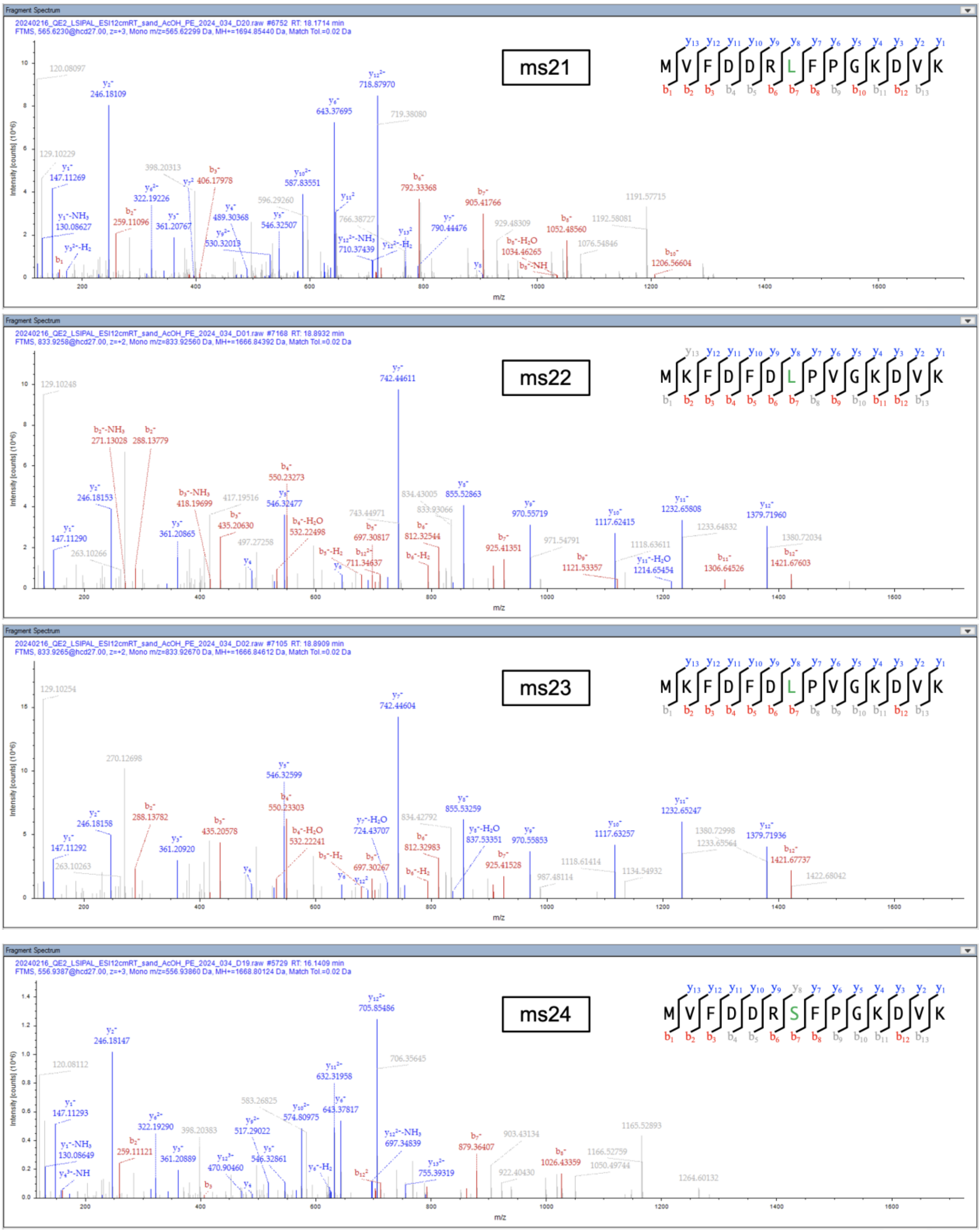

Supplementary Fig. 21. MS/MS data of peptides in Fig. 5 and Supplementary Table 3 and 4 (ms21–ms24). The peaks of the y ions are represented in blue, and the b ions in red. Identified y and b ions are highlighted in the panel at upper right. Amino acids incorporated into CU(U/C) or UC(U/C) codons are represented in green.

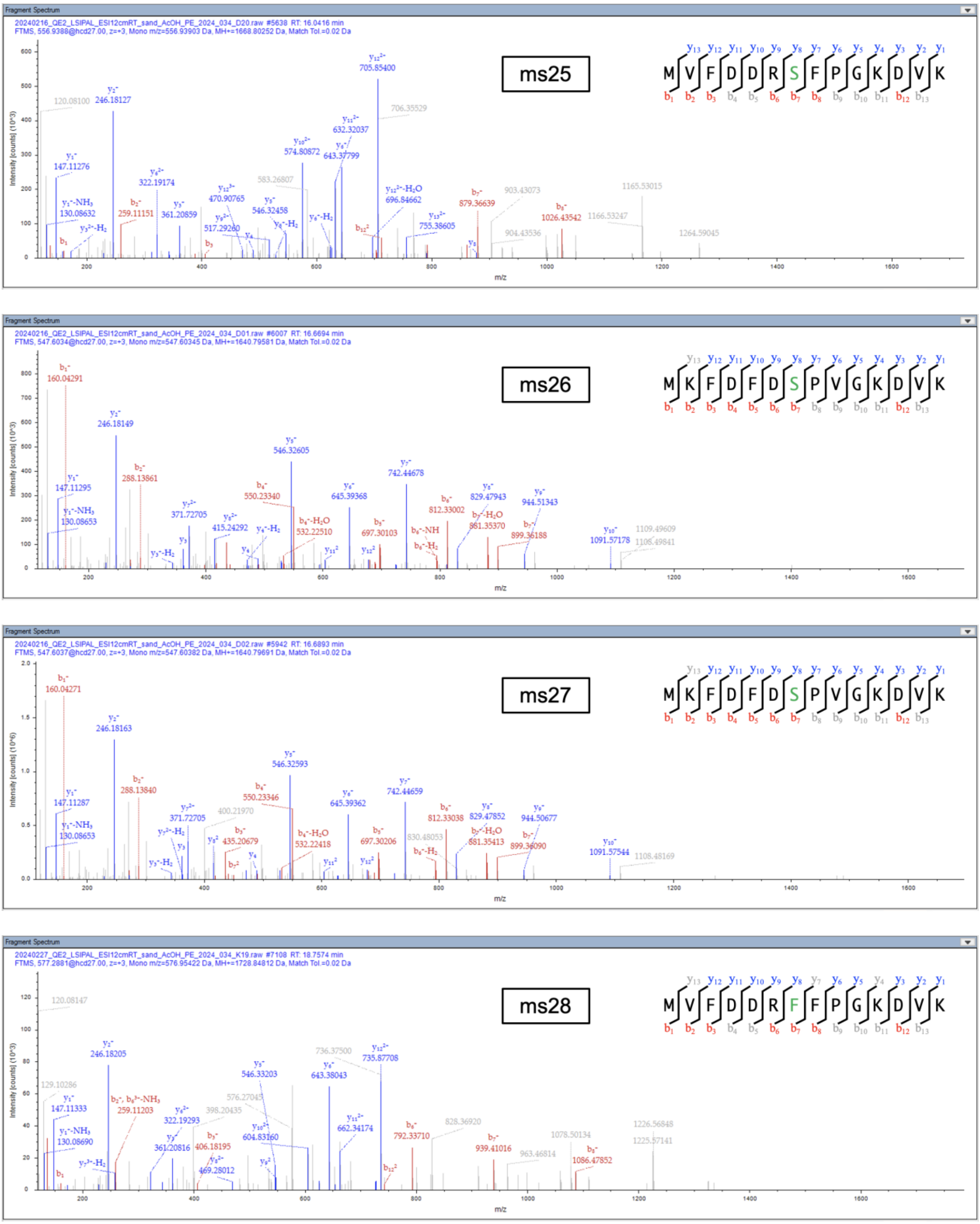

Supplementary Fig. 22. MS/MS data of peptides in Fig. 5 and Supplementary Table 3 and 4 (ms25–ms28). The peaks of the y ions are represented in blue, and the b ions in red. Identified y and b ions are highlighted in the panel at upper right. Amino acids incorporated into CU(U/C) or UC(U/C) codons are represented in green.

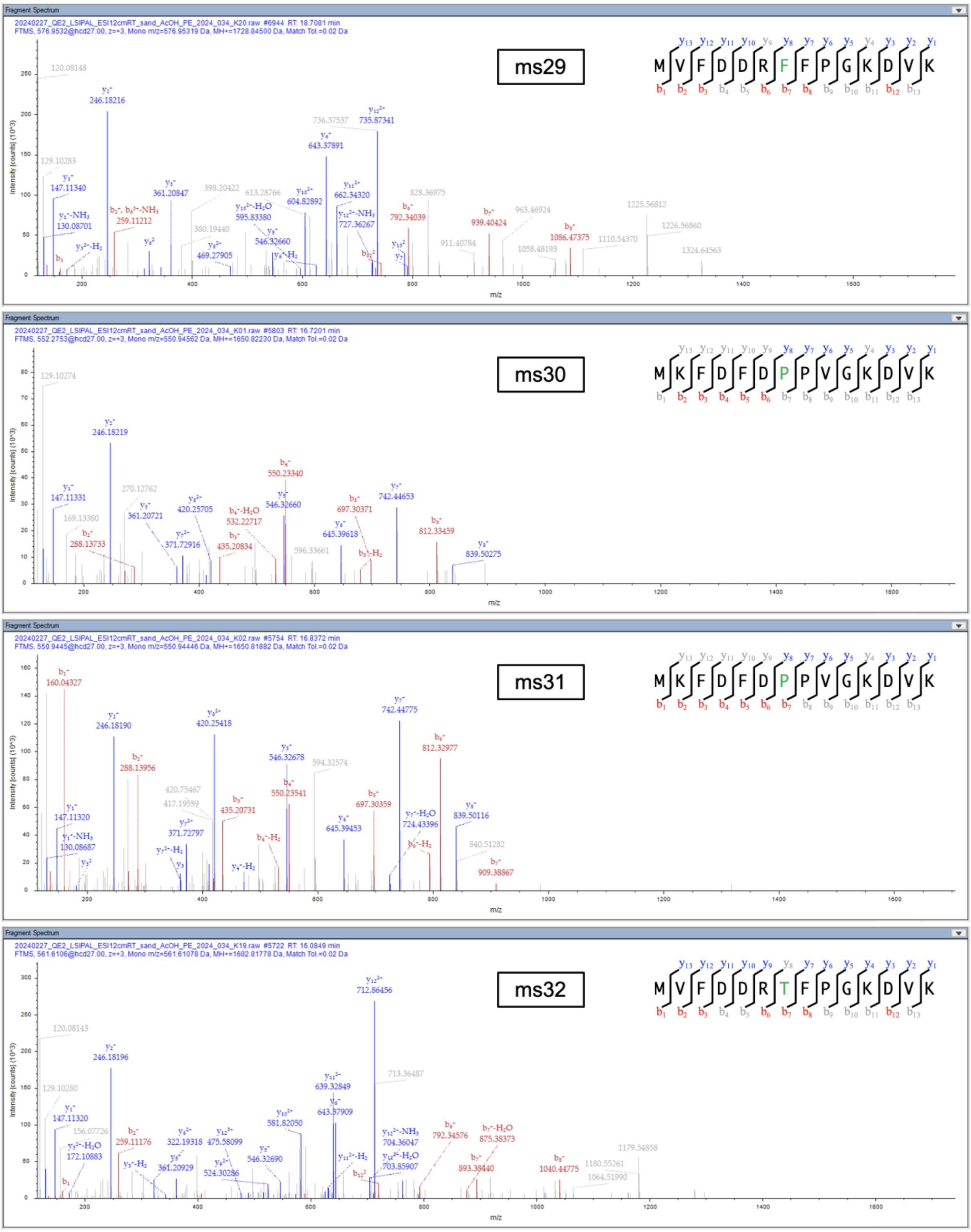

Supplementary Fig. 23. MS/MS data of peptides in Fig. 5 and Supplementary Table 3 and 4 (ms29–ms32). The peaks of the y ions are represented in blue, and the b ions in red. Identified y and b ions are highlighted in the panel at upper right. Amino acids incorporated into CU(U/C) or UC(U/C) codons are represented in green.

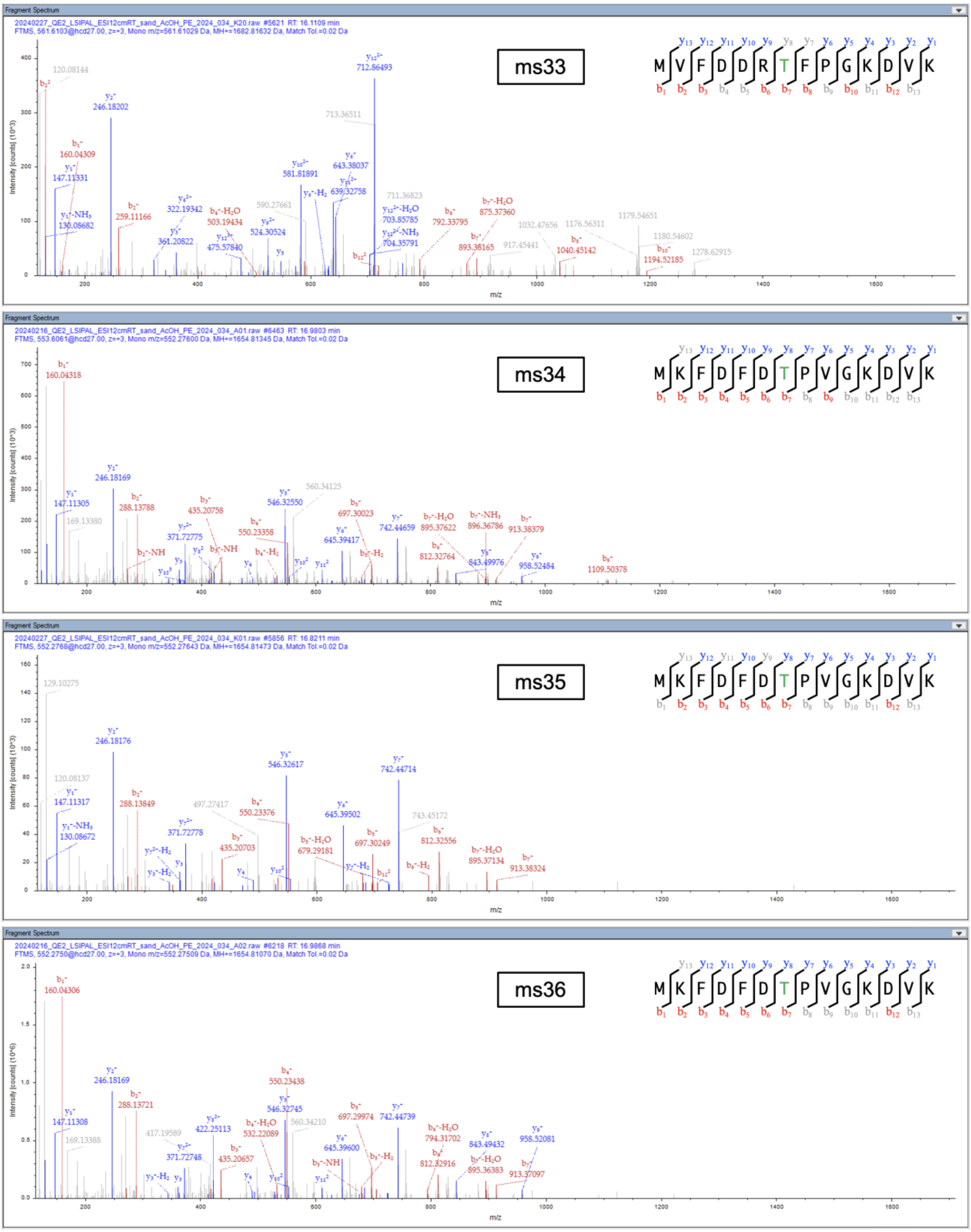

Supplementary Fig. 24. MS/MS data of peptides in Fig. 5 and Supplementary Table 3 and 4 (ms33–ms36). The peaks of the y ions are represented in blue, and the b ions in red. Identified y and b ions are highlighted in the panel at upper right. Amino acids incorporated into CU(U/C) or UC(U/C) codons are represented in green.

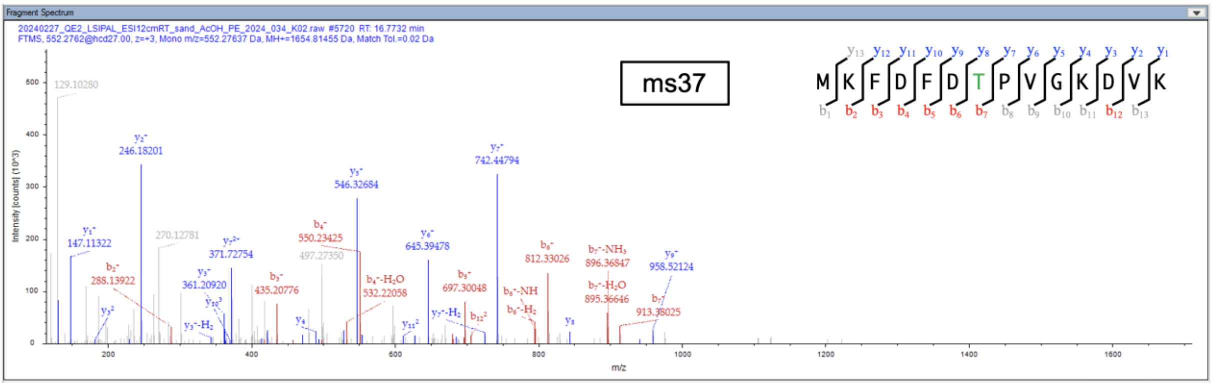

Supplementary Fig. 25. MS/MS data of peptide in Fig. 5 and Supplementary Table 3 and 4 (ms37). The peaks of the y ions are represented in blue, and the b ions in red. Identified y and b ions are highlighted in the panel at upper right. Amino acids incorporated into CU(U/C) or UC(U/C) codons are represented in green.

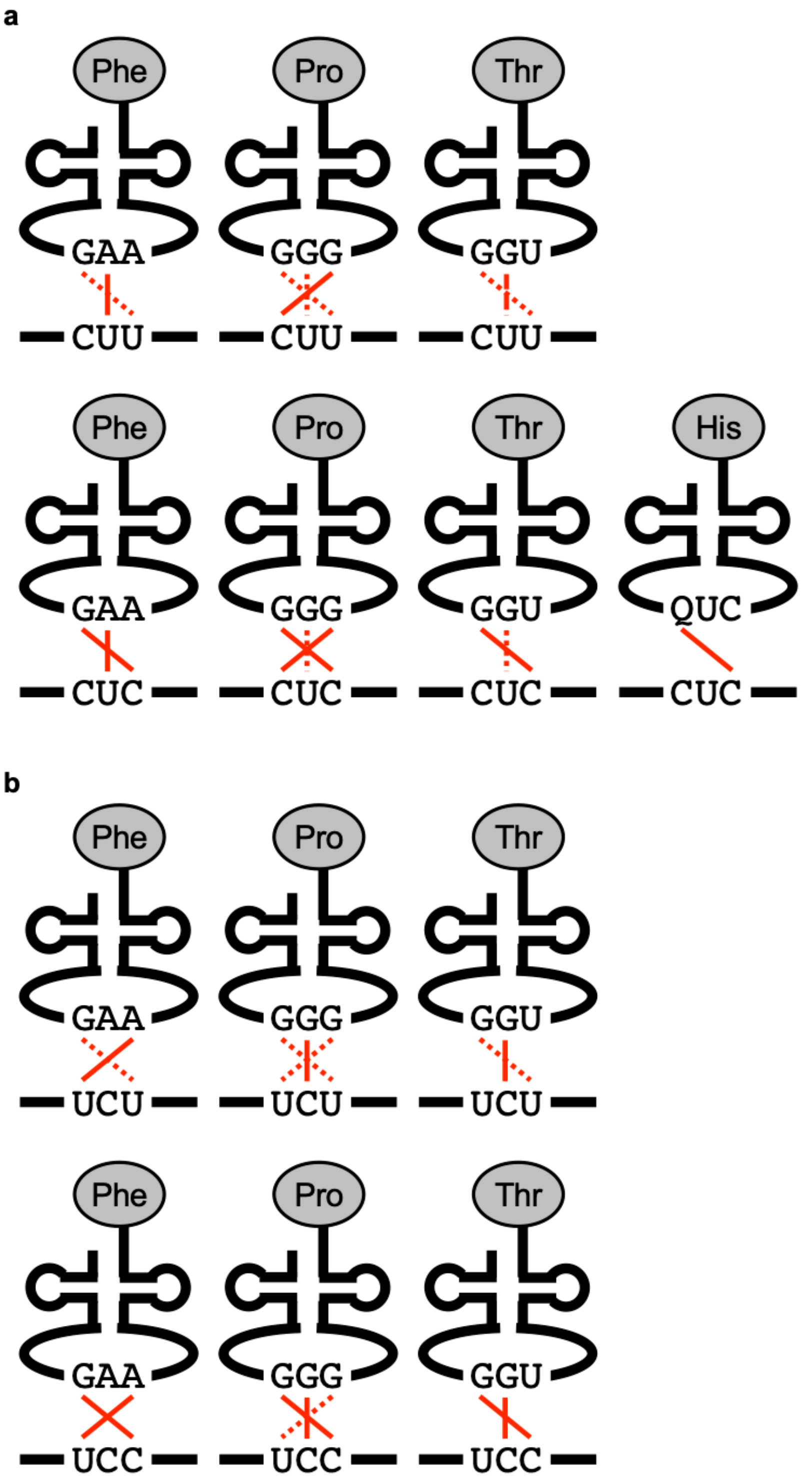

Supplementary Fig. 26. Possible codon-anticodon pairs that induce misincorporation at CUU/C codons and UCU/C codons. a Possible codon-anticodon pairs at CUU/C codons. **b** Possible codon-anticodon pairs at UCU/C codons. Watson-Crick base pairs are indicated by solid red lines, while G-U wobble base pairs are indicated by dotted red lines.

Names of the frameshift products shown in Fig. 6	MS/MS spectrum No.
f1	ms38
f2	ms39
f3	ms40
f4	ms41
f5	ms42
f6	ms43
f7	ms44
f8	ms45
f9	ms46
f10	ms47
f11	ms48
f12	ms49

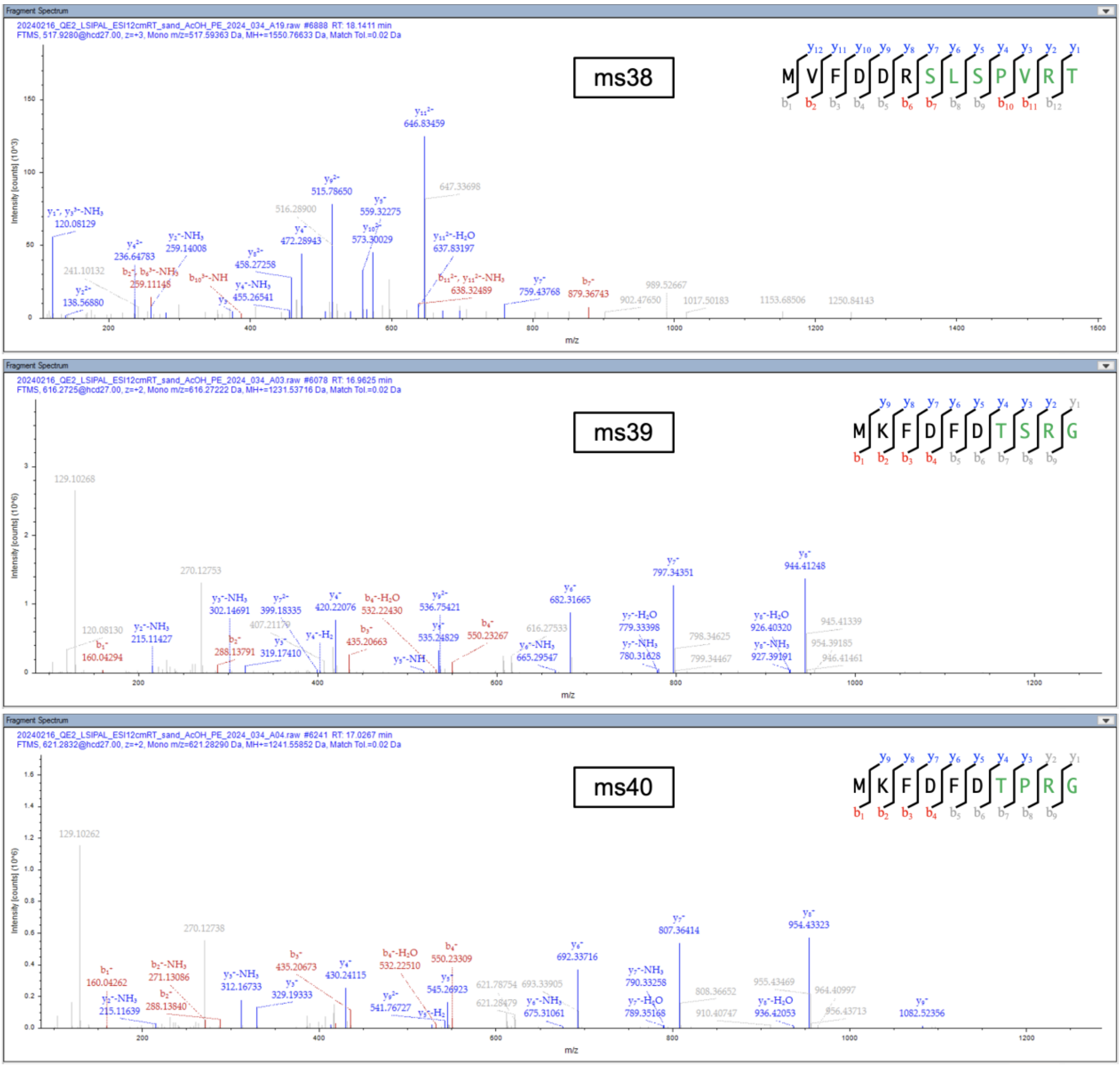

Supplementary Fig. 27. MS/MS data of peptides in Fig. 6 (ms38–ms40). The peaks of the y ions are represented in blue, and the b ions in red. Identified y and b ions are highlighted in the panel at upper right. Amino acid sequences produced by frameshifts are represented in green.

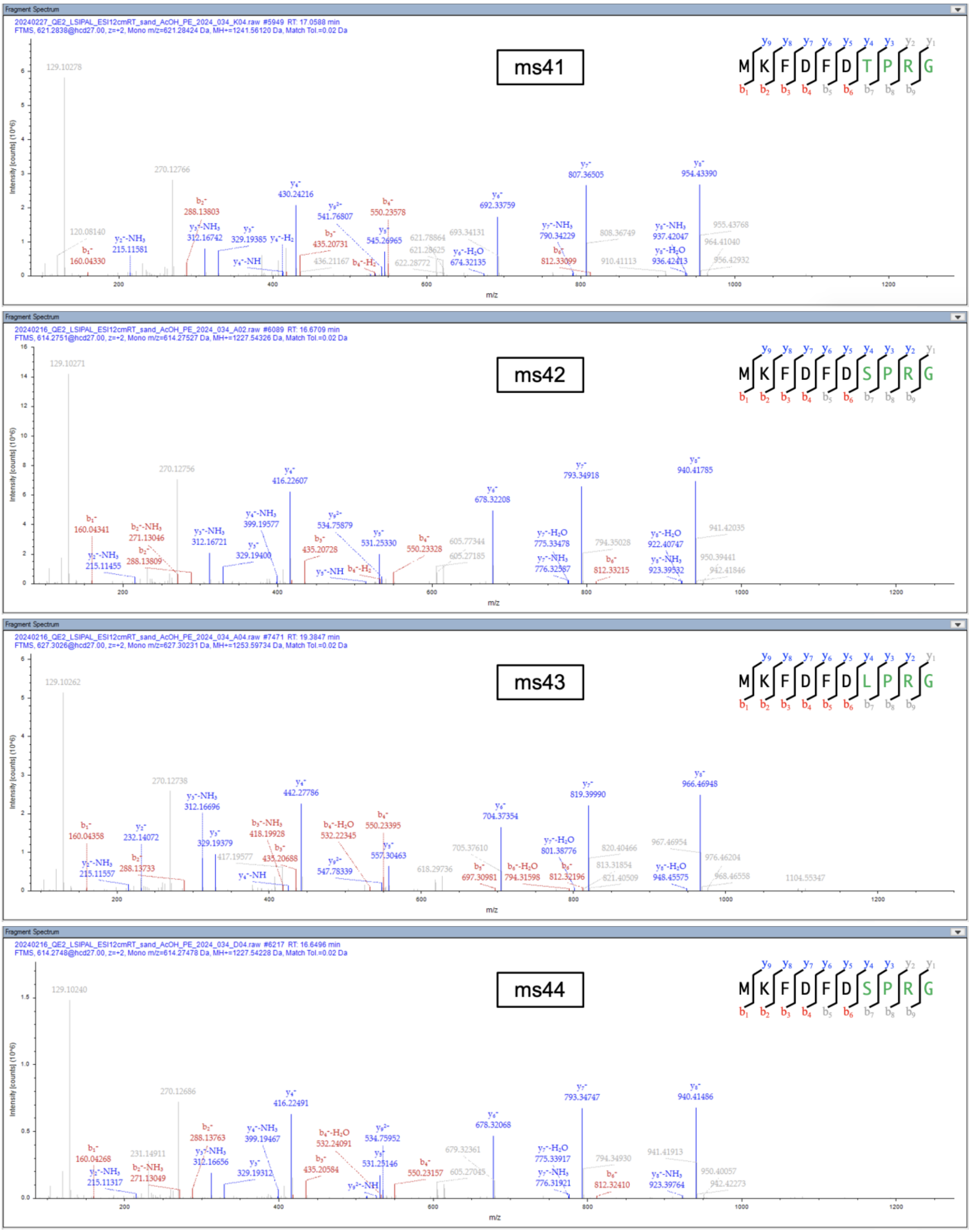

Supplementary Fig. 28. MS/MS data of peptides in Fig. 6 (ms41–ms44, Supplementary Fig. 27). The peaks of the y ions are represented in blue, and the b ions in red. Identified y and b ions are highlighted in the panel at upper right. Amino acid sequences produced by frameshifts are represented in green.

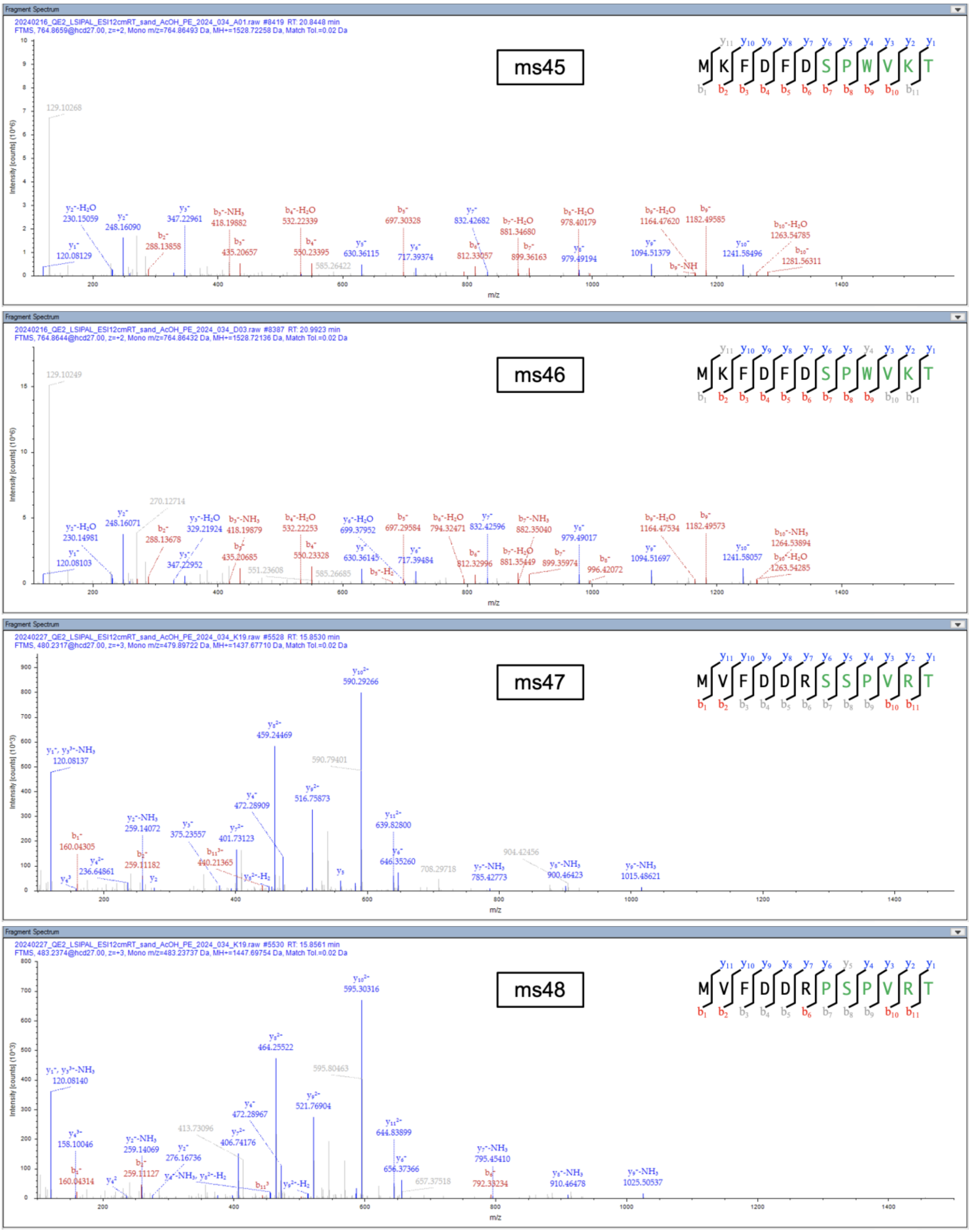

Supplementary Fig. 29. MS/MS data of peptides in Fig. 6 (ms45–ms48, Supplementary Fig. 27). The peaks of the y ions are represented in blue, and the b ions in red. Identified y and b ions are highlighted in the panel at upper right. Amino acid sequences produced by frameshifts are represented in green.

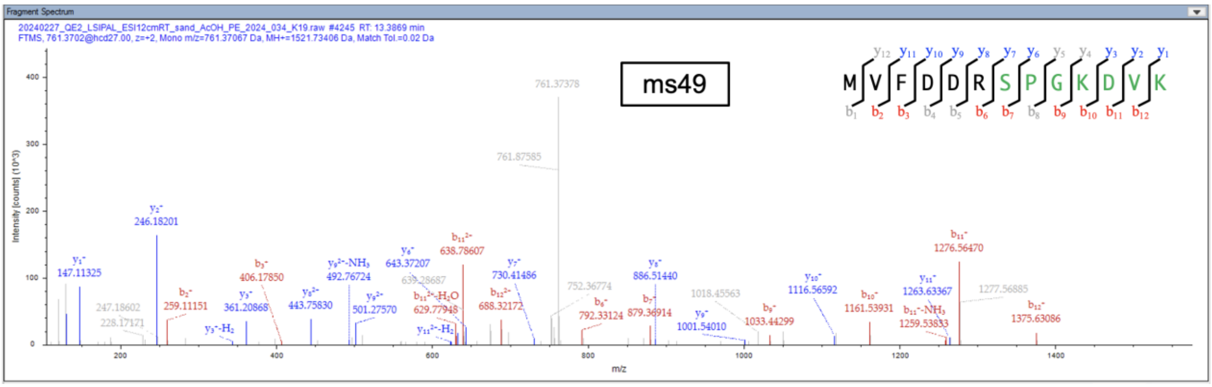

Supplementary Fig. 30. MS/MS data of peptide in Fig. 6 (ms49, Supplementary Fig. 27). The peaks of the y ions are represented in blue, and the b ions in red. Identified y and b ions are highlighted in the panel at upper right. Amino acid sequences produced by frameshifts are represented in green.

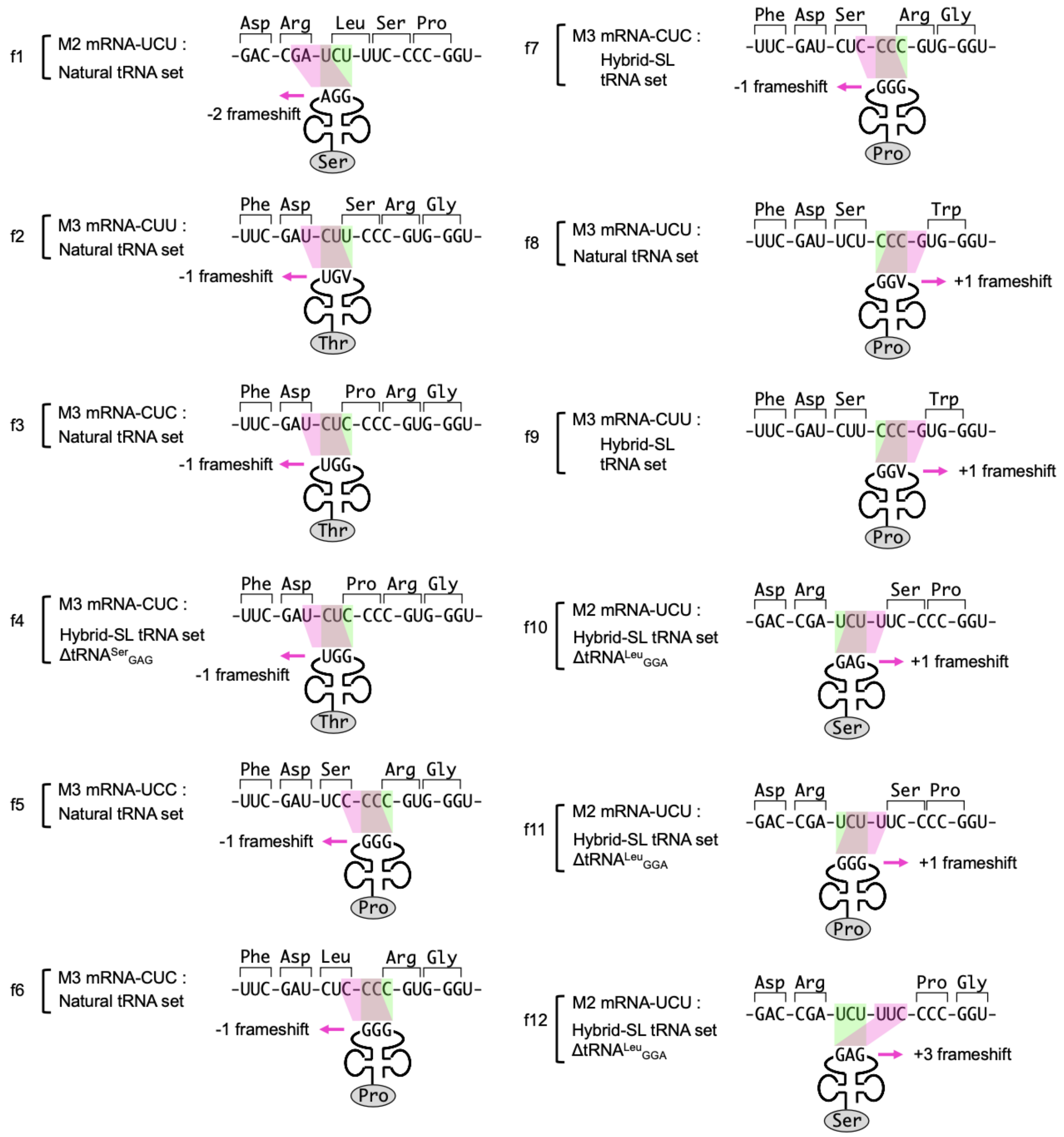

Supplementary Fig. 31. Possible mechanisms to induce frameshifts resulting in the production of f1–f12 peptides. Possible codon/anticodon pairs before and after the frameshift are indicated by green and magenta bands, respectively.

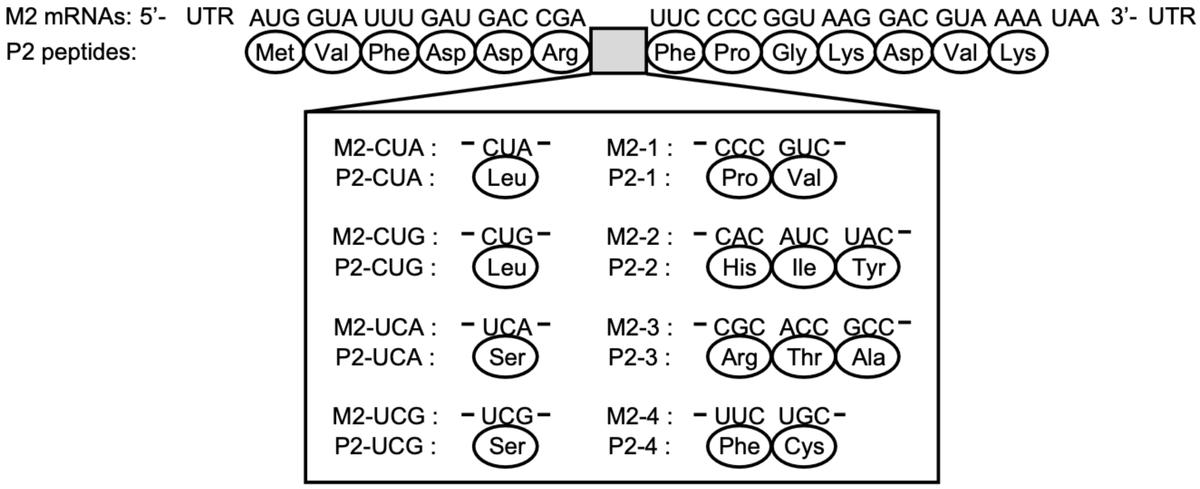

Supplementary Fig. 32. M2 mRNAs to study the fidelity of chimeric tRNAs.

Codons	Incorporated amino acids	MS/MS spectrum No.	
		Natural tRNA extract	Natural tRNA extract + chimeric tRNAs
UUC	Phe	ms50	ms51
	Ser	ms52	ms53
CCC	Pro	ms54	ms55
	Leu	ms56	ms57
	Ser	ms58	ms59

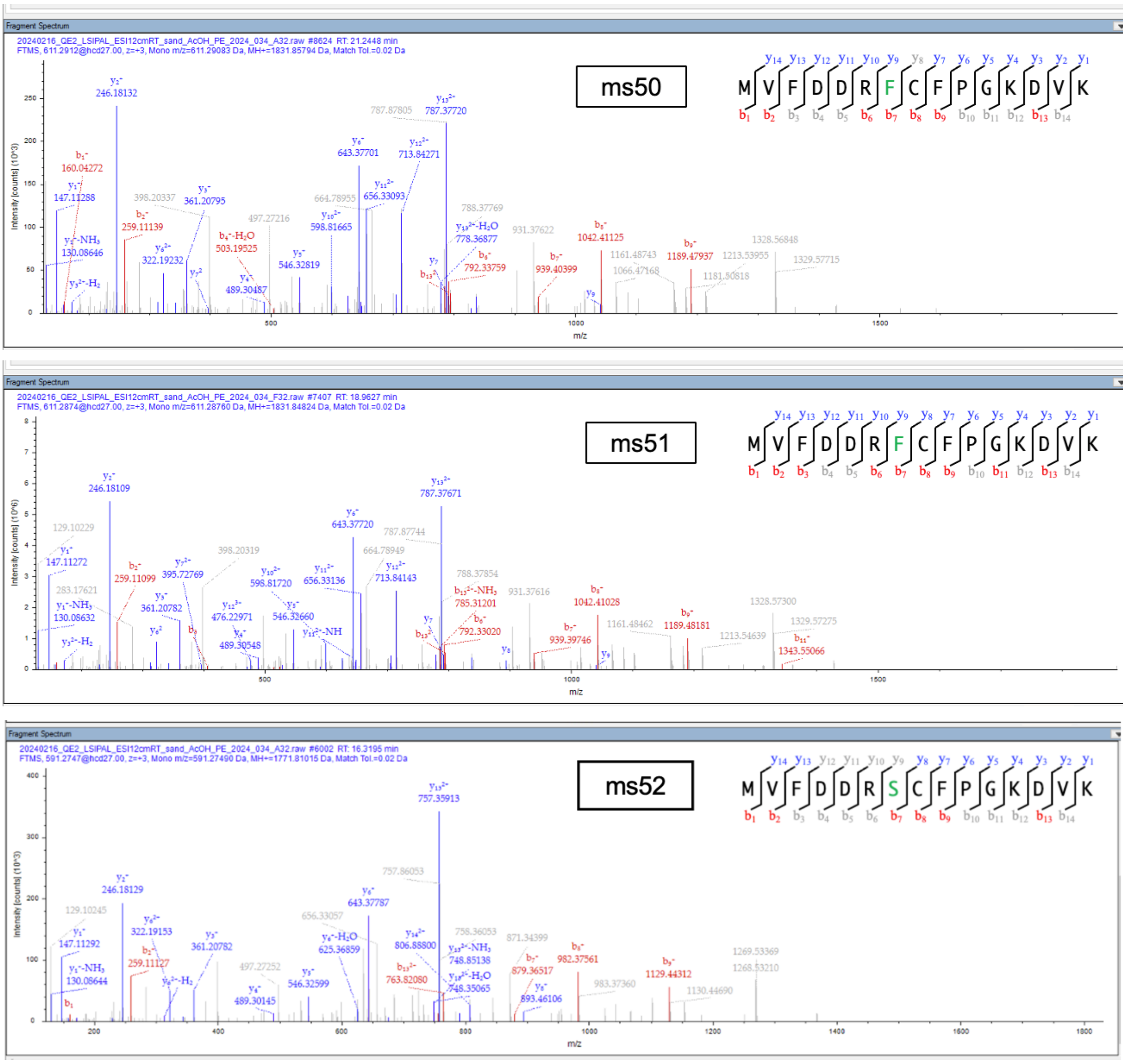

Supplementary Fig. 33. MS/MS data of the products derived from misreading of near-cognate codons on M2 mRNAs by chimeric tRNA^{Ser}$_{GAG}$ and tRNA^{Leu}$_{GGA}$ (ms50–ms52). The peaks of the y ions are represented in blue, and the b ions in red. Identified y and b ions are highlighted in the panel at upper right. Amino acids incorporated into UUC or CCC codons are represented in green.

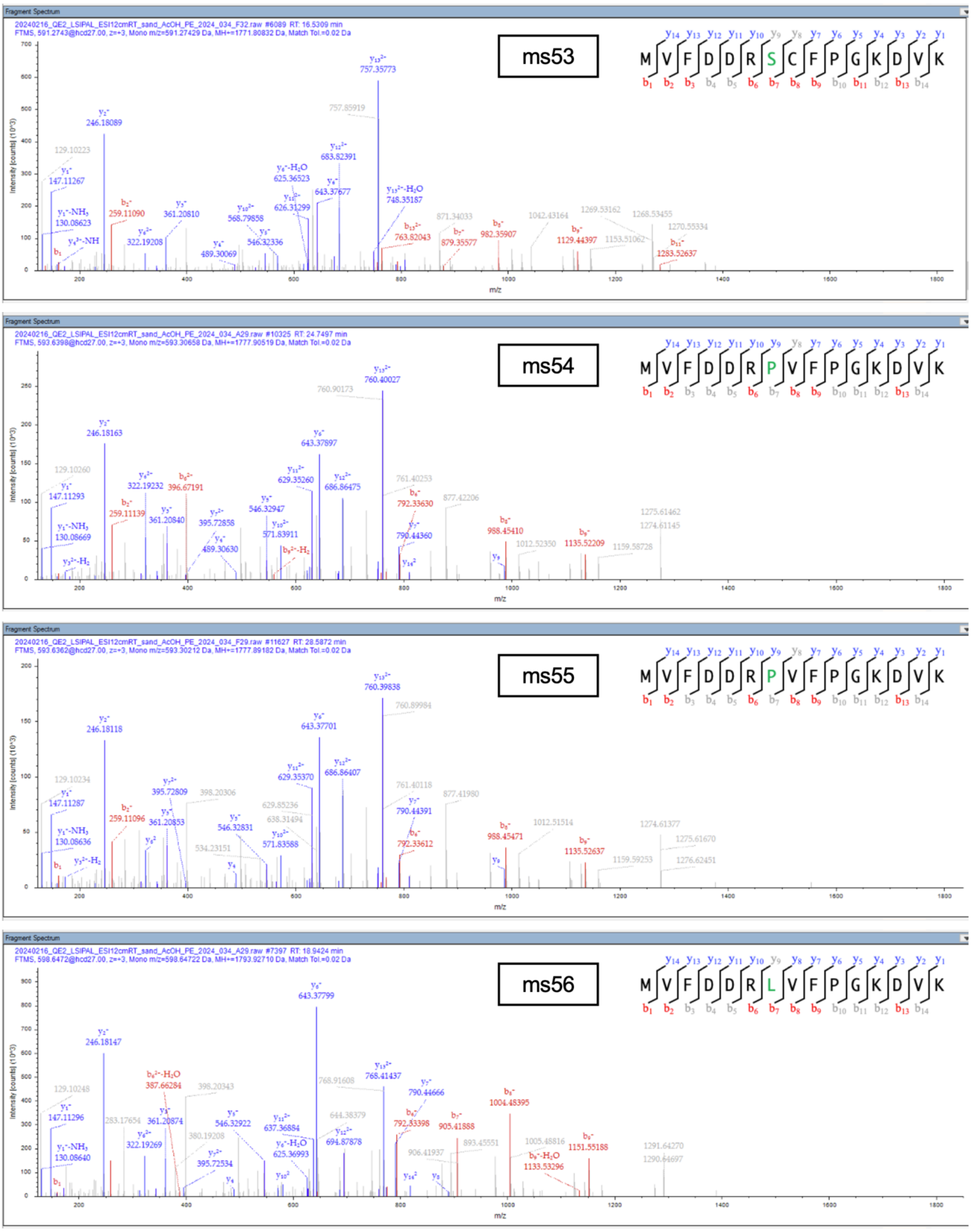

Supplementary Fig. 34. MS/MS data of the products derived from misreading of near-cognate codons on M2 mRNAs by chimeric tRNA$^{Ser}_{GAG}$ and tRNA$^{Leu}_{GGA}$ (ms53–ms56, Supplementary Fig. 33). The peaks of the y ions are represented in blue, and the b ions in red. Identified y and b ions are highlighted in the panel at upper right. Amino acids incorporated into UUC or CCC codons are represented in green.

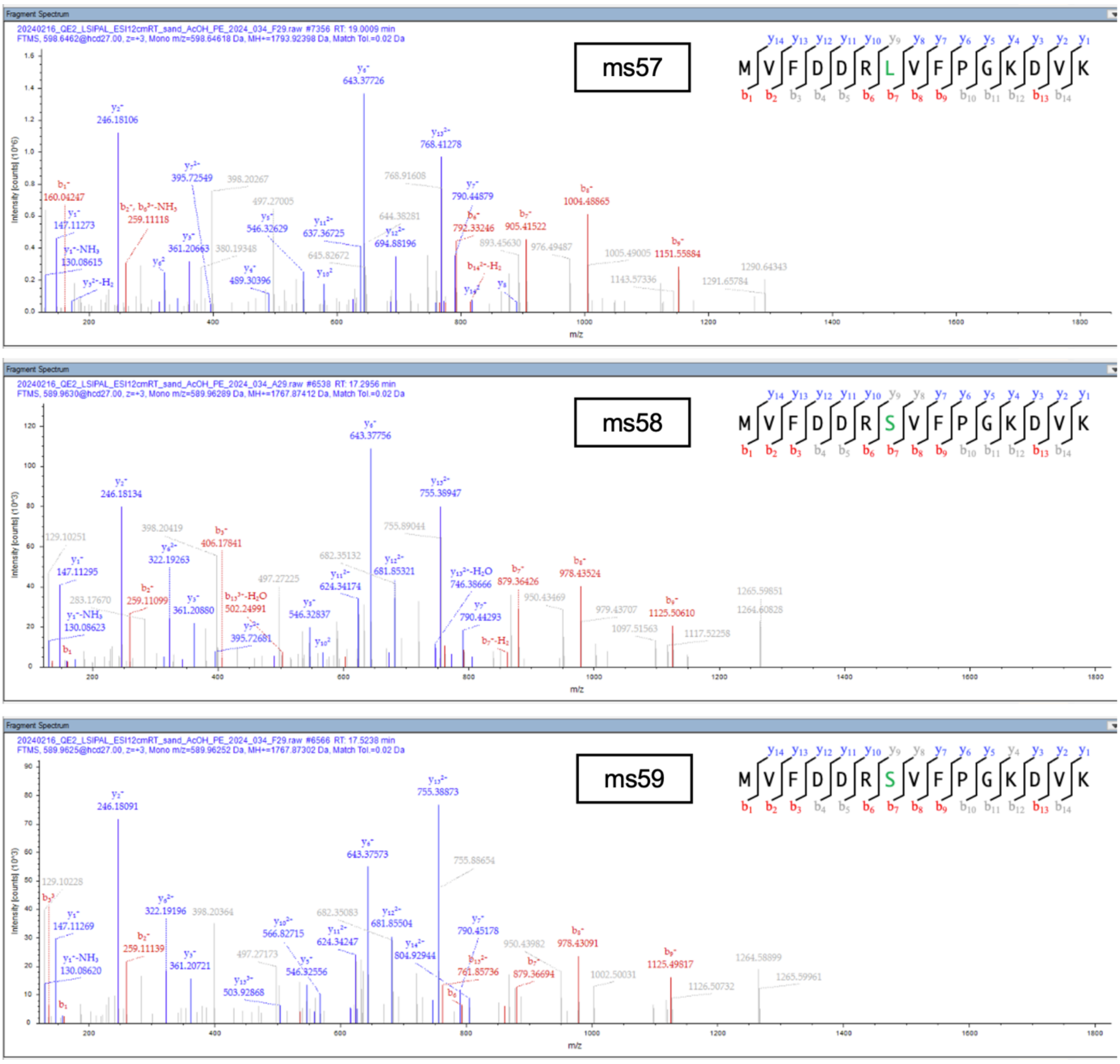

Supplementary Fig. 35. MS/MS data of the products derived from misreading of near-cognate codons on M2 mRNAs by chimeric tRNA$^{Ser}{}_{GAG}$ and tRNA$^{Leu}{}_{GGA}$ (ms57–ms59, Supplementary Fig. 33). The peaks of the y ions are represented in blue, and the b ions in red. Identified y and b ions are highlighted in the panel at upper right. Amino acids incorporated into UUC or CCC codons are represented in green.

```
                   Met Asn Met Lys Gly Val Ala Met Pro Gly Ala Glu Asp Asp Val Val Lys Ser Lys Gly
pa-gfp-Std     1 ATG AAT ATG AAA GGC GTT GCG ATG CCT GGT GCC GAA GAT GAT GTC GTG AAA TCT AAA GGC 60
pa-gfp-SL      1 ATG AAT ATG AAA GGC GTT GCG ATG CCT GGT GCC GAA GAT GAT GTC GTG AAA CTT AAA GGC 60

                   Glu Glu Leu Phe Thr Gly Val Val Pro Ile Leu Val Glu Leu Asp Gly Asp Val Asn Gly
pa-gfp-Std    61 GAA GAA CTT TTT ACC GGT GTC GTA CCG ATT CTT GTC GAG CTT GAC GGC GAT GTG AAT GGC 120
pa-gfp-SL     61 GAA GAG TCT TTT ACG GGT GTC GTC CCC ATC TCT GTC GAA TCT GAT GGT GAC GTA AAT GGC 120

                   His Lys Phe Ser Val Arg Gly Glu Gly Glu Gly Asp Ala Thr Asn Gly Lys Leu Thr Leu
pa-gfp-Std   121 CAC AAG TTT TCT GTC CGT GGT GAA GGT GAA GGC GAT GCG ACC AAT GGG AAA CTT ACG CTT 180
pa-gfp-SL    121 CAC AAA TTT CTT GTA CGT GGA GAA GGC GAA GGT GAT GCG ACC AAT GGG AAA TCT ACC TCT 180

                   Lys Phe Ile Cys Thr Thr Gly Lys Leu Pro Val Pro Trp Pro Thr Leu Val Thr Thr Leu
pa-gfp-Std   181 AAA TTC ATC TGT ACG ACA GGC AAA CTT CCG GTA CCT TGG CCA ACT CTT GTC ACT ACG CTT 240
pa-gfp-SL    181 AAA TTC ATC TGT ACA ACC GGA AAA TCT CCG GTT CCA TGG CCT ACG TCT GTT ACG ACA TCT 240

                   Thr Tyr Gly Val Gln Cys Phe Ser Arg Tyr Pro Asp His Met Lys Arg His Asp Phe Phe
pa-gfp-Std   241 ACG TAT GGG GTT CAG TGC TTT TCT CGT TAT CCG GAT CAC ATG AAA CGG CAT GAC TTC TTT 300
pa-gfp-SL    241 ACG TAT GGC GTT CAA TGC TTT CTT CGG TAT CCG GAT CAT ATG AAG CGT CAT GAC TTC TTC 300

                   Lys Ser Ala Met Pro Glu Gly Tyr Val Gln Glu Arg Thr Ile Ser Phe Lys Asp Asp Gly
pa-gfp-Std   301 AAA TCT GCA ATG CCG GAA GGC TAT GTG CAA GAA CGC ACG ATT TCT TTT AAA GAC GAC GGT 360
pa-gfp-SL    301 AAA CTT GCG ATG CCT GAA GGG TAT GTG CAG GAA CGC ACC ATC CTT TTC AAA GAC GAT GGC 360

                   Thr Tyr Lys Thr Arg Ala Glu Val Lys Phe Glu Gly Asp Thr Leu Val Asn Arg Ile Glu
pa-gfp-Std   361 ACC TAC AAG ACT CGT GCC GAA GTT AAG TTC GAA GGT GAT ACC CTT GTG AAT CGC ATC GAG 420
pa-gfp-SL    361 ACG TAT AAA ACC CGT GCA GAA GTG AAA TTT GAG GGC GAT ACC TCT GTG AAT CGC ATT GAA 420

                   Leu Lys Gly Ile Asp Phe Lys Glu Asp Gly Asn Ile Leu Gly His Lys Leu Glu Tyr Asn
pa-gfp-Std   421 CTT AAA GGC ATC GAT TTC AAG GAA GAT GGG AAC ATC CTT GGT CAT AAA CTT GAG TAT AAC 480
pa-gfp-SL    421 TCT AAA GGC ATC GAC TTC AAA GAA GAT GGC AAC ATT TCT GGT CAC AAG TCT GAG TAC AAC 480

                   Phe Asn Ser His Asn Val Tyr Ile Thr Ala Asp Lys Gln Lys Asn Gly Ile Lys Ala Asn
pa-gfp-Std   481 TTC AAT TCT CAT AAC GTG TAC ATT ACC GCG GAC AAA CAG AAG AAC GGT ATC AAA GCC AAC 540
pa-gfp-SL    481 TTT AAC CTT CAT AAC GTG TAC ATT ACT GCC GAT AAA CAG AAG AAC GGT ATC AAA GCC AAC 540

                   Phe Lys Ile Arg His Asn Val Glu Asp Gly Ser Val Gln Leu Ala Asp His Tyr Gln Gln
pa-gfp-Std   541 TTT AAA ATT CGC CAT AAC GTG GAA GAT GGT TCT GTT CAG CTT GCG GAC CAC TAT CAG CAG 600
pa-gfp-SL    541 TTT AAG ATT CGC CAT AAT GTG GAA GAT GGC CTT GTG CAG TCT GCT GAC CAT TAC CAG CAG 600

                   Asn Thr Pro Ile Gly Asp Gly Pro Val Leu Leu Pro Asp Asn His Tyr Leu Ser Thr Gln
pa-gfp-Std   601 AAT ACC CCT ATT GGC GAT GGA CCA GTT CTT CTT CCC GAT AAC CAC TAC CTT TCT ACC CAA 660
pa-gfp-SL    601 AAC ACT CCG ATT GGG GAT GGT CCG GTA TCT TCT CCA GAC AAT CAC TAC TCT CTT ACC CAA 660

                   Ser Val Leu Ser Lys Asp Pro Asn Glu Lys Arg Asp His Met Val Leu Leu Glu Phe Val
pa-gfp-Std   661 TCT GTA CTT TCT AAA GAC CCG AAT GAG AAA CGC GAT CAT ATG GTG CTT CTT GAG TTT GTG 720
pa-gfp-SL    661 CTT GTT TCT CTT AAA GAC CCG AAT GAG AAA CGC GAT CAC ATG GTG TCT TCT GAA TTT GTC 720

                   Thr Ala Ala Gly Ile Thr His Gly Met Asp Glu Leu Tyr Lys stop
pa-gfp-Std   721 ACA GCA GCT GGA ATT ACC CAT GGC ATG GAT GAA CTT TAC AAA TAA                    765
pa-gfp-SL    721 ACT GCA GCG GGT ATT ACC CAT GGT ATG GAT GAG TCT TAT AAG TAA                    765
```

pa-gfp-Std

Phe	UUU	7	Ser	UCU	10	Tyr	UAU	5	Cys	UGU	1
	UUC	5		UCC	0		UAC	4		UGC	1
Leu	UUA	0		UCA	0	stop	UAA	1	stop	UGA	0
	UUG	0		UCG	0		UAG	0	Trp	UGG	1
	CUU	20	Pro	CCU	3	His	CAU	6	Arg	CGU	3
	CUC	0		CCC	1		CAC	4		CGC	4
	CUA	0		CCA	2	Gln	CAA	2		CGA	0
	CUG	0		CCG	5		CAG	5		CGG	1
Ile	AUU	6	Thr	ACU	3	Asn	AAU	7	Ser	AGU	0
	AUC	5		ACC	8		AAC	7		AGC	0
	AUA	0		ACA	2	Lys	AAA	17	Arg	AGA	0
Met	AUG	7		ACG	5		AAG	5		AGG	0
Val	GUU	5	Ala	GCU	1	Asp	GAU	13	Gly	GGU	9
	GUC	5		GCC	3		GAC	7		GGC	10
	GUA	3		GCA	2	Glu	GAA	12		GGA	2
	GUG	8		GCG	4		GAG	5		GGG	3

pa-gfp-SL

Phe	UUU	7	Ser	UCU	20	Tyr	UAU	5	Cys	UGU	1
	UUC	5		UCC	0		UAC	4		UGC	1
Leu	UUA	0		UCA	0	stop	UAA	1	stop	UGA	0
	UUG	0		UCG	0		UAG	0	Trp	UGG	1
	CUU	10	Pro	CCU	3	His	CAU	6	Arg	CGU	3
	CUC	0		CCC	1		CAC	4		CGC	4
	CUA	0		CCA	2	Gln	CAA	2		CGA	0
	CUG	0		CCG	5		CAG	5		CGG	1
Ile	AUU	6	Thr	ACU	3	Asn	AAU	7	Ser	AGU	0
	AUC	5		ACC	8		AAC	7		AGC	0
	AUA	0		ACA	2	Lys	AAA	17	Arg	AGA	0
Met	AUG	7		ACG	5		AAG	5		AGG	0
Val	GUU	5	Ala	GCU	1	Asp	GAU	13	Gly	GGU	9
	GUC	5		GCC	3		GAC	7		GGC	10
	GUA	3		GCA	2	Glu	GAA	12		GGA	2
	GUG	8		GCG	4		GAG	5		GGG	3

Supplementary Fig. 36. The sequences of *pa-gfp-Std, pa-gfp-SL* and their codon usages. Ser and Leu codons are highlighted in green.

```
                 Met Asn Met Lys Gly Val Ala Met Pro Gly Ala Glu Asp Asp Val Val Lys Asp Pro Ser
pa-stv-Std     1 ATG AAT ATG AAA GGC GTT GCG ATG CCT GGT GCC GAA GAT GAT GTC GTG AAA GAC CCG TCT 60
pa-stv-SL      1 ATG AAT ATG AAA GGC GTT GCG ATG CCT GGT GCC GAA GAT GAT GTC GTG AAA GAC CCG CTT 60

                 Lys Asp Ser Lys Ala Gln Val Ser Ala Ala Glu Ala Gly Ile Thr Gly Thr Trp Tyr Asn
pa-stv-Std    61 AAA GAC TCT AAA GCC CAG GTT TCT GCC GCA GAA GCG GGC ATT ACA GGC ACA TGG TAC AAC 120
pa-stv-SL     61 AAA GAC CTT AAA GCC CAG GTT CTT GCC GCA GAA GCG GGC ATT ACA GGC ACA TGG TAC AAC 120

                 Gln Leu Gly Ser Thr Phe Ile Val Thr Ala Gly Ala Asp Gly Ala Leu Thr Gly Thr Tyr
pa-stv-Std   121 CAG CTT GGT TCT ACC TTT ATC GTG ACG GCA GGA GCT GAT GGC GCT CTT ACT GGC ACC TAT 180
pa-stv-SL    121 CAG TCT GGT CTT ACC TTT ATC GTG ACG GCA GGA GCT GAT GGC GCT TCT ACT GGC ACC TAT 180

                 Glu Ser Ala Val Gly Asn Ala Glu Ser Arg Tyr Val Leu Thr Gly Arg Tyr Asp Ser Ala
pa-stv-Std   181 GAG TCT GCA GTG GGC AAT GCG GAG TCT CGC TAT GTG CTT ACT GGG CGT TAT GAC TCT GCA 240
pa-stv-SL    181 GAG CTT GCA GTG GGC AAT GCG GAG CTT CGC TAT GTG TCT ACT GGG CGT TAT GAC CTT GCA 240

                 Pro Ala Thr Asp Gly Ser Gly Thr Ala Leu Gly Trp Thr Val Ala Trp Lys Asn Asn Tyr
pa-stv-Std   241 CCA GCG ACC GAT GGT TCT GGT ACT GCC CTT GGT TGG ACC GTT GCG TGG AAG AAC AAC TAC 300
pa-stv-SL    241 CCA GCG ACC GAT GGT CTT GGT ACT GCC TCT GGT TGG ACC GTT GCG TGG AAG AAC AAC TAC 300

                 Arg Asn Ala His Ser Ala Thr Thr Trp Ser Gly Gln Tyr Val Gly Gly Ala Glu Ala Arg
pa-stv-Std   301 CGG AAT GCC CAT TCT GCC ACC ACG TGG TCT GGC CAG TAC GTA GGA GGC GCT GAA GCG CGT 360
pa-stv-SL    301 CGG AAT GCC CAT CTT GCC ACC ACG TGG CTT GGC CAG TAC GTA GGA GGC GCT GAA GCG CGT 360

                 Ile Asn Thr Gln Trp Leu Leu Thr Ser Gly Thr Thr Glu Ala Asn Ala Trp Lys Ser Thr
pa-stv-Std   361 ATC AAC ACC CAA TGG CTT CTT ACC TCT GGG ACA ACG GAA GCC AAC GCA TGG AAA TCT ACG 420
pa-stv-SL    361 ATC AAC ACC CAA TGG TCT TCT ACC CTT GGG ACA ACG GAA GCC AAC GCA TGG AAA CTT ACG 420

                 Leu Val Gly His Asp Thr Phe Thr Lys Val Lys Pro Ser Ala Ala Ser Ile Asp Ala Ala
pa-stv-Std   421 CTT GTT GGT CAC GAC ACC TTC ACG AAA GTC AAA CCC TCT GCT GCG TCT ATT GAT GCG GCG 480
pa-stv-SL    421 TCT GTT GGT CAC GAC ACC TTC ACG AAA GTC AAA CCC CTT GCT GCG CTT ATT GAT GCG GCG 480

                 Lys Lys Ala Gly Val Asn Asn Gly Asn Pro Leu Asp Ala Val Gln Glnstop
pa-stv-Std   481 AAG AAA GCA GGG GTG AAC AAT GGC AAT CCT CTT GAT GCC GTC CAA CAG TAA             531
pa-stv-SL    481 AAG AAA GCA GGG GTG AAC AAT GGC AAT CCT TCT GAT GCC GTC CAA CAG TAA             531
```

pa-stv-Std

Phe	UUU	1	Ser	UCU	14	Tyr	UAU	3	Cys	UGU	0
	UUC	1		UCC	0		UAC	3		UGC	0
Leu	UUA	0		UCA	0	stop	UAA	1	stop	UGA	0
	UUG	0		UCG	0		UAG	0	Trp	UGG	6
	CUU	8	Pro	CCU	2	His	CAU	1	Arg	CGU	2
	CUC	0		CCC	1		CAC	1		CGC	1
	CUA	0		CCA	1	Gln	CAA	2		CGA	0
	CUG	0		CCG	1		CAG	4		CGG	1
Ile	AUU	2	Thr	ACU	3	Asn	AAU	5	Ser	AGU	0
	AUC	2		ACC	8		AAC	6		AGC	0
	AUA	0		ACA	3	Lys	AAA	8	Arg	AGA	0
Met	AUG	3		ACG	5		AAG	2		AGG	0
Val	GUU	4	Ala	GCU	4	Asp	GAU	6	Gly	GGU	6
	GUC	3		GCC	8		GAC	4		GGC	9
	GUA	1		GCA	6	Glu	GAA	4		GGA	2
	GUG	5		GCG	9		GAG	2		GGG	3

pa-stv-SL

Phe	UUU	1	Ser	UCU	8	Tyr	UAU	3	Cys	UGU	0
	UUC	1		UCC	0		UAC	3		UGC	0
Leu	UUA	0		UCA	0	stop	UAA	1	stop	UGA	0
	UUG	0		UCG	0		UAG	0	Trp	UGG	6
	CUU	14	Pro	CCU	2	His	CAU	1	Arg	CGU	2
	CUC	0		CCC	1		CAC	1		CGC	1
	CUA	0		CCA	1	Gln	CAA	2		CGA	0
	CUG	0		CCG	1		CAG	4		CGG	1
Ile	AUU	2	Thr	ACU	3	Asn	AAU	5	Ser	AGU	0
	AUC	2		ACC	8		AAC	6		AGC	0
	AUA	0		ACA	3	Lys	AAA	8	Arg	AGA	0
Met	AUG	3		ACG	5		AAG	2		AGG	0
Val	GUU	4	Ala	GCU	4	Asp	GAU	6	Gly	GGU	6
	GUC	3		GCC	8		GAC	4		GGC	9
	GUA	1		GCA	6	Glu	GAA	4		GGA	2
	GUG	5		GCG	9		GAG	2		GGG	3

Supplementary Fig. 37. The sequences of *pa-stv-Std, pa-stv-SL* genes used for LC-MS analysis. Ser and Leu codons are highlighted in green.

Proteins	Positions	Genes	Codons	tRNA sets	MS/MS spectrum No. Incorporated amino acids :	
					Leu	Ser
sfGFP	135	*pa-gfp-Std*	CUU	Natural tRNA extract	ms60	
		pa-gfp-SL	UCU	Hybrid-SL tRNA set	ms61	
	102	*pa-gfp-Std*	UCU	Natural tRNA extract		ms68
		pa-gfp-SL	CUU	Hybrid-SL tRNA set	ms70	ms69
Streptavidin	171	*pa-stv-Std*	CUU	Natural tRNA extract	ms62	
		pa-stv-SL	UCU	Hybrid-SL tRNA set	ms63	
β-galactosidase	41	*pa-gal-Std*	CUU	Natural tRNA extract	ms64	
		pa-gal-SL	UCU	Hybrid-SL tRNA set	ms65	
	424	*pa-gal-Std*	CUU	Natural tRNA extract	ms66	
		pa-gal-SL	UCU	Hybrid-SL tRNA set	ms67	

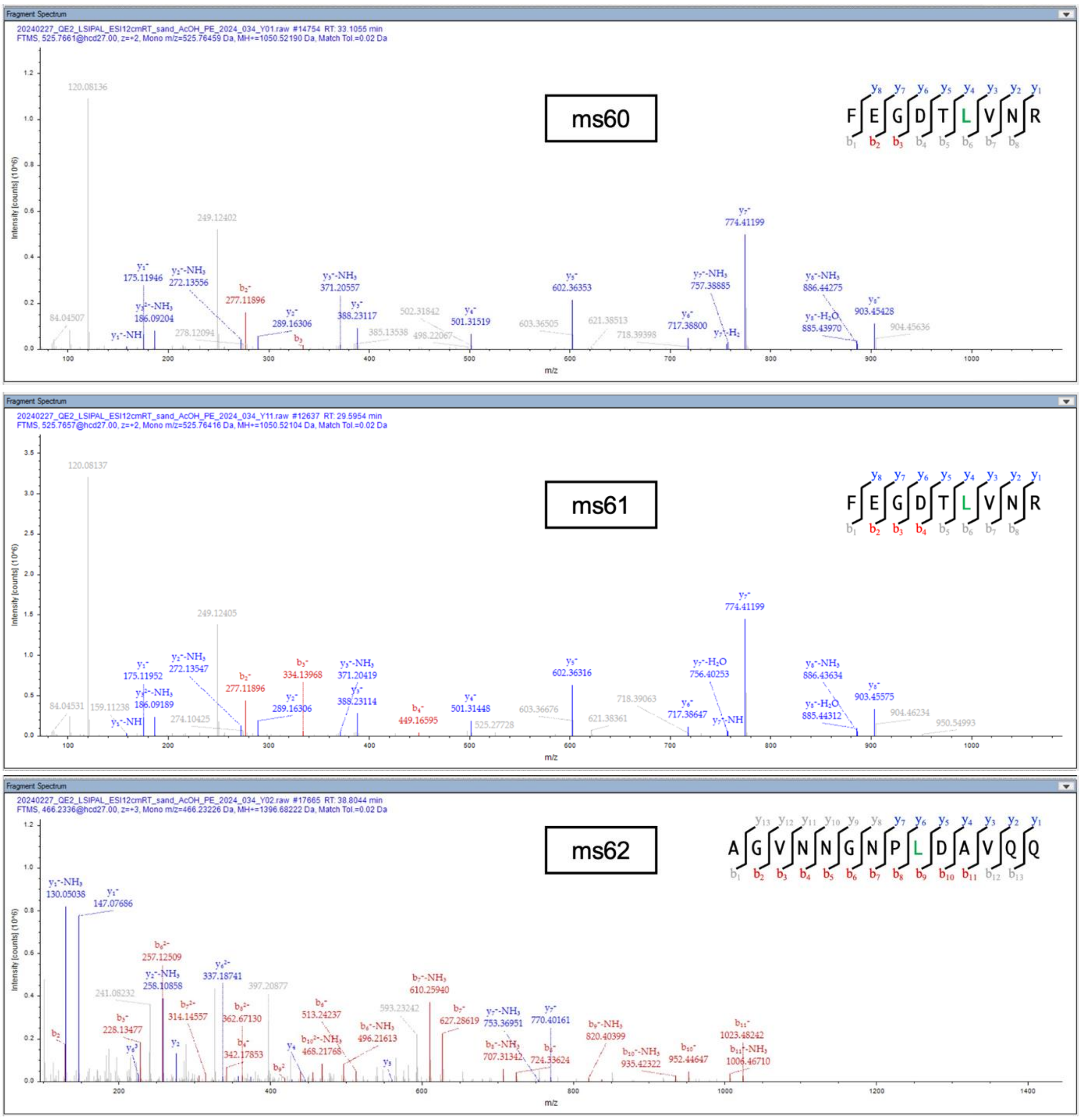

Supplementary Fig. 38. MS/MS data of peptide fragments originating from proteins by trypsin digestion (ms60–ms62). The peaks of the y ions are represented in blue, and the b ions in red. Identified y and b ions are highlighted in the panel at upper right. Amino acids incorporated into CUU or UCU codons are represented in green.

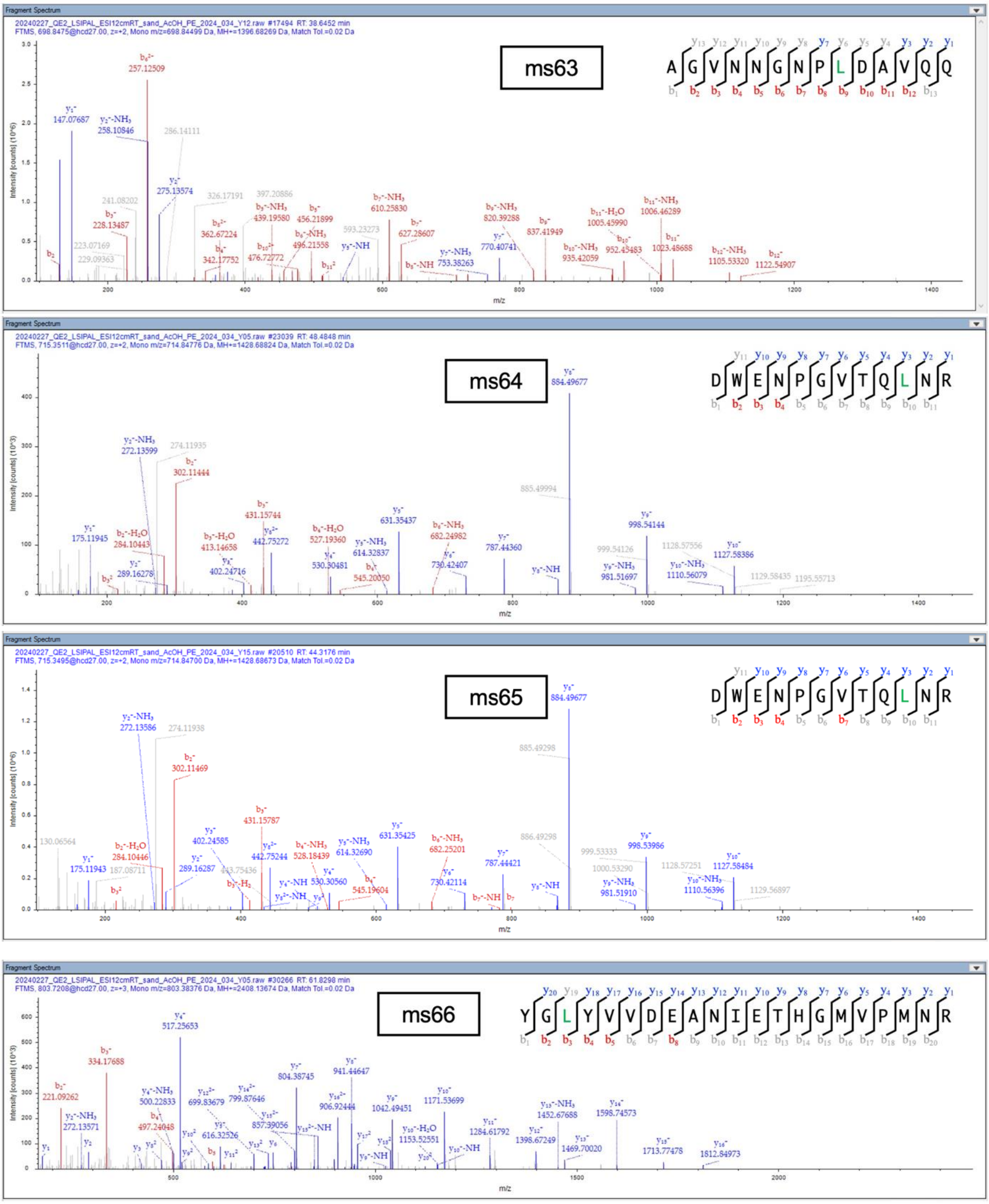

Supplementary Fig. 39. MS/MS data of peptide fragments originating from proteins by trypsin digestion (ms63–ms66, Supplementary Fig. 38). The peaks of the y ions are represented in blue, and the b ions in red. Identified y and b ions are highlighted in the panel at upper right. Amino acids incorporated into CUU or UCU codons are represented in green.

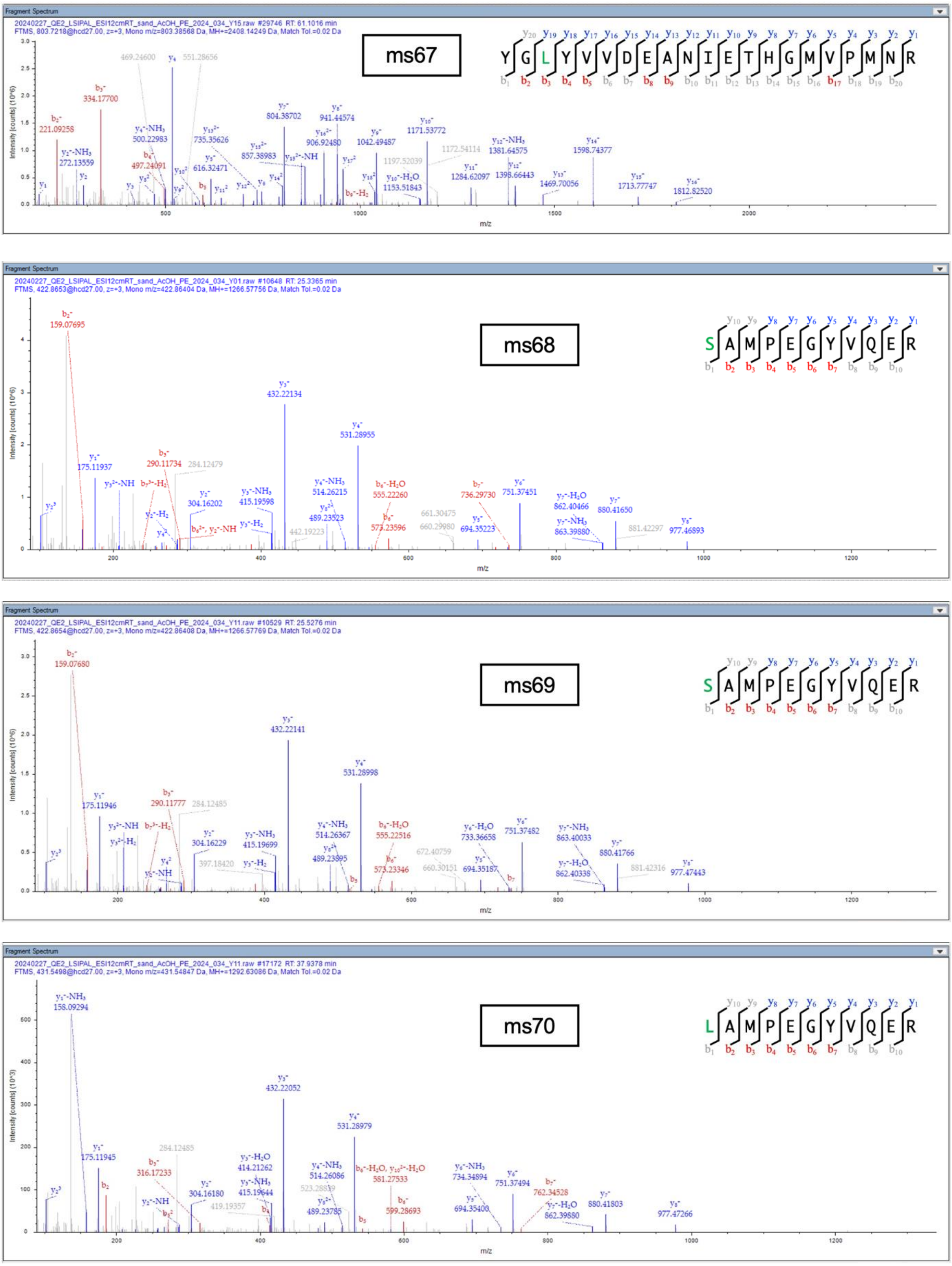

Supplementary Fig. 40. MS/MS data of peptide fragments originating from proteins by trypsin digestion (ms67–ms70, Supplementary Fig. 38). The peaks of the y ions are represented in blue, and the b ions in red. Identified y and b ions are highlighted in the panel at upper right. Amino acids incorporated into CUU or UCU codons are represented in green.

Supplementary Table 1. Calculated and observed mass of model peptides. Abbreviations: Std, standard; SL, Ser/Leu-swapped.

	NNN =	
	UCU	CUU
Hybrid-Std tRNA set	X= Ser: Calculated mass: 1255.45; Observed mass: 1255.46	X= Ser: Calculated mass: 1255.45; Observed mass: −
	X= Leu: Calculated mass: 1281.50; Observed mass: −	X= Leu: Calculated mass: 1281.50; Observed mass: 1281.49
Hybrid-SL tRNA set	X= Ser: Calculated mass: 1255.45; Observed mass: −	X= Ser: Calculated mass: 1255.45; Observed mass: 1255.58
	X= Leu: Calculated mass: 1281.50; Observed mass: 1281.63	X= Leu: Calculated mass: 1281.50; Observed mass: −
E. coli natural tRNA extract + $tRNA^{Ser}_{GAG}$ $tRNA^{Leu}_{GGA}$	X= Ser: Calculated mass: 1255.45; Observed mass: 1255.52	X= Ser: Calculated mass: 1255.45; Observed mass: 1255.54
	X= Leu: Calculated mass: 1281.50; Observed mass: 1281.57	X= Leu: Calculated mass: 1281.50; Observed mass: 1281.59

Supplementary Table 2. Calculated and observed masses of model peptides and their subproducts. Symbols (*, ¶, §, †, ††) in the table correspond to the peak labels in Fig. 3b, and Supplementary Fig. 3a and 4.

Codons	Xaa	Full-length peptides (*)		Sub-products	
		Calculated mass ($M+H^+$)	Observed mass	Types	Observed mass
GCU	Ala	1239.45	1239.54		
AAU	Asn	1282.46	1282.58		
UGU	Cys	1271.42	1271.71	Addition of β-mercaptoethanol (¶)	1347.74
				Addition of Cys (§)	1390.76
CAG	Gln	1296.47	1296.60		
GAG	Glu	1297.46	1298.00	+163 by-product corresponding to an added Tyr (†)	1461.09
GGU	Gly	1225.44	1225.49		
CAU	His	1305.47	1305.56		
AUU	Ile	1281.50	1281.58		
AAG	Lys	1296.51	1297.08	+163 by-product corresponding to an added Tyr (†)	1460.14
AUG	Met	1299.46	1299.71		
UUU	Phe	1315.48	1315.70	+163 by-product corresponding to an added Tyr (†)	1478.80
				+326 by-product corresponding to two additional Tyr residues (††)	1641.91
CCU	Pro	1265.47	1265.58		
ACU	Thr	1269.46	1269.64		
UGG	Trp	1354.49	1354.73	+163 by-product corresponding to an added Tyr (†)	1517.83
GUU	Val	1267.48	1267.75		

Supplementary Table 3. The relative abundance of amino acids incorporated in model peptide P2 shown in Fig. 5a. The relative abundance is represented by a color gradient ranging from white (lowest) to magenta (highest). The MS/MS spectra shown in the table are provided in Supplementary Fig. 16–25. Source data are provided as a Source Data file.

Codons	CUU						CUC					
tRNA sets	Natural tRNA		hybrid SL-swap		hybrid SL $\Delta tRNA^{Ser}_{GAG}$		Natural tRNA		hybrid SL-swap		hybrid SL $\Delta tRNA^{Ser}_{GAG}$	
Amino acids	Relative abundance (%)	MS/MS spectrum No.	Relative abundance (%)	MS/MS spectrum No.	Relative abundance (%)	MS/MS spectrum No.	Relative abundance (%)	MS/MS spectrum No.	Relative abundance (%)	MS/MS spectrum No.	Relative abundance (%)	MS/MS spectrum No.
Phe	0.04		0.03		2.65	ms9	0.07		0.03		4.51	ms10
Leu	99.75		1.23	ms5	96.24		99.79		0.84	ms6	93.80	
Ser	0.12		97.67	ms1	0.02		0.03		98.12	ms2	0.05	
Thr	0.09		1.07		1.09	ms14	0.10		1.01		1.64	ms15

Codons	UCU						UCC					
tRNA sets	Natural tRNA		hybrid SL-swap		hybrid SL $\Delta tRNA^{Leu}_{GGA}$		Natural tRNA		hybrid SL-swap		hybrid SL $\Delta tRNA^{Leu}_{GGA}$	
Amino acids	Relative abundance (%)	MS/MS spectrum No.	Relative abundance (%)	MS/MS spectrum No.	Relative abundance (%)	MS/MS spectrum No.	Relative abundance (%)	MS/MS spectrum No.	Relative abundance (%)	MS/MS spectrum No.	Relative abundance (%)	MS/MS spectrum No.
Phe	0.01		0.04		1.65	ms28	0.01		0.04		3.91	ms29
Leu	0.02		99.57	ms20	0.50		0.02		99.69	ms21	0.05	
Ser	97.48		0.34	ms24	93.81		98.53		0.17	ms25	90.09	
Thr	2.49		0.06		4.04	ms32	1.44		0.10		5.95	ms33

Supplementary Table 4. The relative abundance of amino acids incorporated in model peptide P3 shown in Fig. 5b. The relative abundance is represented by a color gradient ranging from white (lowest) to magenta (highest). The MS/MS spectra shown in the table are provided in Supplementary Fig. 16–25. Source data are provided as a Source Data file.

Codons	CUU						CUC					
tRNA sets	Natural tRNA		hybrid SL-swap		hybrid SL $\Delta tRNA^{Ser}_{GAG}$		Natural tRNA		hybrid SL-swap		hybrid SL $\Delta tRNA^{Ser}_{GAG}$	
Amino acids	Relative abundance (%)	MS/MS spectrum No.	Relative abundance (%)	MS/MS spectrum No.	Relative abundance (%)	MS/MS spectrum No.	Relative abundance (%)	MS/MS spectrum No.	Relative abundance (%)	MS/MS spectrum No.	Relative abundance (%)	MS/MS spectrum No.
His	N.D.		N.D.		N.D.		0.07		0.01		12.54	ms11
Leu	99.15		0.16	ms7	17.38		99.60		0.09	ms8	3.34	
Pro	0.09		0.00		8.85	ms12	0.05		0.00		17.03	ms13
Ser	0.22		84.44	ms3	0.00		0.09		79.53	ms4	0.00	
Thr	0.54		15.39	ms18	73.77	ms16	0.19		20.37	ms19	67.09	ms17
Codons	UCU						UCC					
tRNA sets	Natural tRNA		hybrid SL-swap		hybrid SL $\Delta tRNA^{Leu}_{GGA}$		Natural tRNA		hybrid SL-swap		hybrid SL $\Delta tRNA^{Leu}_{GGA}$	
Amino acids	Relative abundance (%)	MS/MS spectrum No.	Relative abundance (%)	MS/MS spectrum No.	Relative abundance (%)	MS/MS spectrum No.	Relative abundance (%)	MS/MS spectrum No.	Relative abundance (%)	MS/MS spectrum No.	Relative abundance (%)	MS/MS spectrum No.
His	N.D.		N.D.		N.D.		N.D.		N.D.		N.D.	
Leu	0.00		98.54	ms22	0.00		0.01		98.14	ms23	0.00	
Pro	0.01		0.09		19.84	ms30	0.09		0.07		17.73	ms31
Ser	89.05		0.65	ms26	50.64		75.28		1.00	ms27	26.95	
Thr	10.95	ms 34	0.72		29.52	ms35	24.62	ms36	0.78		55.31	ms37

Supplementary Table 5. Sequence of T7-tRNA used in this study. Source data are provided as a Source Data file.

Names	Anticodons	Length	tRNA sequences (5′to 3′)
$tRNA^{Ala}$	GGC	76	GGGGC UAUAG CUCAG CUGGG AGAGC GCUUG CAUGG CAUGC AAGAG GUCAG CGGUU CGAUC CCGCU UAGCU CCACC A
$tRNA^{Arg}$	GCG	77	GCAUC CGUAG CUCAG CUGGA UAGAG UACUC GGCUG CGAAC CGAGC GGUCG GAGGU UCGAA UCCUC CCGGA UGCAC CA
$tRNA^{Asn}$	GUU	76	GCCUC UGUAG UUCAG UCGGU AGAAC GGCGG ACUGU UAAUC CGUAU GUCAC UGGUU CGAGU CCAGU CAGAG GCGCC A
$tRNA^{Asp}$	GUC	77	GGAGC GGUAG UUCAG UCGGU UAGAA UACCU GCCUG UCACG CAGGG GGUCG CGGGU UCGAG UCCCG UCCGU UCCGC CA
$tRNA^{Cys}$	GCA	74	GGCGC GUUAA CAAAG CGGUU AUGUA GCGGA UUGCA AAUCC GUCUA GUCCG GUUCG ACUCC GGAAC GCGCC UCCA
$tRNA^{Glu}$	CUC	76	GUCCC CUUCG UCUAG AGGCC CAGGA CACCG CCCUC UCACG GCGGU AACAG GGGUU CGAAU CCCCU AGGGG ACGCC A
$tRNA^{Gly}$	GCC	76	GCGGG AAUAG CUCAG UUGGU AGAGC ACGAC CUUGC CAAGG UCGGG GUCGC GAGUU CGAGU CUCGU UUCCC GCUCC A
$tRNA^{His}$	GUG	77	GGUGG CUAUA GCUCA GUUGG UAGAG CCCUG GAUUG UGAUU CCAGU UGUCG UGGGU UCGAA UCCCA UUAGC CACCC CA
$tRNA^{Ile}$	GAU	77	GGGCU UGUAG CUCAG GUGGU UAGAG CGCAC CCCUG AUAAG GGUGA GGUCG GUGGU UCAAG UCCAC UCAGG CCCAC CA
$tRNA^{Leu}_{GGA}$	GGA	87	GCCGA GGUGG UGGAA UUGGU AGACA CGCUA CCCUG GAAAG GUAGU GCCCA AUAGG GCUUA CGGGU UCAAG UCCCG UCCUC GGUAC CA
$tRNA^{Lys}$	CUU	76	GGGUC GUUAG CUCAG UUGGU AGAGC AGUUG ACUCU UAAUC AAUUG GUCGC AGGUU CGAAU CCUGC ACGAC CCACC A
$tRNA^{fMet}$	CAU	77	GGCGG GGUGG AGCAG CCUGG UAGCU CGUCG GGCUC AUAAC CCGAA GAUCG UCGGU UCAAA UCCGG CCCCC GCAAC CA
$tRNA^{Met}$	CAU	76	GGCUA CGUAG CUCAG UUGGU UAGAG CACAU CACUC AUAAU GAUGG GGUCA CAGGU UCGAA UCCCG UCGUA GCCAC CA
$tRNA^{Phe}$	GAA	76	GCCCG GAUAG CUCAG UCGGU AGAGC AGGGG ACUGA AAAUC CCCGU GUCCU UGGUU CGAUU CCGAG UCCGG GCACC A
$tRNA^{Ser}_{GAG}$	GAG	90	GGAGA GAUGC CGGAG CGGCU GAACG GACCG GUUUG AGGUA CCGGA GUAGG GGCAA CUCUA CCGGG GGUUC AAAUC CCCCU CUCUC CGCCA
$tRNA^{Thr}$	GGU	76	GCUGA UAUAG CUCAG UUGGU AGAGC GCACC CUUGG UAAGG GUGAG GUCGG CAGUU CGAAU CUGCC UAUCA GCACC A
$tRNA^{Tyr}$	GUA	85	GGUGG GGUUC CCGAG CGGCC AAAGG GAGCA GACUG UAAAU CUGCC GUCAC AGACU UCGAA GGUUC GAAUC CUUCC CCCAC CACCA
$tRNA^{Val}$	GAC	77	GCGUC CGUAG CUCAG UUGGU UAGAG CACCA CCUUG ACAUG GUGGG GGUCG GUGGU UCGAG UCCAC UCGGA CGCAC CA
$tRNA^{Gln}$	CUG	75	UGGGG UAUCG CCAAG CGGUA AGGCA CCGGA UUCUG AUUCC GGCAU UCCGA GGUUC GAAUC CUCGU ACCCC AGCCA
$tRNA^{Pro}$	GGG	77	CGGCA CGUAG CGCAG CCUGG UAGCG CACCG UCAUG GGGUG UCGGG GGUCG GAGGU UCAAA UCCUC UCGUG CCGAC CA
$tRNA^{Trp}$	CCA	76	AGGGG CGUAG UUCAA UUGGU AGAGC ACCGG UCUCC AAAAC CGGGU GUUGG GAGUU CGAGU CUCUC CGCCC CUGCC A
$tRNA^{Leu}$	GAG	87	GCCGA GGUGG UGGAA UUGGU AGACA CGCUA CCUUG AGGUG GUAGU GCCCA AUAGG GCUUA CGGGU UCAAG UCCCG UCCUC GGUAC CA
$tRNA^{Ser}$	GGA	88	GGUGA GGUGU CCGAG UGGCU GAAGG AGCAC GCCUG GAAAG UGUGU AUACG GCAAC GUAUC GGGGG UUCGA AUCCC CCCCU CACCG CCA

Supplementary Table 6. Composition of tRNAs used in each experiment in this study. Source data are provided as a Source Data file.

Figures	Names of tRNA sets	tRNAs									
		Chimeric tRNAs		Standard tRNAs					ΔSLY tRNA set derived from		Natural tRNA extract from MRE600 strain
		$tRNA^{Ser}$(GAG)	$tRNA^{Leu}$(GGA)	$tRNA^{Ser}$(GGA)	$tRNA^{Leu}$(GAG)	$tRNA^{Tyr}$	$tRNA^{Phe}$	other 17 tRNAs	MRE600 strain	Rosetta2 strain	
Figure 3<br>Supplementary Figure 3	Hybrid-Std			+	+	+			+		
	Hybrid-SL	+	+			+			+		
	Natural tRNA extract + chimeric $tRNA^{Ser, Lue}$	+	+								+
Figure 4<br>Supplementary Figure 7	Natural tRNA extract										+
	Hybrid-SL	+	+			+	+		+		
	IVT21-SL	+	+			+	+	+			
Figure 5<br>Figure 6	Natural tRNA extract										+
	Hybrid-SL	+	+			+	+		+		
	Hybrid-SL Δ$tRNA^{Ser}$(GAG)		+			+	+		+		
	Hybrid-SL Δ$tRNA^{Leu}$(GGA)	+				+	+		+		
Supplementary Figure 6	Natural tRNA extract										+
	Hybrid-Std + additional tRNAs (*1)			+	+	+			+		
Supplementary Figure 8<br>Supplementary Figure 12<br>Supplementary Figure 14<br>Supplementary Figure 38	Natural tRNA extract										+
	Hybrid-SL	+	+			+	+		+		
Supplementary Figure 10	Hybrid-SL	+	+			+	+		+		
	Hybrid-SL (Rosetta2)	+	+			+	+			+	
Supplementary Figure 33	Natural tRNA extract										+
	Natural tRNA extract + chimeric $tRNA^{Ser, Lue}$	+	+								+

(*1) See figure for the used additional tRNAs

Supplementary Table 7. Compositions of the cell-free translation systems used in this study. Source data are provided as a Source Data file.

	Transration systems carrying		Units
	E. coli natural tRNA extract or hybrid tRNA set	IVT21-SL tRNA set	
Hepes-K (pH 7.6)	50		mM
Potassium acetate	100		mM
Magnesium acetate	12	17.6	mM
Spermidine	2		mM
DTT	1		mM
ATP	2		mM
GTP	2		mM
CTP	1		mM
UTP	1		mM
Creatine phosphate	20		mM
10-HCO-H4folate	0.1		mM
AlaRS	0.73	1.5	µM
ArgRS	0.03	0.06	µM
AsnRS	0.38	0.76	µM
AspRS	0.13	0.26	µM
CysRS	0.02	0.04	µM
GlnRS	0.06	0.12	µM
GluRS	0.23	2.8	µM
GlyRS	0.09	0.18	µM
HisRS	0.02	0.04	µM
IleRS	0.4	2.8	µM
LeuRS	0.04	0.08	µM
LysRS	0.11	0.22	µM
MetRS	0.03	0.06	µM
PheRS	0.68	1.4	µM
ProRS	0.16	0.32	µM
SerRS	0.04	0.08	µM
ThrRS	0.09	0.18	µM
TrpRS	0.03	0.06	µM
TyrRS	0.02	0.04	µM
ValRS	0.02	0.04	µM
Elongation factor Tu/Ts	20	70	µM
Elongation factor G	0.26		µM
Initiation factor 1	2.7		µM
Initiation factor 2	0.4		µM
Initiation factor 3	1.5		µM
Release factor 2	0.25		µM
Release factor 3	0.17		µM
Methionyl-tRNA formyltransferase	0.6		µM
Ribosome recycling factor	0.5		µM
Creatine kinase	4		µg/mL
Adenosine kinase	0.1		µM
Inorganic pyrophosphatase	0.1		µM
Nucleoside-diphosphate kinase	0.1		µM
Ribosome	1.2		µM
Peptidyl-tRNA hydrolase	0	0.1	µM
DnaJ	0.5	0	µM
DnaK	1.2	0	µM
GrpE	0.5	0	µM

Supplementary references

1. Fujino T, Tozaki M, Murakami H. An Amino Acid-Swapped Genetic Code. *ACS Synth. Biol*. **9**, 2703-2713 (2020).
2. Ehrenstein G. Isolation of sRNA from intact *Escherichia coli* cells. *Meth. Enzymol*. **12,** 588-596 (1967).
