# Supplementary material for: Ser/Leu-swapped cell-free translation system constructed with natural/in vitro transcribed-hybrid tRNA set
Source: Nat Commun. 2024 May 16;15:4143. doi: 10.1038/s41467-024-48056-z (PMC11099018; doi:10.1038/s41467-024-48056-z)
Supplement: Supplementary file 3 — Description of Additional Supplementary Files [file 41467_2024_48056_MOESM3_ESM.pdf]

Description of Additional Supplementary Files

File Name: Supplementary Data 1
Description: Sequences of DNA templates used for the preparation of mRNAs that code for M2 and M3 mRNAs.

File Name: Supplementary Data 2
Description: Sequences of DNA templates used for the preparation of mRNAs that code for model proteins employed in this study.

File Name: Supplementary Data 3
Description: Primer sequences used to prepare the template DNA for synthesizing M1 mRNAs.

File Name: Supplementary Data 4
Description: Pairs of primers used to prepare the template DNA for synthesizing IVT-tRNAs.

File Name: Supplementary Data 5
Description: Primer sequences used to prepare the template DNA for synthesizing IVT-tRNAs.

File Name: Supplementary Data 6
Description: List of the samples with FASTA files and data files in LC-MS/MS analysis.
