# Supplementary material for: Ser/Leu-swapped cell-free translation system constructed with natural/in vitro transcribed-hybrid tRNA set
Source: Nat Commun. 2024 May 16;15:4143. doi: 10.1038/s41467-024-48056-z (PMC11099018; doi:10.1038/s41467-024-48056-z)
Supplement: Supplementary file 10 — Reporting Summary [file 41467_2024_48056_MOESM10_ESM.pdf]

nature portfolio

Corresponding author(s): Hiroshi Murakami

Last updated by author(s): 2024-4-13

# Reporting Summary

Nature Portfolio wishes to improve the reproducibility of the work that we publish. This form provides structure for consistency and transparency in reporting. For further information on Nature Portfolio policies, see our Editorial Policies and the Editorial Policy Checklist.

## Statistics

For all statistical analyses, confirm that the following items are present in the figure legend, table legend, main text, or Methods section.

| n/a | Confirmed | |
|---|---|---|
| ☐ | ☒ | The exact sample size (*n*) for each experimental group/condition, given as a discrete number and unit of measurement |
| ☐ | ☒ | A statement on whether measurements were taken from distinct samples or whether the same sample was measured repeatedly |
| ☐ | ☒ | The statistical test(s) used AND whether they are one- or two-sided<br>*Only common tests should be described solely by name; describe more complex techniques in the Methods section.* |
| ☒ | ☐ | A description of all covariates tested |
| ☒ | ☐ | A description of any assumptions or corrections, such as tests of normality and adjustment for multiple comparisons |
| ☐ | ☒ | A full description of the statistical parameters including central tendency (e.g. means) or other basic estimates (e.g. regression coefficient) AND variation (e.g. standard deviation) or associated estimates of uncertainty (e.g. confidence intervals) |
| ☐ | ☒ | For null hypothesis testing, the test statistic (e.g. *F*, *t*, *r*) with confidence intervals, effect sizes, degrees of freedom and *P* value noted<br>*Give P values as exact values whenever suitable.* |
| ☒ | ☐ | For Bayesian analysis, information on the choice of priors and Markov chain Monte Carlo settings |
| ☒ | ☐ | For hierarchical and complex designs, identification of the appropriate level for tests and full reporting of outcomes |
| ☒ | ☐ | Estimates of effect sizes (e.g. Cohen's *d*, Pearson's *r*), indicating how they were calculated |

*Our web collection on statistics for biologists contains articles on many of the points above.*

## Software and code

Policy information about availability of computer code

| | |
|---|---|
| Data collection | Fluorescent images of the gels in the Figure 4 and S9 were obtained by Typhoon FLA 9000 system with Tyhpoon FLA 9000 control software ver. 1.2 (GE Healthcare).<br>Fluorescent images of the gels in the Figure S11 and S12 were obtained by ChemiDoc MP with ImageLab Touch software ver. 2.4.0.03 (Bio-RAD).<br>Gel images detected by autoradiography were obtained by PharosFX Molecular Imager with Quantity One software ver. 4.6.9 (Bio-Rad).<br>A gel image with ethidium bromide staining was obtained by Gel Doc EZ system with Image Lab software ver. 6.0.1 (Bio-Rad).<br>Mass spectra were obtained by autoflex maX with flexControl software ver. 3.4 (Bruker).<br>Fluorescent spectra were measured by FP-8500 fluorescence spectrometer with Spectra manager™ II (JASCO).<br>LC-MS data was obtained by Q Exactive Hybrid Quadrupole-Orbitrap Mass Spectrometer (ThermoFisher Scientific) with Xcalibur 4.1.50 (ThermoFisher Scientific). |
| Data analysis | Fluorescent images of the gels obtained by Typhoon FLA 9000 system were analyzed using ImageQuant TL 1D software ver. 7.0 (GE Healthcare).<br>Other gel images were analyzed using Image Lab software ver. 6.1.0 (Bio-Rad).<br>Mass spectra were analyzed using flexAnalysis software ver. 3.4 (Bruker).<br>Fluorescent spectra were analyzed using Spectra manager™ II (JASCO).<br>LC-MS data was analyzed using Proteome Discoverer 2.4.1.15 (ThermoFisher Scientific). |

For manuscripts utilizing custom algorithms or software that are central to the research but not yet described in published literature, software must be made available to editors and reviewers. We strongly encourage code deposition in a community repository (e.g. GitHub). See the Nature Portfolio guidelines for submitting code & software for further information.

## Data

Policy information about availability of data

All manuscripts must include a data availability statement. This statement should provide the following information, where applicable:

- Accession codes, unique identifiers, or web links for publicly available datasets
- A description of any restrictions on data availability
- For clinical datasets or third party data, please ensure that the statement adheres to our policy

Source data for all figures and tables except LC-MS are provided with the paper. LC-MS data that support the findings of this study have been deposited to jPOST with the identifier JPST002984 (PXD050604).

## Research involving human participants, their data, or biological material

Policy information about studies with human participants or human data. See also policy information about sex, gender (identity/presentation), and sexual orientation and race, ethnicity and racism.

| | |
|---|---|
| Reporting on sex and gender | NA |
| Reporting on race, ethnicity, or other socially relevant groupings | NA |
| Population characteristics | NA |
| Recruitment | NA |
| Ethics oversight | NA |

Note that full information on the approval of the study protocol must also be provided in the manuscript.

# Field-specific reporting

Please select the one below that is the best fit for your research. If you are not sure, read the appropriate sections before making your selection.

☒ Life sciences ☐ Behavioural & social sciences ☐ Ecological, evolutionary & environmental sciences

For a reference copy of the document with all sections, see nature.com/documents/nr-reporting-summary-flat.pdf

# Life sciences study design

All studies must disclose on these points even when the disclosure is negative.

| | |
|---|---|
| Sample size | No sample size calculation was performed. Experiments for comparing the translational yields were triplicated, which is general sample size in the research field. Other experiments were performed once due to sample availability. |
| Data exclusions | No data was excluded. |
| Replication | Replication of experiments are described in figure legends. |
| Randomization | Randomization was not relevant to this study because all experiment in this study were performed by in vitro translation systems and not performed on humans or animals. |
| Blinding | Blinding was not relevant to this study because it includes no experimental approach that could introduce bias. |

# Reporting for specific materials, systems and methods

We require information from authors about some types of materials, experimental systems and methods used in many studies. Here, indicate whether each material, system or method listed is relevant to your study. If you are not sure if a list item applies to your research, read the appropriate section before selecting a response.

## Materials & experimental systems

| n/a | Involved in the study |
|---|---|
| ☒ | ☐ Antibodies |
| ☒ | ☐ Eukaryotic cell lines |
| ☒ | ☐ Palaeontology and archaeology |
| ☒ | ☐ Animals and other organisms |
| ☒ | ☐ Clinical data |
| ☒ | ☐ Dual use research of concern |
| ☒ | ☐ Plants |

## Methods

| n/a | Involved in the study |
|---|---|
| ☒ | ☐ ChIP-seq |
| ☒ | ☐ Flow cytometry |
| ☒ | ☐ MRI-based neuroimaging |
